# A proteomic view on the developmental transfer of homologous 30 kDa lipoproteins from peripheral fat body to perivisceral fat body *via *hemolymph in silkworm, *Bombyx mori*

**DOI:** 10.1186/1471-2091-13-5

**Published:** 2012-02-28

**Authors:** Britto Cathrin Pakkianathan, Nitin Kumar Singh, Muthukalingan Krishnan, Simone König

**Affiliations:** 1 Department of Environmental Biotechnology, Bharathidasan University, Tiruchirappalli, India; 2 Integrated Functional Genomics, Interdisciplinary Center for Clinical Research, University of Münster, Röntgenstr. 21, 48149 Münster, Germany

## Abstract

**Background:**

A group of abundant proteins of ~30 kDa is synthesized in silkworm larval peripheral fat body (PPFB) tissues and transported into the open circulatory system (hemolymph) in a time-depended fashion to be eventually stored as granules in the pupal perivisceral fat body (PVFB) tissues for adult development during the non-feeding stage. These proteins have been shown to act anti-apoptotic besides being assigned roles in embryogenesis and defense. However, detailed protein structural information for individual PPFB and PVFB tissues during larval and pupal developmental stages is still missing. Gel electrophoresis and chromatography were used to separate the 30 kDa proteins from both PPFB and PVFB as well as hemolymph total proteomes. Mass spectrometry (MS) was employed to elucidate individual protein sequences. Furthermore, 30 kDa proteins were purified and biochemically characterized.

**Results:**

One- and two-dimensional gel electrophoresis (1/2D-PAGE) was used to visualize the relative changes of abundance of the 30 kDa proteins in PPFB and PVFB as well as hemolymph from day 1 of V instar larval stage to day 6 of pupal stage. Their concentrations were markedly increased in hemolymph and PVFB up to the first two days of pupal development and these proteins were consumed during development of the adult insect. Typically, three protein bands were observed (~29, 30, 31 kDa) in 1D-PAGE, which were subjected to MS-based protein identification along with spots excised from 2D-gels run for those proteomes. Gas phase fragmentation was used to generate peptide sequence information, which was matched to the available nucleotide data pool of more than ten highly homologous insect 30 kDa lipoproteins. Phylogenetic and similarity analyses of those sequences were performed to assist in the assignment of experimentally identified peptides to known sequences. Lipoproteins LP1 to LP5 and L301/302 could be matched to peptides extracted from all bands suggesting the presence of full length and truncated or modified protein forms in all of them. The individual variants could not be easily separated by classical means of purification such as 2D-PAGE because of their high similarity. They even seemed to aggregate as was indicated by native gel electrophoresis. Multistep chromatographic procedures eventually allowed purification of an LP3-like protein. The protein responded to lipoprotein-specific staining.

**Conclusions:**

In *B. mori *larvae and pupae, 30 kDa lipoproteins LP1 to LP5 and L301/302 were detected in PPFB and PVFB tissue as well as in hemolymph. The concentration of these proteins changed progressively during development from their synthesis in PPFB, transport in hemolymph to storage in PVFB. While the 30 kDa proteins could be reproducibly separated in three bands electrophoretically, the exact nature of the individual protein forms present in those bands remained partially ambiguous. The amino acid sequences of all known 30 kDa proteins showed very high homology. High-resolution separation techniques will be necessary before MS and other structural analysis can shed more light on the complexity of the 30 kDa subproteome in *B. mori*. A first attempt to that end allowed isolation of a *B. mori *LP3-like protein, the complete structure, properties and function of which will now be elucidated in detail.

## Background

Silkworm is the larva of the domesticated silk moth, *Bombyx mori*. It is of great interest to research not only due to its economic importance in silk production [1], but silkworm also serves as a model organism in insect science because of its large size and ease of culture [2,3]. Accelerated by the publication of the full genome [4,5] silkworm genomics and proteomics have quickly proceeded [2,3,6-8], in particular, because it is possible, due to the size of the insect, to separate silkworm organs at different developmental stages and study them individually (for an overview on the proteomics efforts in that respect see ref. [2]).

Silkworm larvae advance through five stages of growth called instars (I instar: 3-4 days of growth, II instar: 2-3 days, III instar: 3-4 days, IV instar: 3-5 days, V instar: 6-8 days). At the end of the V instar, larvae stop feeding and spin silk to form a cocoon in which they pupate (non-feeding/silk-spinning stage: 7-8 days) [2]. In silkworm, two differentiated fat body (FB) tissues are known by their embryonic origin [9,10]. FB tissue, which is situated at the epidermis or integument region, is called peripheral (PP) or larval FB tissue and it is also known as synthesis site. Most of the major hemolymph proteins are synthesized by PPFB tissue and are secreted into hemolymph. FB tissue, which is situated in the visceral compartment, is called perivisceral (PV) or pupal FB tissue and it is structurally and functionally different from PPFB [9,10]. The important function of PVFB tissue is to sequester the storage proteins and 30 kDa proteins, which are synthesized in large quantities during the final larval stadium, from the hemolymph, and store them as granules for adult development during the non-feeding stage [11-13]. Insect FB is often compared to vertebrate liver and adipose tissue [14,15].

Proteome analysis of total FB tissue (V instar, day 3) assigned as many as 380 proteins [16]. However, major hemolymph proteins such as lipophorin [17], vitellogenin, storage protein, egg specific protein and 30 kDa proteins, which were also found in FB tissue, drew the most attention in the past as they are needed for silkworm growth, development and reproduction [11,18-20]. They are synthesized in larval FB tissues, in particular that of the final instar larva, and are transported into the open circulatory system, the hemolymph, by receptor-mediated endocytosis to attain their respective tissues in a time-depended fashion during post embryonic development and metamorphosis [7,13,21-24].

Highly abundant at some developmental stages in hemolymph as well as FB tissues is the group of proteins of approximately 30 kDa. They have been characterized as lipoproteins and accumulate towards the later stage of the IV instar larva [25,26]. The mRNA of the 30 kDa proteins increases simultaneously with the disappearance of juvenile hormone in hemolymph and concentrates in FB [27]. In fact, it has been demonstrated also on the protein level that the 30 kDa lipoproteins are synthesized in PPFB of both sexes, are secreted into the hemolymph and subsequently are moved into PVFB as 'storage protein' for adult development [28]. These proteins have been assigned multiple functions in *B. mori *and other lepidopteran insects such as anti-apoptotic properties [28-31] and roles in embryogenesis [32]. In sexually mature female moths, 30 kDa proteins are transported into yolk granules, where they become the second major yolk protein of the eggs after vitellin [33,34]. During embryonic development, the 30 kDa proteins are also detectable and eventually vanish after larval hatching [7,32]. In the pharate adult stage, the amount of 30 kDa proteins decreases sharply in female hemolymph providing nutrition for the activities of the last stages of embryonic development [7]. A protease for digesting 30 kDa proteins, named 30 kP protease A, was found in silkworm eggs. It selectively hydrolyzes the 30 kDa yolk protein, which exhibits a high expression level in late-pupal stages [34]. Hou et al. [7] suggested that during pupal-adult development, some 30 kDa proteins in male pupa are enzymatically degraded, producing nutrition for the activities of the last stages, such as eclosion and mating. Conclusively, the stored granules of 30 kDa proteins in the PVFB tissues are solubilized by enzymatic digestion or pH regulation and are transported to respective tissues *via *hemolymph. 30 kDa proteins have been shown to bind glucose and glycans suggesting their involvement in the insect self defense system [35,36] and lipid transport in the larvae of silkworm [31]. They were identified as lipoproteins [25]. Yang et al. [37] revealed that FB Bmlp7 plays a crucial role in the protection of *B. mori *against invading pathogens due to the presence of a putative sugar binding domain. Crystal structures of 30 kDa proteins LP2 (P09335) [38] and Bmlp7 [26,37] indicated an N-terminal lipid-binding cavity and a C-terminal sugar-binding site. The 30 kDa protein 6 G1 (Bmlp1) from FB was reported to interfere with hyphal growth of the entomopathogenic fungus, *Paecilomyces tenuipes *[35]. Also, 30 kDa proteins are involved in translocating chymotrypsin inhibitor-8 to the membrane of the midgut [39].

Generally, 30 kDa proteins are multifunctional and permanently required by insects; they therefore exist at various sites throughout the life cycle of the silkworm. During larval development, they are expressed in FB tissues and hemolymph [11]. They are observed in ovarioles and FB and are released into hemolymph in the pupal developmental period [32,40], where they act as storage protein and microvitellogenin [13,41]. In the adult, they are found in FB tissues, egg and hatched larvae. They have also been reported in *Manduca sexta *[42] and *Spodoptera litura *[43].

Based on the analysis of the primary sequences deduced from nucleotide data [44] and expressed sequence tags (ESTs), ten genes of 30 kDa proteins of the silkworm (Bmlp1-10) were identified and grouped into three subfamilies [26]. Research groups have also supplied nucleotide sequences for several related proteins of this approximate molecular weight. A summary of the sequences currently available in the UniProt database is given in supplementary information (see Additional file 1). As Sun et al. [26] suggested, the 30 kDa protein gene may have a common origin and may have arisen through gene duplication. It was also pointed out that it is not clear yet how much of the sequence variability present in the database is due to the use of different strains of *B. mori *[45], because the composition of the 30 kDa proteins was found to be different among strains and races [33].

No comprehensive analysis on the protein level has been carried out so far, apart from first assignments of short terminal stretches of a hemolymph 30 kDa component by Edman sequencing (matching 17 amino acids to LP3 (P09336)/L301 (Q00802) [28]) and its detection in global proteome analyses of hemolymph [46] and total FB [16]. However, RNA and protein levels hardly correlate so that detailed proteomic investigations are adamant for functional studies.

In this work, we extended the investigations to include separately prepared PPFB and PVFB tissues, respectively, of strain Tamil Nadu White X NB4D2. We used mass spectrometry (MS) and its peripheral proteomic technologies to investigate in particular the 30 kDa proteins. One- and two-dimensional polyacrylamide gel electrophoresis (1/2D-PAGE) were employed to characterize proteome changes in FB and hemolymph during larval-to-pupal transformation. We also made an effort to isolate and characterize individual 30 kDa protein forms from PVFB tissue (3^rd ^day of V instar larva) by extensive chromatographic means for detailed biochemical characterization.

## Methods

### Insects

Disease free eggs of *B. mori *(the crossbreed race, Tamil Nadu White X NB4D2) were provided from the Government grainage center, Tiruchirappalli, South India, and maintained at a temperature of 27 ± 2°C and a relative humidity of 75 ± 5% [47]. Hatched larvae were fed with chopped tender leaves of the mulberry variety (MR_2_) until III instar and with coarse leaves until the end of the last instar. Bed cleaning and spacing of larvae in trays were performed as described [11].

### Hemolymph

It was collected from 1^st ^day of V instar to 6^th ^day of pupae by making a slit in a proleg. Hemolymph that flowed from the wound without external pressure was collected in an Eppendorf tube which was pre-rinsed with phenylthiourea solution to prevent tyrosinase activity. Samples were centrifuged at 10.000 rpm at 4°C for 10 min to remove hemocytes and cell debris and stored at -80°C until further use.

### Fat body

PPFB tissue was collected from 1^st ^day of V instar to 1^st ^day of pupa and PVFB tissue from 1^st ^day of V instar to 6^th ^day of pupa. The tissue was washed with insect ringer solution containing antibiotics (0.03 mg/ml Penicillin and 0.1 mg/ml Streptomycin) followed by rinsing in ice cold phosphate buffered saline (PBS; 4.3 mM Na_2_HPO4 × 7 H_2_O, 1.4 mM NaH_2_PO_4_, 137 mM NaCl and 1 mM phenylmethylsulfonyl fluoride, pH 7.2) to completely remove hemolymph. The tissue was homogenized in a pre-chilled glass homogenizer (Borosil, India) with 200-300 μl PBS and centrifuged at 10.000 rpm at 4°C for 10 min. Phase separation was observed and the protein solution underneath the lipid layer was collected and stored at -80°C.

### Protein extraction

Lipid and contaminants were removed from the protein extract according to the protocol by Wessel and Fugge [48] with slight modifications. Briefly, 250 μl of protein extract was treated with 1 ml of 100% methanol, 250 μl of chloroform and 750 μl of milliQ water, it was vortexed and centrifuged (11.000 rpm, 5 min, 4°C). The top aqueous layer was discarded, 1 ml methanol was added to the remaining solution, which was vortexed and centrifuged (10.000 rpm, 5 min, 4°C). The resulting pellet was air dried and re-solubilized in 200 μl lysis buffer (7 M urea, 2 M thiourea, 2% CHAPS, 1% DTT, 0.8% of 40% Biolyte (pH 3-10); 10 min on ice). For improved solubilization the sample was sonicated at 50%, 60% and at 80% amplitude for 3 min, respectively.

### Gel electrophoresis

Sodium dodecyl sulfate (SDS)-PAGE was performed according the method of Laemmli [49] using Proviga (India) maxi electrophoretic apparatus. A 12% linear resolving gel and 3% stacking gel were used to separate the proteins. Native gel electrophoresis was carried out without SDS. The protein concentration was determined using the method of Bradford [50]. Loading buffer was added and the sample was heated to 95°C for 3 min. Per well 40 μg protein were loaded. SDS-PAGE was run until the bromophenol blue front reached the end of the gel (at 30 V for stacking gel, 50 V for separating gel). 2D-PAGE using Bio-Rad equipment was performed as recommended by the manufacturer using non-linear IPG strip pH 3-10 and 12% gels.

### Gel staining

Gels were stained with Coomassie Brilliant Blue R-20 solution (1 mg dye, 250 ml methanol, 35 ml acetic acid made up with water to 500 ml) for 6 h. Destaining was carried out in 50 ml distilled water containing 10 ml glacial acetic acid and 5 ml methanol. Alternatively, proteins were visualized using silver staining according to ref. [51]. Proteins were fixed exposing the gel to a solution containing 50% ethanol, 12% acetic acid and 0.1% formaldehyde overnight. Gels were then washed twice with 50% ethanol and treated with sodium thiosulfate for 1 min followed by washing in water twice. Subsequently, gels were immersed in 0.2% silver nitrate containing 0.05% formaldehyde for 20 min. Reduction of silver nitrate to metallic silver and enhancement of color was performed in the developer solution (Na_2_CO_3_, 60 g/l; 0.5 ml/l of 37% HCHCO_3_, 4 mg/l Na_2_S_2_O_3 _× 5 H_2_O). The reaction was stopped after brown protein bands appeared on yellow background.

### Western blot using vitellogenin antibody

Proteins of PVFB tissue sample (V instar 4^th ^day) were separated by 1D-PAGE and electro-transferred from the gel onto nitrocellulose membrane for 30 min at 30 V (Trans-Blot Semi-Dry, Bio-Rad) according to Towbin et al. [52]. Blots were traced with primary antibody (1 μl of primary antibody diluted with 999 μl of Tris buffered saline TBS, pH 7.5) against vitellogenin of *Pteromalus puparum *in mouse (generous gift from Prof. Gong-Yin Ye; State Key Laboratory of Rice Biology, Institute of Insect Sciences, College of Agriculture and Biotechnology, Zhejiang University, Hangzhou, China). Excess antibody was removed by washing twice with TBS (10 mM Tris base, 150 mM NaCl, pH 7.5 with 0.05%/Tween 20) for 10 min each with gentle shaking. The primary antibody was then probed with secondary antibody (goat anti mouse IgG tagged with hydrogen peroxidase, Genei Bangalore, India) and antibody binding was visualized by 4-chloro-1-naphthol/hydrogen peroxide liquid chromogenic substance.

### Lipoprotein staining

Gels were incubated in a saturated solution of the lipoprotein-specific stain Oil red O [53] (Himedia, India) in 50% methanol v/v containing 10% trichloroacetic acid w/v at 60°C for 2 h. Lipoproteins appeared as orange red bands. Gels were kept in 7% acetic acid v/v and were photographed using reflected light.

### Ion exchange chromatography

DEAE cellulose (Sigma, USA) was equilibrated with equilibration buffer (120 mM Tris HCl, pH 8.8) for 2 h. Then the cellulose was packed into a glass column (2 cm × 22 cm; Brosil, India). Crude fat body protein sample (4 mg) was loaded and the column was washed with the equilibrium buffer for 2 h to ensure that unbound proteins were removed. Bound proteins were eluted using a salt gradient (0.5 M NaCl in equilibrium buffer) at a flow rate of 1 ml/3 min. A total of 200 fractions was collected and their protein concentration was determined by the Bradford method [50]. The samples (15 μg protein each) were subjected to 12% SDS-PAGE and gels were silver stained. The samples showing only three bands (fractions 12-37) of the 30 kDa proteins were pooled and stored at -80°C for further analysis.

### Gel permeation chromatography

Gel filtration was carried out according to ref. [54] using Sephadex G-50 (Sigma, USA). The matrix was soaked in the equilibration buffer (120 mM Tris HCl, pH 8.8) and the swollen matrix was packed into the column (1 × 120 cm). The pooled fractions from the DEAE-cellulose ion exchange chromatography were lyophilized and redissolved in 120 mM Tris HCl, pH 8.8. Approximately 2 mg of total protein was loaded onto the column. Proteins were eluted using equilibration buffer. Fractions were collected at a flow rate of 2 ml/3 min. Again, the protein content of the fraction was controlled by 1D-PAGE. Fractions containing only 30 kDa proteins were stored at -80°C until further use.

### Field emission scanning electron microscopy (FE-SEM)

PVFB was collected from 3^rd ^day of V instar and fixed with 2.5% glutaraldehyde buffered with 0.1 M sodium cacodylate (pH 7.2) containing 0.1 M sucrose at 4°C for 1 h. It was washed and analyzed at SASTRA University, Thanjavur, Tamil Nadu, India using a JSM 6701 F scanning electron microscope (JEOL, Japan).

For the examination of the structure of purified protein with the same microscope, negative staining with uranium acetate [55] was used. After ion exchange purification, desalted 100 μg purified protein (pool of 3-band fractions) was dissolved in uranium acetate solution (1% uranium acetate in distilled water, pH 4.8) and lyophilized overnight. The dry protein sample was placed on a sample holder, coated with platinum and examined.

### LC-MS/MS and data analysis

Bands or spots of interest were excised from the gels and subjected to tryptic protein digestion as described [56]. Peptides were extracted from the gel matrix, dried and resolubilized in 5-10 μl of LC aqueous solvent A (0.1% formic acid, 5% acetonitrile in HPLC water) depending on the staining intensity of the original gel sample. Accordingly, 1- 4.5 μl sample was injected to the LC column (1.7 μm BEH130 C18, 100 μm × 100 mm) for nanoUPLC-MS/MS and MS^E ^with Q-TOF Premier coupled to Acquity nanoUPLC (all from Waters. Corp.). Gradients were run with aqueous solvent A and organic solvent B (0.1% formic acid, 5% water in LC acetonitrile). For UPLC-MS^E^, a gradient was run from 97% A to 50% B in 90 min using a flow rate of 0.4 μl/min; in data-dependent LC-MS/MS the gradient was 30 min long. Peptides were analyzed in the mass range *m/z *50-1800 (0.95 s scan time, 0.05 s interscan delay) alternating the collision energy from 4 V to a ramp of 10-35 V. Glu-fibrinopeptide was used as lock mass. The lock mass channel was scanned for 1 s with a frequency of 15 s while the collision energy was raised to 21 V to allow recalibration on the doubly-charged parent ion and singly-charged fragment ions. Experiments were always followed by blank runs to control removal of all material from the chromatographic column and avoid cross-talk. Data were analyzed using PLGS 2.3 (Waters Corp.) and Mascot (Matrix Science Inc.) using both the comprehensive public databases (NCBI, UniProt) as well as species-specific databases downloaded for Bombicoidae and Bombyx at irregular intervals starting June 1, 2010.

## Results and discussion

**Figure 1:**
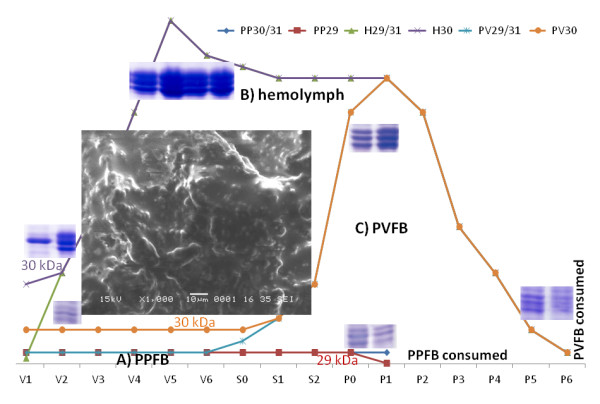
**1D-PAGE (12%) electrophoretic profiles of proteins collected from silkworm *B. mori *at different developmental stages**. The schematic provides a simplified overview of the changes in relative protein concentration as estimated by staining intensity in bands of 29, 30 and 31 kDa; the corresponding original gel data are provided in supplementary files for closer inspection. For detailed explanations see text. 50 μg per lane of protein was loaded. The insert shows an FE-SEM image of native PVFB tissue from 3^rd ^day of V instar; magnification 1.000. V1-V6: Day 1 to day 6 of V instar larval stage; S0-S2: Day 0 to day 2 of spinning stage; P0-P6: Day 0 to day 6 of pupal stage; M: Molecular weight marker. A) PPFB, larval and spinning stages. 30 kDa proteins are synthesized and secreted into hemolymph (see Additional file 2). B) Hemolymph, larval, spinning and pupal stages. 30 kDa protein concentration increases during synthesis and decreases with uptake into PVFB (see Additional file 3). C) PVFB, larval, spinning and pupal stages. 30 kDa protein concentration increases during uptake and storage and decreases during late pupal stages and transformation to adult (see Additional file 4).

In order to visualize the changes in expression of the 30 kDa proteins in larval PPFB and PVFB as well as in hemolymph, the proteomes isolated from 1^st ^day of V instar larvae to 6^th ^day of pupae were separated by 1D-PAGE. During this experimental period silkworm undergoes dramatic morphological transition, pupation and eclosion. A schematic summarizes the detected relative concentration changes of the proteins in the 30 kDa region as evaluated from the staining intensity (Figure 1, for best understanding first see corresponding source data in Additional file 2 Additional file 3 and Additional file 4). The gel images of PPFB tissue showed consistent presence of 30 kDa proteins from day 1 of V instar larvae to day 1 of pupae (Figure 1A, see Additional file 5). The three major 30 kDa proteins (29, 30, 31 kDa) reproducibly appeared and several minor bands were also distinguished in the molecular weight region from 25-35 kDa. The result reflects the continuous production of 30 kDa proteins in PPFB and their release into hemolymph.

After day 1 of pupa, PPFB tissue cannot be distinguished anymore as a result of insect development. It was routinely observed in the laboratory that the 29 kDa band tends to weaken in intensity or even vanish at this stage. In the data shown here, the effect is not obvious (only slightly lower intensity compared to the 30 and 31 kDa bands), but it was evident when sampling was performed with higher frequency (e.g. every 2 h instead of daily, data not shown). It is of note that no protein could be assigned by MS analysis in that particular band (see Table 1). The substances forming the 29 kDa band are currently under investigation in our laboratory with respect to apoptosis.

**Table 1:** Evidence for lipoproteins LP1 to LP5 and L301/L302 in 1D-PAGE bands based on unique peptides on days 0-5 of pupa (see Additional file 5 and Additional file 6)

Day	LP	0	0	0	1	1	1	5	5	5
**Band kDa**		**29**	**30**	**31**	**29**	**30**	**31**	**29**	**30**	**31**

**PPFB**	**1**	x								

	**2**		x	x						

	**G3**		x	x			x	x		

	**G4**	x	x			x				

	**5**		x	x		x	x			

**PVFB**	**1**		x		x	x	x	x	x	

	**2**	x		x	x	x		x	x	x

	**G3**			x		x	x	x	x	x

	**G4**	x	x		x	x	x	x	x	x

	**5**	x	x	x	x	x	x	x	x	x

**Hemolymph**	**1**				x	x	x	x	x	

	**2**					x	x	x	x	x

	**G3**					x	x	x	x	x

	**G4**				x	x	x	x	x	x

	**5**				x	x	x	x	x	x

The presence of homologous lipoproteins in some bands cannot be excluded; unique peptides may have gone undetected.

As a consequence of protein synthesis in PPFB and release into hemolymph, an increase of the concentration of the 30 kDa proteins in hemolymph could be observed (Figure 1B, see Additional file 3). It reached the maximum at the wandering stages (band overloading at day 5 of V instar larva) and became a major component of hemolymph during pupation. The high level of 30 kDa proteins was maintained as long as synthesis in PPFB was ongoing. From day 2 of pupa the 30 kDa protein level decreased. This process coincided with the gradual decrease of 30 kDa protein concentration in PVFB. During feeding cessation, 30 kDa proteins were taken up from the hemolymph by PVFB tissue, the main tissue filling the body cavity of *B. mori*. Healthy tissue consists of bulge-like structures (see electron micrograph image in Figure 1) while, during apoptosis, empty vacuoles can be observed [57].

Interesting is the detection of a higher staining intensity of the 30 kDa band compared to the 29 and 31 kDa bands in hemolymph day 1 of V instar and in PVFB during the whole larval period indicating overabundance of a major protein form. As will be discussed below, however, all LP1 to LP5 lipoprotein sequences can be detected in that band, as in the other two bands, so that it is not clear at this point, which process is responsible for the effect. Such detailed observations have not been possible before; but in general above results on developmental changes of 30 kDa proteins in FB and hemolymph agree with data by other authors (within limits due to sample origin and preparation as well as gel electrophoresis settings) [6,13,26]. Kim et al. [28] and Hou et al. [7] reported four or five individual protein bands for the 30 kDa proteins, but it was noticed that only three forms of 30 kDa proteins were present at considerable amounts in FB tissues. An earlier report of Gamo [25] stated that the three major forms of 30 kDa proteins accounted for 35% of total hemolymph protein.

Considering the homology of 30 kDa lipoproteins to *M. sexta *vitellogenin, a female-specific lipo-glyco-carenoprotein, it was not surprising that they showed cross-reactivity with its monoclonal antibody (see Additional file 4) [55,58]. After 1D-PAGE separation of PVFB, the response of a protein at ~29 kDa was noted in Western blot. However, none of the related 30 kDa proteins discussed here (see Additional file 1 Additional file 7) including microvitellogenin from *M. sexta *(P19616, Q0VJU3) exhibited the vitellogenin domain profile (PROSITE scan on PDOC51211). Nevertheless, the 30 kDa proteins have been shown to be involved in yolk development like vitellogenin [32,33] accounting for about 35% of total yolk proteins [59]. They are sequestered by developing oocytes along with vitellogenin and egg specific protein and are stockpiled as yolk granules in eggs providing nutrient for embryo development [32]. It is known that the 30 kDa proteins do not use the same endocytotic receptor as vitellogenin [33]. In female silkworm they are involved in embryonic development whereas males use them in insect metamorphosis and mating [7,32].

**Figure 2:**
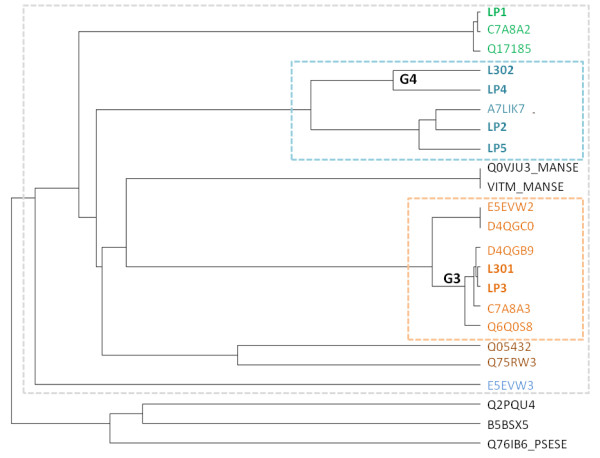
**Phylogenetic tree of insect lipoproteins similar to LP1**. Generated using Mafft (v6.857b; http://mafft.cbrc.jp). For details see Additional file 7 Additional file 8 Additional file 9 Additional file 10 Additional file 11 Additional file 12 and Additional file 13. All sequences are from *B. mori *unless otherwise noted (MANSE - *M. sexta*, PSESE - *P*. *separata*). Grouping of related sequences is emphasized by boxes.

We proceeded with the identification of the proteins in the bands at 29, 30 and 31 kDa at different developmental stages of pupa (day 0, 1, 5) in FB tissues and hemolymph by MS. The analysis of sequence data obtained by gas phase fragmentation during MS analysis of tryptic peptides relies on their *in silico *comparison to known protein sequences in the public domain. In case of unknown proteins with no database entry, protein identification can become increasingly difficult. Comparison with homologous proteins may assist, but most often *de novo *sequencing is required. In order to evaluate the current database situation for sequence information for the 30 kDa proteins, we performed a UniProt BLAST search for lipoprotein LP1 of *B. mori *(UniProt accession P09334). Twenty-three sequences with a high degree of similarity were identified (≥ 40% identity; see Additional file 7 and Additional file 8) including two sequences from *M. sexta *and one from *Pseudaletia separata*. The homology of *B. mori *30 kDa proteins and microvitellogenin from the tobacco moth (Q0VJU3, P19616) has been known for some time, while the so called growth-blocking peptide-binding protein from the oriental armyworm (Q76IB6) is a relatively new addition to the insect 30 kDa lipoprotein family. It was noted that some sequences such as C7A8A2 and Q17185 were identical except for one or two amino acid residues (see Additional file 9 Additional file 10 and Additional file 11). Coloring the remaining sequences by their similarity to the seven lipoproteins first described (LP1 to LP5 [22] and L301/L302 [23]; see Additional file 12) revealed a clear grouping of primary protein structures visualized in the phylogenetic tree shown in Figure 2. Lipoprotein LP1-LP5 sequences represented characteristic individual proteins, while L301 was strongly related to LP3 (the group of proteins was named G3) and L302 to LP4 (named G4).

**Figure 3:**
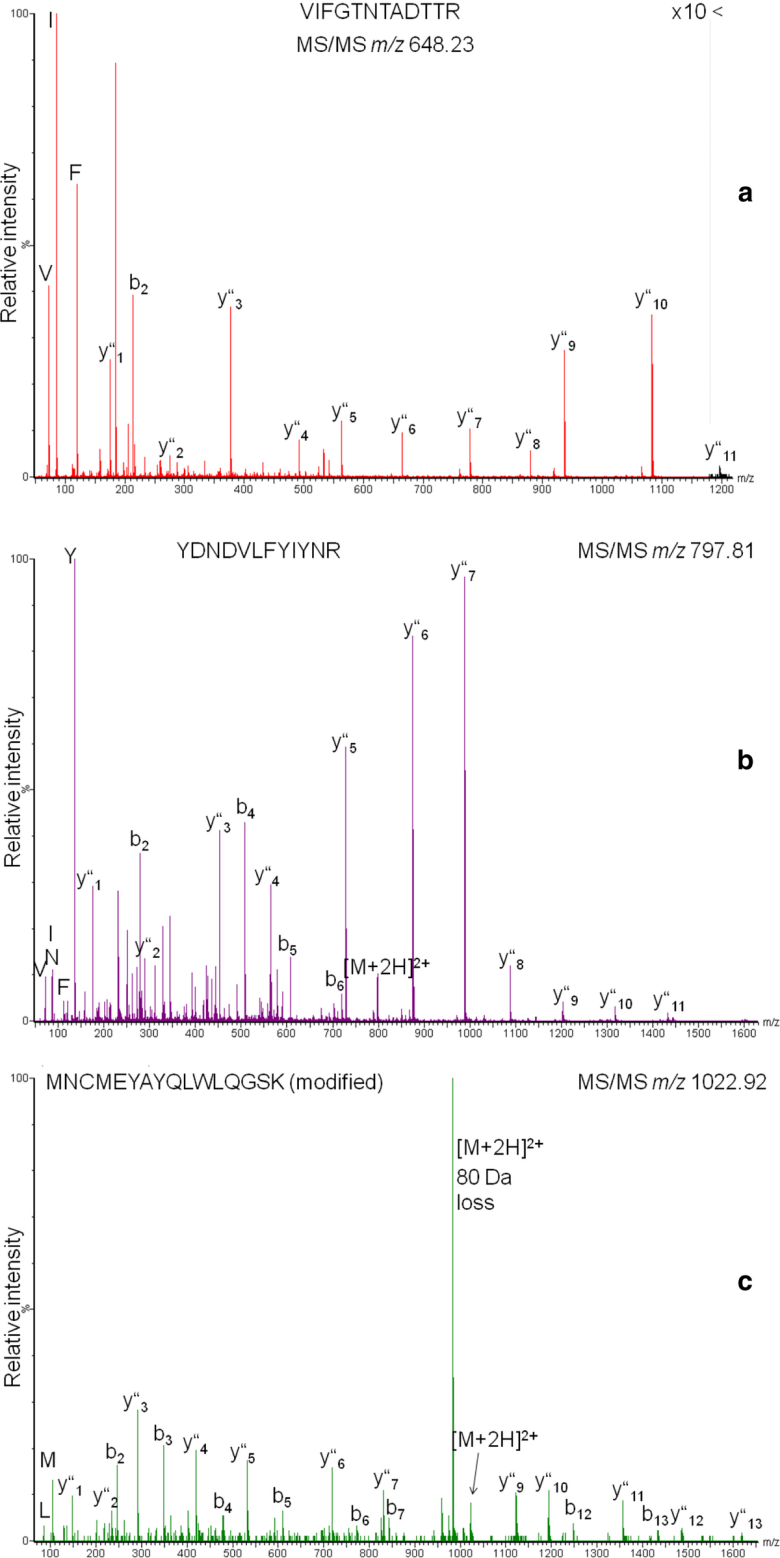
**MS/MS spectra of peptides unique for specific 30 kDa lipoproteins**. Fragment ion series from the N- and C-terminus were observed as well as immonium ions. A) L302. B/C) from purified lipoprotein (fraction 60, Fig. 4). B) LP3/L301, C) LP3/L301, modified (phosphorylated or O-sulfonated).

**Figure 4:**
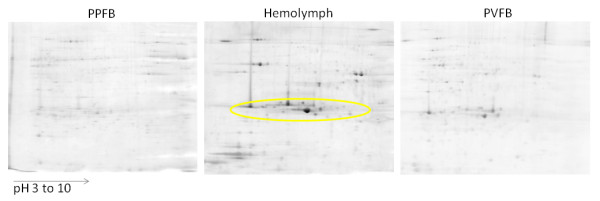
**2D-PAGE separation of hemolymph and FB protein of day 3 of V instar larvae from silkworm *B. mori***. Coomassie images of the region 5-250 kDa, pH 3-10. The increase of proteins in the 30 kDa region (marked by ellipse) in hemolymph and PVFB is clearly evident. Protein spots in that area were excised. Assignments can be found in Additional file 15. Proteins of the 30 kDa family were detected in all spots with few exceptions.

The assignment of the MS data for pupal lipoproteins is summarized in Table 1. It is based on peptides, which are unique for certain protein sequences (for individual peptide matches see Additional file 5 and Additional file 14). An exemplary spectrum for a unique peptide of L302 is shown in Figure 3A demonstrating confident sequence determination. The Q-TOF mass spectrometer allows measurements with a resolution of ~10.000 (full width at half maximum) and mass accuracies < 10 ppm, but the assignment of amino acid residues very similar in mass such as lysine (128.095 mass units) and glutamine (128.056 mass units) may remain ambiguous. Isobaric amino acids residues like leucine and isoleucine (both 113.084 mass units) were also not distinguishable in the chosen measurement approach. None of the three bands at ~29, 30 and 31 kDa could be assigned to only one protein sequence even though they were well separated in 1D-PAGE. In fact, characteristic peptides for every single lipoprotein L1-L5 could be detected in hemolymph day 1 (bands at 30, 31 kDa) and day 5 (bands at 29 and 30 kDa). The observation that L2 and G3 were absent on day 1 in the 29 kDa band and L1 was not found in the 31 kDa band at day 5 may in principal be attributed to a lower concentration of those proteins during certain periods of development; more likely, however, are difficulties in finding unique peptides in the complex mixtures present in each band. This is especially true, since hemolymph is the link between synthesis and storage site and all lipoproteins L1-L5 have arrived in PVFB at day 5. Essentially, all lipoproteins L1 to L5 could be found in all three bands, possibly at different individual lengths or in modified forms. Obviously, 1D-PAGE technology is not sensitive enough to visualize subtle concentration changes of one of several proteins in a single band. Therefore, we hoped to achieve better separation in 2D-PAGE experiments of FB and hemolymph methanol/chloroform precipitated protein samples from final instar stadium (day 3, V instar; non-linear pI range 3-10; Figure 4). This technology separates proteins not only by molecular weight but also by their isoelectric point and it has been used to analyze silkworm hemolymph proteins, skeletal muscle, silk glands and FB (for review see [2]). Approximately 250 proteins spots were visible in the gel image for PPFB, 210 for hemolymph and 190 for PVFB. A relatively uniform staining of the spots in the PPFB image infered that many proteins were produced at the synthesis site at about equal rate. In hemolymph, however, about a dozen spots across the whole gel were more intensively stained including spots in the 30 kDa region. This pattern is almost mirrored for PVFB demonstrating again the protein flow synthesis > transport > storage. Thirty-two protein spots were excised from the gel of the hemolymph proteome in the 30 kDa region and subjected to proteomic analysis. Unfortunately, the 30 kDa proteins were distributed over an area of pI ~5-9 with low resolution (for spot assignment see Additional file 15). Lipoproteins L1 to L5 were detected in parallel in single spots, but also in different spots at varying pI. That effect was repeatedly observed although other proteins such as 27 kDa glycoprotein, juvenile hormone binding protein and 14-3-3 protein could be well separated. Possibly, functional moieties of varying size such as lipids, attached to the protein backbone, were responsible for this property of the 30 kDa proteins. It became clear in these experiments that traditional gel electrophoretic separation techniques were not suitable to properly elucidate the primary structure of these proteins.

**Figure 5:**
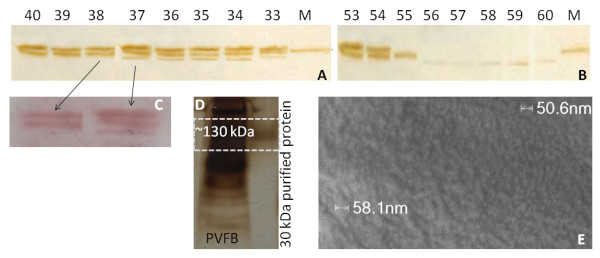
**Purification and characterization of 30 kDa proteins**. DEAE cellulose ion exchange chromatography (A) followed by Sephadex G25 gel filtration chromatography (B). The protein content for exemplary fractions (A) 33-40, (B) 53-60, was visualized using 1D-PAGE. Fractions 38-51 showed two bands in the 30 kDa region (M - 30 kDa marker; ~30, 31 kDa), fractions 12-37 three bands (~ 29, 30, 31); otherwise the gels showed no protein signals. Gel filtration allowed isolation of protein in a single band (fraction 55-60). Bands from fractions 34 (A) and 60 (B) were subjected to LC-MS/MS analysis. The samples showing only three bands of the 30 kDa proteins were pooled (PUR) and stored at -80°C for further analysis. (C) Oil red O lipoprotein staining of fractions 38 and 37 separated by 12% SDS-PAGE. (D) Crude PVFB protein was compared to PUR protein by native 7% 1D-PAGE. The latter was not resolved and detected at ~130 kDa (no marker available). (E) Electron micrograph of PUR protein, magnification 50.000.

Therefore, we made an effort to specifically isolate the 30 kDa proteins from 3 mg of total protein from PVFB (day 3 of V instar) by chromatography-based two-step purification as shown for vitellogenin isolation earlier [60]. First, DEAE cellulose anion exchange chromatography was employed to purify the 30 kDa proteins from other major proteins such as lipophorin, vitellogenin, storage proteins, egg specific protein and further low molecular proteins. Tris HCl (pH 8.2) was chosen as an equilibration buffer based on the theoretical pI of the 30 kDa proteins (pI 6.5-8; SwissProt) combined with 0.7 M NaCl salt gradient elution. Fractions 33-37 thereby showed three prominent protein bands in the 30 kDa region on 12% SDS-PAGE (Figure 5A). Silver staining was employed for improved detection of low abundant impurities and none were found. Following DEAE cellulose ion exchange chromatography by Sephadex G-50 gel filtration it was furthermore possible to reduce the protein complexity in some fractions to result in a single gel band (Figure 5B). LC-MS analysis of selected bands demonstrated that after the first purification step proteins such as 14-3-3 or prohibitin still coeluted with lipoproteins L1-L5 (peptide assignments shown in Additional file 16). Successive gel filtration then allowed isolation of predominantly LP3/L301 (judging by the number of extracted peptides, see Additional file 17). An exemplary spectrum for a peptide common to LP3 and L301 is shown in Figure 3B. There is evidence based on collision-induced dissociation that peptide MNCMEYAYQLWLQGSK is phosphorylated either at Y6, Y8 or S15 (Figure 3C) although no phosphorylation site is predicted in that region (NetPhos 2.0). However, O-sulfonation (mass difference to phosphorylation 0.0095 Da) cannot be entirely excluded. Peptides derived from LP2, LP4 and LP5 were found (Additional file 18) in addition to peptides unique for proteins Q6Q0S8 and C7A8A3. At this point it cannot be clarified whether the ambiguous sequence assignment is due to the fact that the available sequence information in the databases stems from other *Bombyx *subspecies or from co-purified protein forms.

The isolated 30 kDa proteins were characterized further, first comparing them with the total PVFB proteome in native 1D-PAGE (Figure 5D). Interestingly, they were not resolved at 30 kDa, but appeared at ~130 kDa indicating a tendency to aggregate. Staining with Oil red O after 1D-PAGE separation of the purified 30 kDa proteins showed their lipoprotein nature (Figure 5C). Formerly, Gamo [25] and Kim et al. [61] have reported that 30 kDa proteins were lipo-glycoproteins according to Sudan Black and periodic acid-Schiff staining. Lipoproteins are defined as post-translationally modified by the attachment of at least one lipid or fatty acid (UniProt definition). The detection of lipoproteins is typically performed using fat-soluble dyes such as those from the Sudan family [62]. These dyes target hydrophobic compounds like neutral triglycerides, lipids and fatty acids, but it remains unclear to what extent sequence stretches of hydrophobic amino acids respond to the dye. For redhorse vitellogenin poor staining was observed after SDS-PAGE suggesting that lipid content was reduced by the procedure [58], that free lipids were removed and that only covalently bound lipidoic moieties were present.

The desalted pooled 30 kDa protein fractions from ion exchange chromatography were furthermore subjected to field emission scanning electron microscopy (FE-SEM, Figure 5E), a technique which has been used to estimate protein size. For example, for insect vitellogenin [55] as well as silkworm vitellin [45] a diameter of roughly 13 nm was suggested. We observed nanoparticle structures of ~50 nm which were otherwise indistinguishable in size and shape. Since proteins are expected to be much smaller [55,63], the image possibly shows protein agglomerates. However, artifacts cannot be excluded; an extensive study has only recently dealt with EM negative staining artifacts due to rouleau formation investigating plasma lipoproteins [64].

Amino acid separation of hydrolyzed purified 30 kDa protein was also performed by LC (29 kDa band, see Additional file 19). Glutamic and aspartic acid as well as alanine were detected in abundance in agreement with Gamo [25], but leucine, isoleucine and lysine were not seen at all. The presence of lysine and argentine (trypsin cleavage sites) as well as that of entire peptide sequences was however already determined from the enzymatic digestion experiments used in preparation of MS analysis. Acid hydrolysis is furthermore known to deamidate glutamine and asparagines and destroy tryptophan so that this type of analysis was not too conclusive. Amino acid analysis may have possibly also been hampered by covalently bound moieties.

## Conclusion

Comprehensive structural proteomic analysis of 30 kDa proteins isolated from *B. mori *PPFB and PVFB tissues as well as hemolymph at various developmental stages allowed insights into protein primary structure along with the visualization of time-dependent changes in concentration. One- and two-dimensional gel electrophoresis was employed to resolve individual members of the 30 kDa protein family. MS protein identification showed, however, that five lipoproteins LP1 to LP5 reproducibly populate the three major bands in 1D-PAGE and could not be properly resolved using 2D-PAGE. It is of note that LP3 shares large parts of its sequence with L301 and LP4 with L302. Sequence assignment remained partially ambiguous due to differences in protein database information for different silkworm strains. These observations indicated that each of the proteins is present at different molecular weights as well as pIs so that specific or unspecific cleavage, terminal truncation and protein modification need to be considered. The 30 kDa proteins were shown to respond to lipoprotein-specific staining, also in our experiments, and to be glycoproteins (shown by other authors); the covalent addition of lipid or sugar moieties would therefore explain the electrophoretic behavior as well. In order to clarify this hypothesis along with complete sequence elucidation, isolation of each of the individual protein forms will be necessary. We have used multidimensional chromatography for this purpose and succeeded in purifying a LP3/L301-like lipoprotein. Much more effort will now be required to upscale purification to characterize each protein in detail biochemically and functionally. As soon as a single protein can be isolated at high purity, its amino acid sequence can be confidently assigned by MS despite the complex database situation containing dozens of highly similar lipoprotein sequences.

The 30 kDa proteins showed developmental fluctuation patterns similar to storage proteins in *B. mori *and other insects such as *M. sexta *[65], *Lymantria dispar *[66], *M. brassicae *[67], *Helicoverpa zea *[68], *S. litura *[69], *Apis mellifera *[70] and *Amsagta albistriga *[71] as visualized using gel electrophoresis. The notable increase of 30 kDa protein in PVFB tissues during the spinning and early pupal stages indicated that the in PPFB abundantly synthesized 30 kDa proteins during the vigorous feeding stage were taken up by and stored in PVFB (storage tissue) to provide the nutrients for the insects at non-feeding stages. This observation is in accord with Mine et al. [13], who have reported that the 30 kDa proteins are a specific type of plasma protein and one of the so-called "storage proteins" in the silkworm hemolymph being synthesized in FB of the feeding larvae and released into the hemolymph. During the larval-pupal transformation, they are transported from the silkworm hemolymph to FB cells and stored as protein granules. Insects prepare for synthetic demands of molting, metamorphosis and reproduction by accumulating the proteins in their hemolymph during the active feeding period [11,72-75]. In insects, juvenile hormone regulates the biosynthesis of major plasma proteins [76].

Parameters such as strain-specific properties, choice of experimental tissue, developmental stages and biochemical protein modifications determine the diversity among lipoprotein sequences and complicate their analysis. Current sequence assignment remained partially ambiguous due to multiple database entries for very similar insect lipoproteins. In addition to their high homology, 30 kDa lipoproteins seemed to form high molecular weight aggregates as detected by native 1D-PAGE. They co-purified and were difficult to separate by common electrophoretic and chromatographic methods.

As a prerequisite for functional investigations, future research will have to elucidate the entire primary amino acid sequence of each 30 kDa protein member and development-related structural changes such as truncation and cleavage as well as modifications such as lipidation, glycosylation, phosphorylation.

## Competing interests

The authors declare that they have no competing interests.

## Authors' contributions

BCP and NS raised silkworms, performed protein isolation and biochemistry and wrote experimental parts; MK oversaw this work. SK performed proteomic and bioinformatic analysis and wrote the paper. All authors read and approved the final manuscript.

## Supplementary Material

Additional file 1 **Database and sequence references for *B. mori *lipoproteins**. Click here for file

Additional file 2 **1D-PAGE (12%) electrophoretic profiles of protein collected from silkworm *B. mori *PPFB at larval and spinning stages**. Data for Figure 1A. 30 kDa proteins are synthesized and secreted into hemolymph. M: Molecular weight marker; V1-V6: Day 1 to day 6 of V instar larval stage; S0-S2: Day 0 to day 2 of spinning stage; P0-P6: Day 0 to day 6 of pupal stage. Click here for file

Additional file 3 **1D-PAGE (12%) electrophoretic profiles of protein collected from silkworm *B. mori *hemolymph at larval, spinning and pupal stages. Data for Figure **1B. 30 kDa protein concentration increases during synthesis and decreases with uptake into PVFB. M: Molecular weight marker; V1-V6: Day 1 to day 6 of V instar larval stage; S0-S2: Day 0 to day 2 of spinning stage; P0-P6: Day 0 to day 6 of pupal stage. Click here for file

Additional file 4 **1D-PAGE (12%) electrophoretic profiles of protein collected from silkworm *B. mori *PVFB at larval, spinning and pupal stages (C, data for Figure **1C**) and Western blot of V instar 4^th ^day PVFB tissue sample using vitellogenin antibody (D)**. 30 kDa protein concentration increases during uptake and storage and decreases during late pupal stages and transformation to adult. M: Molecular weight marker; V1-V6: Day 1 to day 6 of V instar larval stage; S0-S2: Day 0 to day 2 of spinning stage; P0-P6: Day 0 to day 6 of pupal stage. Click here for file

Additional file 5 **Peptide data from the separation of 30 kDa lipoproteins from *B. mori *by 1D-PAGE and identification by LC-MS/MS or LC-MS^E^**. Click here for file

Additional file 6 **CLUSTAL format alignment by MAFFT (v6.811b) of LP1-LP5 and L301/L302 for visualization of detected tryptic peptides**. Click here for file

Additional file 7 **UniProt Blast results for LP1_BOMMO (June 16, 2011)**. Click here for file

Additional file 8 **UniProt Blast sequence alignment best matches for LP1_BOMMO (June 16, 2011)**. Click here for file

Additional file 9 **Mafft (v6.857b) alignment for C7A8A2 and Q17185**. C7A8A2 is identical to LP1 (P09334) except for the presence of N instead of K in position 114 (marked in blue) and was therefore removed from the considerations discussed in the main text. Click here for file

Additional file 10 **Mafft (v6.857b) alignment for C7A8A3 and D4QGB9**. Click here for file

Additional file 11 **Mafft (v6.857b) alignment for L301 (Q00802) and LP3 (P09336)**. Click here for file

Additional file 12 **Clustal format Mafft (v6.857b) alignment for lipoprotein Blast matches to LP1**. Click here for file

Additional file 13 **Mafft phylogenetic tree of Blast sequence alignment matches for LP1_BOMMO (June 16, 2011)**. Click here for file

Additional file 14 **CLUSTAL format alignment by MAFFT (v6.811b) of LP1-LP5 and L301/L302 for visualization of detected tryptic peptides**. Click here for file

Additional file 15 **UniProt entries for proteins assigned to spots excised from the 2D gel map of total proteins isolated from hemolymph of day 3 of V instar larvae of *B. mori *using LC-MS/MS and database search**. Click here for file

Additional file 16 **Separation of 30 kDa lipoproteins from *B. mori *by DEAE ion chromatography followed by 1D-PAGE (bands resulting from fraction 34, Figure **5**)**. Identification by LC-MS/MS. Tentative assignment of tryptic peptides to lipoproteins LP1-LP5, L301/L302. Click here for file

Additional file 17 **Separation of 30 kDa lipoproteins from *B. mori *by DEAE ion chromatography followed by gel filtration chromatography and 1D-PAGE (band resulting from fraction 60, Figure **5**)**. Identification by LC-MS/MS. Tentative assignment of tryptic peptides to lipoproteins LP1-LP5, L301/L302. Click here for file

Additional file 18 **Exemplary spectra for peptides derived from LP2, LP4 and LP5 found in the purified lipoprotein (fraction 60, Figure **5**)**. Click here for file

Additional file 19 **Amino acid analysis of purified protein**. Click here for file
