# Supplementary material for: A proteomic view on the developmental transfer of homologous 30 kDa lipoproteins from peripheral fat body to perivisceral fat body via hemolymph in silkworm, Bombyx mori
Source: BMC Biochem. 2012 Feb 28;13:5. doi: 10.1186/1471-2091-13-5 (PMC3306753; doi:10.1186/1471-2091-13-5)
Supplement: Additional file 1 — Database and sequence references for B. mori lipoproteins. [file 1471-2091-13-5-S1.PDF]

Additional file 1 – Database and sequence references for *B. mori* lipoproteins.

**UniProt#**		**Protein**	**Strain**	**Source**	**Reference**
P09334	LP1	Low molecular 30 kDa lipoprotein	Tokai X Asahi	Fat body	Structures and expression of mRNAs coding for major plasma proteins of Bombyx mori. Sakai N., Mori S., Izumi S., Haino-Fukushima K., Ogura T., Maekawa H., Tomino S. *Biochim. Biophys. Acta* 949:224-232 (1988)
P09335	LP2	Low molecular 30 kDa lipoprotein	Tokai X Asahi	Fat body	Structures and expression of mRNAs coding for major plasma proteins of Bombyx mori. Sakai N., Mori S., Izumi S., Haino-Fukushima K., Ogura T., Maekawa H., Tomino S. *Biochim. Biophys. Acta* 949:224-232 (1988)
P09336	LP3	Low molecular 30 kDa lipoprotein	Tokai X Asahi	Fat body	Structures and expression of mRNAs coding for major plasma proteins of Bombyx mori. Sakai N., Mori S., Izumi S., Haino-Fukushima K., Ogura T., Maekawa H., Tomino S. *Biochim. Biophys. Acta* 949:224-232 (1988)
P09337	LP4	Low molecular 30 kDa lipoprotein	Tokai X Asahi	Fat body	Structures and expression of mRNAs coding for major plasma proteins of Bombyx mori. Sakai N., Mori S., Izumi S., Haino-Fukushima K., Ogura T., Maekawa H., Tomino S. *Biochim. Biophys. Acta* 949:224-232 (1988)
P09338	LP5	Low molecular 30 kDa lipoprotein	Tokai X Asahi	Fat body	Structures and expression of mRNAs coding for major plasma proteins of Bombyx mori. Sakai N., Mori S., Izumi S., Haino-Fukushima K., Ogura T., Maekawa H., Tomino S. *Biochim. Biophys. Acta* 949:224-232 (1988)
Q00802	L301	Low molecular 30 kDa lipoprotein	Tokai X Asahi	Fat body	Complete nucleotide sequences of major plasma protein genes of Bombyx mori. Mori S., Izumi S., Tomino S. *Biochim. Biophys. Acta* 1090:129-132 (1991)
Q00801	L302	Low molecular 30 kDa lipoprotein	Tokai X Asahi	Fat body	Complete nucleotide sequences of major plasma protein genes of Bombyx mori. Mori S., Izumi S., Tomino S. *Biochim. Biophys. Acta* 1090:129-132 (1991)
Q17185		30 kDa protein	Tokai X	Fat body	Structures and organization of major plasma protein genes of the

			Asahi		silkworm Bombyx mori. Mori S., Izumi S., Tomino S. *J. Mol. Biol.* 218:7-12 (1991)
Q05432		Hemolymph protein	Tokai X Asahi		Gene structure of Bombyx mori larval serum protein (BmLSP). Fujiwara Y., Yamashita O. *Insect Mol. Biol.* 1:63-69 (1992)
C7A8A2		30 kDa protein	Bom m 9		Yang R. (2008) Shenzhen University, China
C7A8A3	19G1	Low molecular mass 30 kDa lipoprotein	Bom m 11		Yang R. (2008) Shenzhen University, China
D4QGC0	19G1P	Putative 30 kDa uncharacterized protein	C60		Isolation of a partial DNA. Ueno Y., Banno Y., Aso Y.
D4QGB9	19G1Q	Putative 30 kDa uncharacterized protein	C60		Isolation of a partial DNA. Ueno Y., Banno Y., Aso Y.
B5BSX5				Hemocyte	Purification and characterization of silkworm hemocytes by flow cytometry. Nakahara Y., Shimura S., Ueno C., Kanamori Y., Mita K., Kiuchi M., Kamimura M. *Dev. Comp. Immunol.* 33:439-448 (2009)
A7LIK7		30 kDa lipoprotein	Qingsong Haoyue		Cloning and bioinformatics analysis of a parthenogenesis-related differential 30K lipoprotein precursor gene in Bombyx mori. Wang D., Nie Z., Long X., Chen J., Lv Z., He P., Wu X., Zhang Y.
Q6Q0S8		Major plasma protein 30K (Fragment)	Zhejiang Chunxiao		Complete coding-region nucleotide sequence of a major plasma protein of Bombyx mori. Chen J., Zhu C.-G., Zhang Y.-Z.
Q75RW3		BmLSP-T	R06		Isolation and Characterization of Differently Expressed cDNAs in a Meiotic Recombination Strain of Bombyx mori. Miyagawa Y., Kusakabe T., Lee J., Maeda T., Kawaguchi Y., Koga K. *J. Insect Biotechnol. Sericology* 73:117-127 (2004)
Q2PQU4		Putative paralytic peptide-binding protein			Cloning and characterization of Bombyx mori PP-BP a gene induced by viral infection. Hu Z.G., Chen K.P., Yao Q., Gao G.T., Xu J.P., Chen H.Q. Yi *Chuan Xue Bao* 33:975-983 (2006)
E5EVW2		30kDa protein			Proteomic analysis of silkworm egg proteins during germ-band formation stage and the function analysis of BmEP80 protein. Xu Y., Fu Q., Xiang Z., He N.
E5EVW3		30kDa protein			Proteomic analysis of silkworm egg proteins during germ-band

					formation stage and the function analysis of BmEP80 protein.<br>Xu Y., Fu Q., Xiang Z., He N.
		30 kDa proteins Bmlp1-10	Dazao P50	Fat body	Analysis of the structure and expression of the 30 K protein genes in silkworm Bombyx mori. Sun, Q., Zhao, P., Lin, Y., Hou, Y., Xia, Q.-Y., Xiang, Z.-H. *Insect Sci.* 14: 5-14 (2007)
Q76IB6		Growth blocking peptide binding protein	*Pseudaletia separata*	Hemocyte	Insect cytokine growth-blocking peptide triggers a termination system of cellular immunity by inducing its binding protein.<br>Matsumoto Y., Oda Y., Uryu M., Hayakawa Y.<br>*J. Biol. Chem.* 278:38579-38585 (2003)
Q0VJU3		Microvitellogenin	*Manduca sexta*	Egg	Innate immunity: Eggs of Manduca sexta are able to respond to parasitism by Trichogramma evanescens. Abdel-latief M., Hilker M.
P19616		Microvitellogenin	*Manduca sexta*		The nucleotide sequence of a microvitellogenin encoding gene from the tobacco hornworm, Manduca sexta.<br>Wang X., Cole K.D., Law J.H. *Gene* 80:259-268 (1989)<br>cDNA cloning and deduced amino acid sequence of microvitellogenin, a female specific hemolymph and egg protein from the tobacco hornworm, Manduca sexta.<br>Wang X., Cole K.D., Law J.H. *J. Biol. Chem.* 263:8851-8855 (1988)

UniProt accession numbers, species information and reference for the discussed lipoprotein sequences as available for download to date (June 2011; Exception: Sun 2007).
