# Supplementary figures and images for: A proteomic view on the developmental transfer of homologous 30 kDa lipoproteins from peripheral fat body to perivisceral fat body via hemolymph in silkworm, Bombyx mori
Source: BMC Biochem. 2012 Feb 28;13:5. doi: 10.1186/1471-2091-13-5 (PMC3306753; doi:10.1186/1471-2091-13-5)

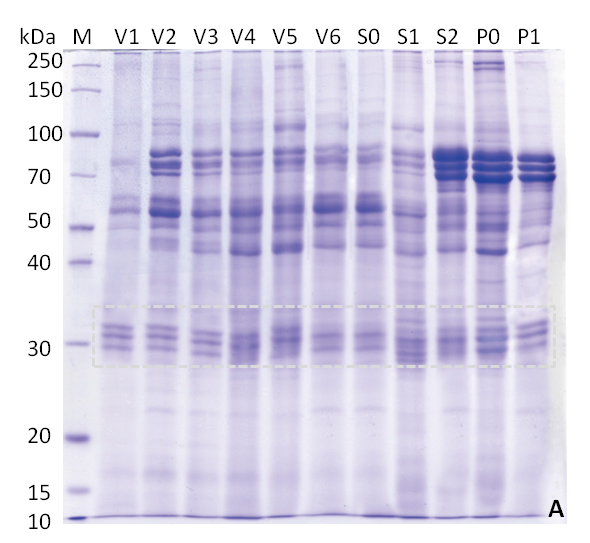

Supplement: Additional file 2 — 1D-PAGE (12%) electrophoretic profiles of protein collected from silkworm B. mori PPFB at larval and spinning stages. Data for Figure 1A. 30 kDa proteins are synthesized and secreted into hemolymph. M: Molecular weight marker; V1-V6: Day 1 to day 6 of V instar larval stage; S0-S2: Day 0 to day 2 of spinning stage; P0-P6: Day 0 to day 6 of pupal stage. [file 1471-2091-13-5-S2.TIFF]

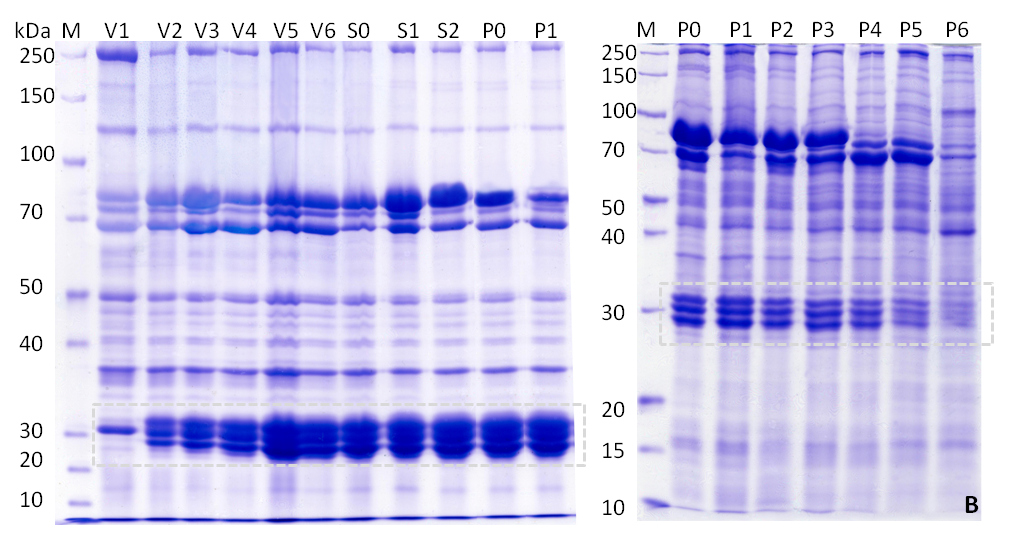

Supplement: Additional file 3 — 1D-PAGE (12%) electrophoretic profiles of protein collected from silkworm B. mori hemolymph at larval, spinning and pupal stages. Data for Figure 1B. 30 kDa protein concentration increases during synthesis and decreases with uptake into PVFB. M: Molecular weight marker; V1-V6: Day 1 to day 6 of V instar larval stage; S0-S2: Day 0 to day 2 of spinning stage; P0-P6: Day 0 to day 6 of pupal stage. [file 1471-2091-13-5-S3.TIFF]

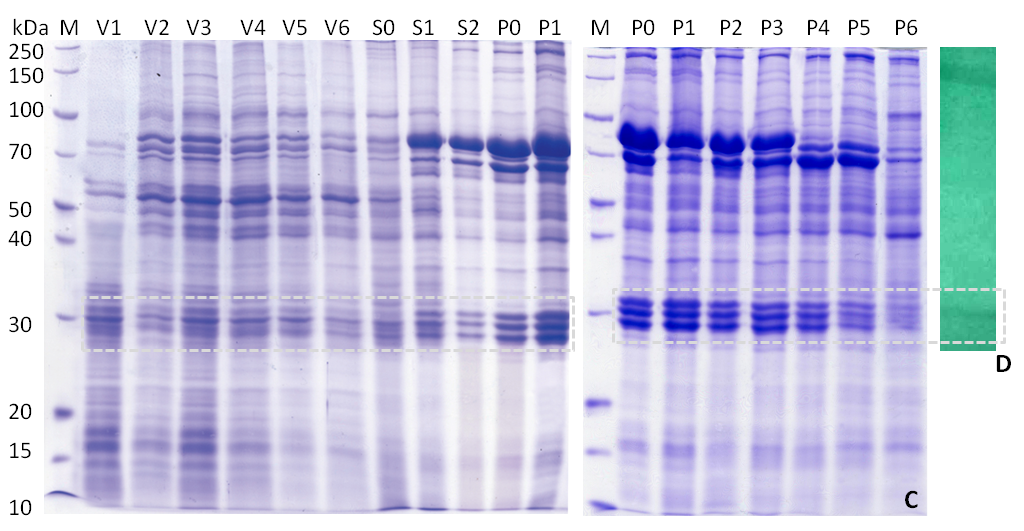

Supplement: Additional file 4 — 1D-PAGE (12%) electrophoretic profiles of protein collected from silkworm B. mori PVFB at larval, spinning and pupal stages (C, data for Figure 1C) and Western blot of V instar 4th day PVFB tissue sample using vitellogenin antibody (D). 30 kDa protein concentration increases during uptake and storage and decreases during late pupal stages and transformation to adult. M: Molecular weight marker; V1-V6: Day 1 to day 6 of V instar larval stage; S0-S2: Day 0 to day 2 of spinning stage; P0-P6: Day 0 to day 6 of pupal stage. [file 1471-2091-13-5-S4.TIFF]
