# Supplementary material for: A proteomic view on the developmental transfer of homologous 30 kDa lipoproteins from peripheral fat body to perivisceral fat body via hemolymph in silkworm, Bombyx mori
Source: BMC Biochem. 2012 Feb 28;13:5. doi: 10.1186/1471-2091-13-5 (PMC3306753; doi:10.1186/1471-2091-13-5)
Supplement: Additional file 5 — Peptide data from the separation of 30 kDa lipoproteins from B. mori by 1D-PAGE and identification by LC-MS/MS or LC-MSE. [file 1471-2091-13-5-S5.PDF]

**Additional file 5 - (Additional_file_5.pdf) Peptide data from the separation of 30 kDa lipoproteins from *B. mori* by 1D-PAGE and identification by LC-MS/MS or LC-MS$^E$.**

| Sequence | LP1 | LP2 | LP3 | LP4 | LP5 | L301 | L302 |
|---|---|---|---|---|---|---|---|
| **Day 0, peripheral, 29 kDa** | | | | | | | |
| LYNSILTGDYDSAVR | | x | | x | x | | x |
| LGPTLDPANER | | | | x | | | x |
| VIFGTNTADTTR | | | | | | | x |
| EQWFLQPTK | | | | x | | | x |
| VIFTEQTVK | x | | | | | | |
| FTPVLENNR | x | | | | | | |
| IIYGDSTADTFK | x | | | | | | |
| **Day 0, peripheral, 30 kDa** | | | | | | | |
| FITLWENNR | | x | | | x | | x |
| LYNSILTGDYDSAVR | | x | | x | x | | x |
| YENDVLFFIYNR | | x | | x | x | | x |
| YFPLSFR | | x | | | | | |
| EQWFFQPAK | | x | | | x | | |
| LIMAGNYVK | | x | | | x | | |
| QFNDALELGTIVNASGDR | | x | | | x | | |
| VVYGGNSADSTR | | x | | | x | | |
| KSEVITNVVNK | | | x | | | x | |
| LGPTLDPANER | | | | | | | x |
| LWVGNGQEIVR | | | | | x | | |
| SEVITNVVNK | | | x | | | x | |
| SLEYESQGQGSIVQNVVNNLIIDK | | x | | | | | |
| AQWYLQPAK | | | x | | | x | |
| MAWGYNGR | | | x | | | x | |
| **Day 0, peripheral, 31 kDa** | | | | | | | |
| FITLWENNR | | x | | | x | | x |
| LYNSILTGDYDSAVR | | x | | x | x | | x |
| EQWFFQPAK | | x | | | x | | |
| VVYGGNSADSTR | | x | | | x | | |
| KSEVITNVVNK | | | x | | | x | |
| LIALWENNK | | | x | | | x | |
| SEVITNVVNK | | | x | | | x | |
| SKHLYEEK | | | x | | | x | |
| YDNDVLFYIYNR | | | x | | | x | |
| AQWYLQPAK | | | x | | | x | |
| MAWGYNGR | | | x | | | x | |
| **Day 0, perivisceral, 29 kDa** | | | | | | | |
| FITLWENNR | | x | | | x | | x |
| LWVGNGQDIVK | | x | | | | | |
| LYNSILTGDYDSAVR | | x | | x | x | | x |
| YENDVLFFIYNR | | x | | x | x | | x |
| EQWFFQPAK | | x | | | x | | |
| LGPTLDPANER | | | | x | | | x |
| LWVGNGQEIVR | | | | | x | | |
| LWVGNGQHIVR | | | | x | | | x |
| LYNSILTGDYDSAVR | | x | | x | x | | x |
| YFPLNFR | | | | | x | | |

| | | | | | | | |
|---|---|---|---|---|---|---|---|
| EQWFLQPTK | | | | x | | | x |
| NYNLALK | | x | | x | x | | x |
| QSLEYENQGK | | | | x | | | x |
| **Day 0, perivisceral, 29 kDa** | | | | | | | |
| FTPVLENNR | x | | | | | | |
| SYFPIQFR | x | | | | | | |
| VSWK | x | | x | | | x | |
| IIYGDSTADTFK | x | | | | | | |
| NTMDFAYQLWTK | x | | | | | | |
| VIFTEQTVK | x | | | | | | |
| FITLWENNR | | x | | | x | | x |
| GSIIQNVVNNLIIDK | | | | | x | | |
| LGPTLDPANER | | | | x | | | x |
| LWVGNGQDIVK | | x | | | | | |
| LWVGNGQEIVR | | | | | x | | |
| LYNSILTGDYDSAVR | | x | | x | x | | x |
| SLEYESQGQGSIVQNVVNNLIIDK | | x | | | | | |
| YENDVLFFIYNR | | x | | x | x | | x |
| YFPLNFR | | | | | x | | |
| EQWFLQPTK | | | | x | | | x |
| KYFPLNFR | | | | | x | | |
| LIMAGNFVK | | | | x | | | x |
| NYNLALK | | x | | x | x | | x |
| QFNDALELGTIVNASGDR | | x | | | x | | |
| QSLEYESQGK | | | | | x | | |
| VIFGTNTADTTR | | | | | | | x |
| VVYGGNSADSTR | | x | | | x | | |
| **Day 0, perivisceral, 31 kDa** | | | | | | | |
| FITLWENNR | | x | | | x | | x |
| GSIIQNVVNNLIIDK | | | | | x | | |
| GSIIQNVVNNLIIDKR | | | | | x | | |
| KSEVITNVVNK | | | x | | | x | |
| LIALWENNK | | | x | | | x | |
| LWVGNGQEIVR | | | | | x | | |
| LYNSILTGDYDSAVR | | x | | x | x | | x |
| YDNDVLFYIYNR | | | x | | | x | |
| YFPLSFR | | x | | | | | |
| EQWFFQPAK | | x | | | x | | |
| MAWGYNGR | | | x | | | x | |
| VIGSPEHYAWGIK | | | x | | | x | |
| VVYGGNSADSTR | | x | | | x | | |
| **Day 1, peripheral, 29 kDa** | | | | | | | |
| GSIIQNVVNNLIIDK | | | | | x | | |
| LWVGNGQEIVR | | | | | x | | |
| LYNSILTGDYDSAVR | | x | | x | x | | x |
| EQWFFQPAK | | x | | | x | | |
| QSLEYESQGK | | | | | x | | |
| FITLWENNR | | x | | | x | | x |
| HTELVSWK | | | | | x | | |
| KSEVITNVVNK | | | x | | | x | |

| | | | | | | | |
|---|---|---|---|---|---|---|---|
| LGPTLDPANER | | | | x | | | x |
| LWVGNGQEIVR | | | | | x | | |
| LYNSILTGDYDSAVR | | x | | x | x | | x |
| AQWYLQPAK | | | x | | | x | |
| VIFGTNTADTTR | | | | | | | x |
| VVYGGNSADSTR | | x | | | x | | |
| **Day 1, peripheral, 31 kDa** | | | | | | | |
| FITLWENNR | | x | | | x | | x |
| LYNSILTGDYDSAVR | | x | | x | x | | x |
| EQWFFQPAK | | x | | | x | | |
| KSEVITNVVNK | | | x | | | x | |
| LIALWENNK | | | x | | | x | |
| LWVGNGQEIVR | | | | | x | | |
| YDNDVLFYIYNR | | | x | | | x | |
| AQWYLQPAK | | | x | | | x | |
| LIMAGNYVK | | | | | x | | |
| MAWGYNGR | | | x | | | x | |
| **Day 1, hemolymph, 26 kDa** | | | | | | | |
| FTPVLENNR | x | | | | | | |
| IMSTEDKQYLK | x | | | | | | |
| RNTMDFAYQLWTK | x | | | | | | |
| SYFPIQFR | x | | | | | | |
| **EALGHSGEVSGYPQLFAWYIVPY** | x | | | | | | |
| EYNSVMTLDEDMAANEDR | x | | | | | | |
| IIYGDSTADTFK | x | | | | | | |
| NTMDFAYQLWTK | x | | | | | | |
| TDDVLAEQLYMSVVIGEYETAIAK | x | | | | | | |
| VIFTEQTVK | x | | | | | | |
| LIMAGNFVK | | | | x | | | x |
| EKNSDLISWK | | | | x | | | x |
| FITLWENNR | | x | | | x | | x |
| GSIIQNVVNNLIIDGSR | | | | x | | | x |
| GSIIQNVVNNLIIDK | | | | | x | | |
| HTELVSWK | | | | | x | | |
| LGPTLDPANER | | | | x | | | x |
| LWVGNGQHIVR | | | | x | | | x |
| LYNSILTGDYDSAVR | | x | | x | x | | x |
| YENDVLFFIYNR | | x | | x | x | | x |
| YFPYNFR | | | | x | | | x |
| EQWFFQPAK | | | | | x | | |
| EQWFLQPTK | | | | x | | | x |
| KYFPYNFR | | | | x | | | x |
| LIMAGNFVK | | | | x | | | x |
| LIMAGNYVK | | x | | | x | | |
| NYNLALK | | x | | x | x | | x |
| QSLEYENQGK | | | | x | | | x |
| VIFGTNTADTTR | | | | | | | x |
| **Day 1, hemolymph, 30 kDa** | | | | | | | |
| FTPVLENNR | x | | | | | | |
| IMSTEDKQYLK | x | | | | | | |

| | | | | | | | |
|---|---|---|---|---|---|---|---|
| **EALGHSGEVSGYPQLFAWYIVPY** | x | | | | | | |
| EYNSVMTLDEDMAANEDR | x | | | | | | |
| IIYGDSTADTFK | x | | | | | | |
| NTMDFAYQLWTK | x | | | | | | |
| TDDVLAEQLYMSVVIGEYETAIAK | x | | | | | | |
| VIFTEQTVK | x | | | | | | |
| FITLWENNR | | x | | | x | | x |
| GSIIQNVVNNLIIDGSR | | | | x | | | x |
| GSIIQNVVNNLIIDK | | | | | x | | |
| GSIIQNVVNNLIIDKR | | | | | x | | |
| HTELVSWK | | | | | x | | |
| KSEVITNVVNK | | | x | | | x | |
| KYFPLSFR | | x | | | | | |
| LGPTLDPANER | | | | x | | | x |
| LGSTTNPSNER | | x | | | x | | |
| LIALWENNK | | | x | | | x | |
| LWVGNGQDIVK | | x | | | | | |
| LWVGNGQEIVR | | | | | x | | |
| LWVGNGQHIVR | | | | x | | | x |
| LYNSILTGDYDSAVR | | x | | x | x | | x |
| LYNSILTGDYDSAVRK | | x | | | | | |
| SEVITNVVNK | | | x | | | x | |
| SLEYESQGQGSIVQNVVNNLIIDK | | x | | | | | |
| SLEYESQGQGSIVQNVVNNLIIDKR | | x | | | | | |
| YDNDVLFYIYNR | | | x | | | x | |
| YENDVLFFIYNR | | x | | x | x | | x |
| YFPLNFR | | | | | x | | |
| YFPYNFR | | | | x | | | x |
| AQWYLQPAK | | | x | | | x | |
| DRVVYGGNSADSTR | | x | | | x | | |
| EQWFFQPAK | | x | | | x | | |
| EQWFLQPTK | | | | x | | | x |
| IAYGDGVDKHTELVSWK | | | | | x | | |
| KSLEYESQGQGSIVQNVVNNLIIDK | | x | | | | | |
| KYFPYNFR | | | | x | | | x |
| LIFAENAIK | | | x | | | x | |
| LIMAGNFVK | | | | x | | | x |
| MAWGYNGR | | | x | | | x | |
| QFNDALELGTIVNASGDR | | x | | | x | | |
| QFNDALELGTIVNASGDRK | | x | | | x | | |
| QSLEYENQGK | | | | x | | | x |
| QSLEYESQGK | | | | | x | | |
| VIFGTNTADTTR | | | | | | | x |
| VIGSPEHYAWGIK | | | x | | | x | |
| VVYGGNSADSTR | | x | | | x | | |
| **Day 1, hemolymph, 31 kDa** | | | | | | | |
| SYFPIQFR | x | | | | | | |
| EYNSVMTLDEDMAANEDR | x | | | | | | |
| NTMDFAYQLWTK | x | | | | | | |
| VIFTEQTVK | x | | | | | | |

| | | | | | | | |
|---|---|---|---|---|---|---|---|
| LIFAENAIK | | | x | | | x | |
| FITLWENNR | | x | | | x | | x |
| FTPVLENNR | x | | | | | | |
| GSIIQNVVNNLIIDGSR | | | | x | | | x |
| GSIIQNVVNNLIIDK | | | | | x | | |
| GSIIQNVVNNLIIDKR | | | | | x | | |
| HTELVSWK | | | | | x | | |
| KSEVITNVVNK | | | x | | | x | |
| LGPTLDPANER | | | | x | | | x |
| LWVGNGQDIVK | | x | | | | | |
| LWVGNGQEIVR | | | | | x | | |
| LYNSILTGDYDSAVR | | x | | x | x | | x |
| SLEYESQGQGSIVQNVVNNLIIDK | | x | | | | | |
| SLEYESQGQGSIVQNVVNNLIIDKR | | x | | | | | |
| YDNDVLFYIYNR | | | x | | | x | |
| YENDVLFFIYNR | | x | | x | x | | x |
| YFPLNFR | | | | | x | | |
| YFPLSFR | | x | | | | | |
| AQWYLQPAK | | | x | | | x | |
| EQWFFQPAK | | x | | | x | | |
| EYNSVMTLDEDMAANEDR | x | | | | | | |
| IAYGDGVDKHTELVSWK | | | | | x | | |
| IIYGDSTADTFK | x | | | | | | |
| KSLEYESQGQGSIVQNVVNNLIIDK | | x | | | | | |
| LIFAENAIK | | | x | | | x | |
| LIMAGNFVK | | | | x | | | x |
| LIMAGNYVK | | x | | | x | | |
| MAWGYNGR | | | x | | | x | |
| NYNLALK | | x | | x | x | | x |
| QFNDALELGTIVNASGDR | | x | | | x | | |
| QFNDALELGTIVNASGDRK | | x | | | x | | |
| QSLEYESQGK | | | | | x | | |
| VIFGTNTADTTR | | | | | | | x |
| VIGSPEHYAWGIK | | | x | | | x | |
| VVYGGNSADSTR | | x | | | x | | |
| **Day 1, perivisceral, 29 kDa** | | | | | | | |
| FTPVLENNR | x | | | | | | |
| IMSTEDKQYLK | x | | | | | | |
| SYFPIQFR | x | | | | | | |
| EYNSVMTLDEDMAANEDR | x | | | | | | |
| IIYGDSTADTFK | x | | | | | | |
| NTMDFAYQLWTK | x | | | | | | |
| VIFTEQTVK | x | | | | | | |
| FITLWENNR | | x | | | x | | x |
| GSIIQNVVNNLIIDGSR | | | | x | | | x |
| GSIIQNVVNNLIIDK | | | | | x | | |
| LGPTLDPANER | | | | x | | | x |
| LWVGNGQDIVK | | x | | | | | |
| LWVGNGQEIVR | | | | | x | | |
| LWVGNGQHIVR | | | | x | | | x |

| | | | | | | | |
|---|---|---|---|---|---|---|---|
| LYNSILTGDYDSAVR | | x | | x | x | | x |
| NSDLISWK | | | | x | | | x |
| YENDVLFFIYNR | | x | | x | x | | x |
| EQWFFQPAK | | x | | | x | | |
| EQWFLQPTK | | | | x | | | x |
| LIMAGNYVK | | x | | | x | | |
| QSLEYENQGK | | | | x | | | x |
| QSLEYESQGK | | | | | x | | |
| VIFGTNTADTTR | | | | | | | x |
| VVYGGNSADSTR | | x | | | x | | |
| **Day 1, perivisceral, 30 kDa** | | | | | | | |
| FTPVLENNR | x | | | | | | |
| SYFPIQFR | x | | | | | | |
| IIYGDSTADTFK | x | | | | | | |
| FITLWENNR | | x | | | x | | x |
| GSIIQNVVNNLIIDK | | | | | x | | |
| GSIIQNVVNNLIIDKR | | | | | x | | |
| KSEVITNVVNK | | | x | | | x | |
| LGPTLDPANER | | | | x | | | x |
| LWVGNGQDIVK | | x | | | | | |
| LWVGNGQEIVR | | | | | x | | |
| LYNSILTGDYDSAVR | | x | | x | x | | x |
| SEVITNVVNK | | | x | | | x | |
| SLEYESQGQGSIVQNVVNNLIIDK | | x | | | | | |
| SLEYESQGQGSIVQNVVNNLIIDKR | | x | | | | | |
| YDNDVLFYIYNR | | | x | | | x | |
| YENDVLFFIYNR | | x | | x | x | | x |
| YFPLNFR | | | | | x | | |
| DRVVYGGNSADSTR | | x | | | x | | |
| EQWFFQPAK | | x | | | x | | |
| IAYGDGVDKHTELVSWK | | | | | x | | |
| LIFAENAIK | | | x | | | x | |
| LIMAGNYVK | | x | | | x | | |
| MAWGYNGR | | | x | | | x | |
| NYNLALK | | x | | x | x | | x |
| QFNDALELGTIVNASGDR | | x | | | x | | |
| QSLEYESQGK | | | | | x | | |
| VIFGTNTADTTR | | | | | | | x |
| VVYGGNSADSTR | | x | | | x | | |
| **Day 1, perivisceral, 31 kDa** | | | | | | | |
| SYFPIQFR | x | | | | | | |
| IIYGDSTADTFK | x | | | | | | |
| VIFTEQTVK | x | | | | | | |
| LIALWENNK | | | x | | | x | |
| SEVITNVVNK | | | x | | | x | |
| YDNDVLFYIYNR | | | x | | | x | |
| MAWGYNGR | | | x | | | x | |
| FITLWENNR | | x | | | x | | x |
| LWVGNGQEIVR | | | | | x | | |
| LYNSILTGDYDSAVR | | x | | x | x | | x |

| | | | | | | | |
|---|---|---|---|---|---|---|---|
| QSLEYESQGK | | | | | x | | |
| VIFGTNTADTTR | | | | | | | x |
| VVYGGNSADSTR | | x | | | x | | |
| **Day 5, perivisceral, 29 kDa** | | | | | | | |
| FTPVLENNR | x | | | | | | |
| IMSTEDKQYLK | x | | | | | | |
| SYFPIQFR | x | | | | | | |
| **EALGHSGEVSGYPQLFAWYIVPY** | x | | | | | | |
| EYNSVMTLDEDMAANEDR | x | | | | | | |
| IIYGDSTADTFK | x | | | | | | |
| NTMDFAYQLWTK | x | | | | | | |
| VIFTEQTVK | x | | | | | | |
| FITLWENNR | | x | | | x | | x |
| GSIIQNVVNNLIIDGSR | | | | x | | | x |
| GSIIQNVVNNLIIDK | | | | | x | | |
| KSEVITNVVNK | | | x | | | x | |
| LGPTLDPANER | | | | x | | | x |
| LWVGNGQDIVK | | x | | | | | |
| LWVGNGQEIVR | | | | | x | | |
| LWVGNGQHIVR | | | | x | | | x |
| LYNSILTGDYDSAVR | | x | | x | x | | x |
| YENDVLFFIYNR | | x | | x | x | | x |
| YFPLSFR | | x | | | | | |
| YFPYNFR | | | | x | | | x |
| AQWYLQPAK | | | x | | | x | |
| EQWFFQPAK | x | | | | x | | |
| KYFPYNFR | | | | x | | | x |
| LIFAENAIK | | | x | | | x | |
| LIMAGNFVK | | | | x | | | x |
| MAWGYNGR | | | x | | | x | |
| NYNLALK | | x | | x | x | | x |
| QFNDALELGTIVNASGDR | | x | | | x | | |
| QSLEYENQGK | | | | x | | | x |
| QSLEYESQGK | | | | | x | | |
| SGIWTR | | | | x | | | |
| VIFGTNTADTTR | | | | | | | x |
| VVYGGNSADSTR | | x | | | x | | |
| **Day 5, perivisceral, 30 kDa** | | | | | | | |
| FTPVLENNR | x | | | | | | |
| SYFPIQFR | x | | | | | | |
| EYNSVMTLDEDMAANEDR | x | | | | | | |
| IIYGDSTADTFK | x | | | | | | |
| NTMDFAYQLWTK | x | | | | | | |
| VIFTEQTVK | x | | | | | | |
| LIFAENAIK | | | x | | | x | |
| AVGHDGEVAGLPDIYSWFITPF | | x | | | x | | |
| FITLWENNR | | x | | | x | | x |
| GSIIQNVVNNLIIDK | | | | | x | | |
| GSIIQNVVNNLIIDKR | | | | | x | | |
| HTELVSWK | | | | | x | | |

| | | | | | | | |
|---|---|---|---|---|---|---|---|
| KSEVITNVVNK | | | x | | | x | |
| KYFPLSFR | | x | | | | | |
| LGPTLDPANER | | | | x | | x | |
| LIALWENNK | | | x | | | x | |
| LWVGNGQDIVK | | x | | | | | |
| LWVGNGQEIVR | | | | | x | | |
| LYNSILTGDYDSAVR | | x | | x | x | x | x |
| NSDLISWK | | | | x | | | x |
| SLEYESQGQGSIVQNVVNNLIIDK | | x | | | | | |
| SLEYESQGQGSIVQNVVNNLIIDKR | | x | | | | | |
| YDNDVLFYIYNR | | | x | | | x | |
| YENDVLFFIYNR | | x | | x | x | | x |
| YFPLNFR | | | | | x | | |
| YFPLSFR | | x | | | | | |
| YNQYLK | | x | | x | x | | x |
| AQWYLQPAK | | | x | | | x | |
| EQWFFQPAK | | x | | | x | | |
| EQWFLQPTK | | | | x | | | x |
| IAYGDGVDK | | | | | x | | |
| IAYGDGVDKHTELVSWK | | | | | x | | |
| KSLEYESQGQGSIVQNVVNNLIIDK | | x | | | | | |
| KYFPLNFR | | | | | x | | |
| LIFAENAIK | | | x | | | x | |
| LIMAGNFVK | | | | x | | | x |
| MAWGYNGR | | | x | | | x | |
| NYNLALK | | x | | x | x | x | |
| QFNDALELGTIVNASGDR | | x | | | x | | |
| QFNDALELGTIVNASGDRK | | x | | | x | | |
| QSLEYESQGK | | | | | x | | |
| VIFGTNTADTTR | | | | | | | x |
| VIGSPEHYAWGIK | | | x | | | x | |
| VVYGGNSADSTR | | x | | | x | | |
| **Day 5, perivisceral, 31 kDa** | | | | | | | |
| FITLWENNR | | x | | | x | | x |
| LWVGNGQDIVK | | x | | | | | |
| LYNSILTGDYDSAVR | | x | | x | x | | x |
| SLEYESQGQGSIVQNVVNNLIIDK | | x | | | | | |
| SLEYESQGQGSIVQNVVNNLIIDKR | | x | | | | | |
| YENDVLFFIYNR | | x | | x | x | | x |
| EQWFFQPAK | | x | | | x | | |
| NYNLALK | | x | | x | x | | x |
| QFNDALELGTIVNASGDR | | x | | | x | | |
| VVYGGNSADSTR | | x | | | x | | |
| GSPEHYAWGIK | | | x | | | | x |
| GSIIQNVVNNLIIDK | | | | | x | | |
| GSIIQNVVNNLIIDKR | | | | | x | | |
| HTELVSWK | | | | | x | | |
| KSEVITNVVNK | | | x | | | x | |
| LGPTLDPANER | | | | x | | | x |
| LIALWENNK | | | x | | | x | |

| | | | | | | | |
|---|---|---|---|---|---|---|---|
| LWVGNGQEIVR | | | | | x | | |
| LYNSILTGDYDSAVR | | x | | x | x | | x |
| SEVITNVVNK | | | x | | | x | |
| YDNDVLFYIYNR | | | x | | | x | |
| AQWYLQPAK | | | x | | | x | |
| IAYGDGVDKHTELVSWK | | | | | x | | |
| MAWGYNGR | | | x | | | x | |
| QFNDALELGTIVNASGDRK | | x | | | x | | |
| QSLEYESQGK | | | | | x | | |
| VIGSPEHYAWGIK | | | x | | | x | |
| **Day 5, hemolymph, 29 kDa** | | | | | | | |
| FTPVLENNR | x | | | | | | |
| IMSTEDKQYLK | x | | | | | | |
| SYFPIQFR | x | | | | | | |
| EYNSVMTLDEDMAANEDR | x | | | | | | |
| IIYGDSTADTFK | x | | | | | | |
| NTMDFAYQLWTK | x | | | | | | |
| VIFTEQTVK | x | | | | | | |
| EKNSDLISWK | | | | x | | | x |
| FITLWENNR | | x | | | x | | x |
| GSIIQNVVNNLIIDGSR | | | | x | | | x |
| GSIIQNVVNNLIIDK | | | | | x | | |
| HTELVSWK | | | | | x | | |
| KSEVITNVVNK | | | x | | | x | |
| LGPTLDPANER | | | | x | | | x |
| LWVGNGQDIVK | | x | | | | | |
| LWVGNGQEIVR | | | | | x | | |
| LWVGNGQHIVR | | | | x | | | x |
| LYNSILTGDYDSAVR | | x | | x | x | | x |
| NSDLISWK | | | | x | | | x |
| SLEYESQGQGSIVQNVVNNLIIDK | | x | | | | | |
| SLEYESQGQGSIVQNVVNNLIIDKR | | x | | | | | |
| YENDVLFFIYNR | | x | | x | x | | x |
| YFPYNFR | | | | x | | | x |
| ACVTPACAEMSAVSMSSSNK | | | | | x | | |
| EQWFFQPAK | | x | | | x | | |
| EQWFLQPTK | | | | x | | | x |
| IAYGDGVDKHTELVSWK | | | | | x | | |
| KSLEYESQGQGSIVQNVVNNLIIDK | | x | | | | | |
| KYFPYNFR | | | | x | | | x |
| LIMAGNFVK | | | | x | | | x |
| NYNLALK | | x | | x | x | | x |
| QFNDALELGTIVNASGDR | | x | | | x | | |
| QFNDALELGTIVNASGDRK | | x | | | x | | |
| VIFGTNTADTTR | | | | | | | x |
| VVYGGNSADSTR | | x | | | x | | |
| **Day 5, hemolymph, 30 kDa** | | | | | | | |
| FTEQTVK | x | | | | | | |
| FTPVLENNR | x | | | | | | |
| EYNSVMTLDEDMAANEDR | x | | | | | | |

| | | | | | | | |
|---|---|---|---|---|---|---|---|
| IIYGDSTADTFK | x | | | | | | |
| NTMDFAYQLWTK | x | | | | | | |
| VIFTEQTVK | x | | | | | | |
| LIFAENAIK | | | x | | | x | |
| FITLWENNR | | x | | | x | | x |
| GSIIQNVVNNLIIDGSR | | | | x | | | x |
| GSIIQNVVNNLIIDK | | | | | x | | |
| GSIIQNVVNNLIIDKR | | | | | x | | |
| KSEVITNVVNK | | | x | | | x | |
| KYFPLSFR | | x | | | | | |
| LGPTLDPANER | | | | x | | | x |
| LIALWENNK | | | x | | | x | |
| LWVGNGQDIVK | | x | | | | | |
| LWVGNGQEIVR | | | | | x | | |
| LYNSILTGDYDSAVR | | x | | x | x | | x |
| NSDLISWK | | | | x | | | x |
| SEVITNVVNK | | | x | | | x | |
| SLEYESQGQGSIVQNVVNNLIIDK | | x | | | | | |
| SLEYESQGQGSIVQNVVNNLIIDKR | | x | | | | | |
| YDNDVLFYIYNR | | | x | | | x | |
| YENDVLFFIYNR | | x | | x | x | | x |
| YFPLNFR | | | | | x | | |
| YFPLSFR | | x | | | | | |
| AQWYLQPAK | | | x | | | x | |
| EQWFFQPAK | | x | | | x | | |
| EQWFLQPTK | | | | x | | | x |
| IAYGDGVDKHTELVSWK | | | | | x | | |
| KSLEYESQGQGSIVQNVVNNLIIDK | | x | | | | | |
| KYFPLNFR | | | | | x | | |
| LIFAENAIK | | | x | | | x | |
| LIMAGNFVK | | | | x | | | x |
| LIMAGNYVK | | x | | | x | | |
| MAWGYNGR | | | x | | | x | |
| NYNLALK | | x | | x | x | | x |
| QFNDALELGTIVNASGDR | | x | | | x | | |
| QSLEYESQGK | | | | | x | | |
| VIFGTNTADTTR | | | | | | | x |
| VIGSPEHYAWGIK | | | x | | | x | |
| VVYGGNSADSTR | | x | | | x | | |
| **Day 5, hemolymph, 31 kDa** | | | | | | | |
| LIFAENAIK | | | x | | | x | |
| FITLWENNR | | x | | | x | | x |
| GSIIQNVVNNLIIDK | | | | | x | | |
| GSIIQNVVNNLIIDKR | | | | | x | | |
| KSEVITNVVNK | | | x | | | x | |
| LGSTTNPSNER | | x | | | x | | |
| LWVGNGQDIVK | | x | | | | | |
| LWVGNGQEIVR | | | | | x | | |
| LYNSILTGDYDSAVR | | x | | x | x | | x |
| SEVITNVVNK | | | x | | | x | |

| | | | | | | | |
|---|---|---|---|---|---|---|---|
| YDNDVLFYIYNR | | | x | | | x | |
| YENDVLFFIYNR | | x | | x | x | | x |
| YFPLNFR | | | | | x | | |
| YFPLSFR | | x | | | | | |
| AQWYLQPAK | | | x | | | x | |
| EQWFFQPAK | | x | | | x | | |
| LIFAENAIK | | | x | | | x | |
| MAWGYNGR | | | x | | | x | |
| QFNDALELGTIVNASGDR | | x | | | x | | |
| QFNDALELGTIVNASGDRK | | x | | | x | | |
| QSLEYESQGK | | | | | x | | |
| VIGSPEHYAWGIK | | | x | | | x | |
| VVYGGNSADSTR | | x | | | x | | |

Tentative assignment of tryptic peptides to lipoproteins LP1-LP5, L301/L302. Peptides were often found to be deamidated or oxidized. Sequences marked in bold represent terminal peptides. Peptide location with the protein sequences is visualized in Additional file 6.
