# Supplementary material for: A proteomic view on the developmental transfer of homologous 30 kDa lipoproteins from peripheral fat body to perivisceral fat body via hemolymph in silkworm, Bombyx mori
Source: BMC Biochem. 2012 Feb 28;13:5. doi: 10.1186/1471-2091-13-5 (PMC3306753; doi:10.1186/1471-2091-13-5)
Supplement: Additional file 6 — CLUSTAL format alignment by MAFFT (v6.811b) of LP1-LP5 and L301/L302 for visualization of detected tryptic peptides. [file 1471-2091-13-5-S6.PDF]

Additional file 6 - CLUSTAL format alignment by MAFFT (v6.811b) of LP1-LP5 and L301/L302 for visualization of detected tryptic peptides.

```
sp|P09338|LP5_B MK---FLVVFAVVRACVTPACAEMSAVSMSSSNKELEEKLYNSILTGDYDSAVRQSLEYE
sp|P09335|LP2_B MK---LLVVFAMCVPAASAGVVELSADSMSPSNQDLEDKLYNSILTGDYDSAVRKSLEYE
sp|Q00801|L302_ MK---FLVVFASCVLAVSAGVAEMSAVSMSSSNKELEEKLYNSILTGDYDSAVRQSLEYE
sp|P09337|LP4_B MK---FVVVFASCVLAVSAGVTEMSAASMSSSNKELEEKLYNSILTGDYDSAVRQSLEYE
sp|P09336|LP3_B MKPAIVILCL---------FVASLYAADSDVPNDILEEQLYNSVVVADYDSAVEKSKHLY
sp|Q00802|L301_ MKPAIVILCL---------FVASLYAADSDVPNDILEEQLYNSVVVADYDSAVEKSKHLY
sp|P09334|LP1_B MRLTLFAFVLAVCALASNATLA-------PRTDDVLAEQLYMSVVIGEYETAIAKCSEYL
                *:   . . :           .         .:. * ::** *:: .:*::*: :. .

sp|P09338|LP5_B SQGKGSIIQNVVNNLIIDKRRNTMEYCYKLWVGNGQEIVRKYFPLNFRLIMAGNYVKIIY
sp|P09335|LP2_B SQGQGSIVQNVVNNLIIDKRRNTMEYCYKLWVGNGQDIVKKYFPLSFRLIMAGNYVKLIY
sp|Q00801|L302_ NQGKGSIIQNVVNNLIIDGSRNTMEYCYKLWVGNGQHIVRKYFPYNFRLIMAGNFVKLIY
sp|P09337|LP4_B NQGKGSIIQNVVNNLIIDGSRNTMEYCYKLWVGNGQHIVRKYFPYNFRLIMAGNFVKLIY
sp|P09336|LP3_B EEKKSEVITNVVNKLIRNNKMNCMEYAYQLWLQGSKDIVRDCFPVEFRLIFAENAIKLMY
sp|Q00802|L301_ EEKKSEVITNVVNKLIRNNKMNCMEYAYQLWLQGSKDIVRDCFPVEFRLIFAENAIKLMY
sp|P09334|LP1_B KEKKGEVIKEAVKRLIENGKRNTMDFAYQLWTKDGKEIVKSYFPIQFRVIFTEQTVKLIN
                .: :..:: :.*:.** :   * *::.*:**  ..:.**:. ** .**:*:: : :*::

sp|P09338|LP5_B RNYNLALKLGSTTNPSNERIAYGDGVDKHTELVSWKFITL-WENNRVYFKIHNTKYNQYL
sp|P09335|LP2_B RNYNLALKLGSTTNPSNERIAYGDGVDKHTDLVSWKFITL-WENNRVYFKAHNTKYNQYL
sp|Q00801|L302_ RNYNLALKLGPTLDPANERLAYGDGKEKNSDLISWKFITL-WENNRVYFKIHNTKYNQYL
sp|P09337|LP4_B RNYNLALKLGPTLDPANERLAYGDGKEKNSDLISWKSHYLVGEQHSVLQDPPTLSYNQYL
sp|P09336|LP3_B KRDGLALTLSNDVQGDDGRPAY--GKDKTSPRVSWKLIAL-WENNKVYFKILNTERNQYL
sp|Q00802|L301_ KRDGLALTLSNDVQGDDGRPRYGDGKDKTSPRVSWKLIAL-WENNKVYFKILNTERNQYL
sp|P09334|LP1_B KRDHHALKLID--QQNHNKIAFGDSKDKTSKKVSWKFTPV-LENNRVYFKIMSTEDKQYL
                :.   **.*    :  . :  :  . :* :  :***   :  *:: *  .  . . :***

sp|P09338|LP5_B KMSTTTCNCNSRDRVVYGGNSADSTREQWFFQPAKYENDVLFFIYNRQFND-ALELGTIV
sp|P09335|LP2_B KMSTSTCNCNARDRVVYGGNSADSTREQWFFQPAKYENDVLFFIYNRQFND-ALELGTIV
sp|Q00801|L302_ KLSSTT-DCNTQDRVIFGTNTADTTREQWFLQPTKYENDVLFFIYNREYND-ALKLGRIV
sp|P09337|LP4_B KLSSTT-DCNTQDRIIFGTNTADTTREQWFLQPTKYENDVLFFIYNREVQRVALKLGRIV
sp|P09336|LP3_B VLGVGT-NWNG-DHMAFGVNSVDSFRAQWYLQPAKYDNDVLFYIYNREYSK-ALTLSRTV
sp|Q00802|L301_ VLGVGT-NWNG-DHMAFGVNSVDSFRAQWYLQPAKYDNDVLFYIYNREYSK-ALTLSRTV
sp|P09334|LP1_B KLDNTK--GSSDDRIIYGDSTADTFKHHWYLEPSMYESDVMFFVYNREYNS-VMTLDEDM
                 :.  .   .  *:: :*  .:.*: : :*:::*: *:.**:*::***: .  .: *.  :

sp|P09338|LP5_B NASGDRKAVGHDGEVAGLPDIYSWFITPF
sp|P09335|LP2_B NASGDRKAVGHDGEVAGLPDIYSWFITPF
sp|Q00801|L302_ DASGDRMAFGHDGEVAGLPDIFSWFVTPF
sp|P09337|LP4_B DASGDRSGI--------------WTRWMK
sp|P09336|LP3_B EPSGHRMAWGYNGRVIGSPEHYAWGIKAF
sp|Q00802|L301_ EPSGHRMAWGYNGRVIGSPEHYAWGIKAF
sp|P09334|LP1_B AANEDREALGHSGEVSGYPQLFAWYIVPY
                 .. .* .               *
```
