# Supplementary material for: A proteomic view on the developmental transfer of homologous 30 kDa lipoproteins from peripheral fat body to perivisceral fat body via hemolymph in silkworm, Bombyx mori
Source: BMC Biochem. 2012 Feb 28;13:5. doi: 10.1186/1471-2091-13-5 (PMC3306753; doi:10.1186/1471-2091-13-5)
Supplement: Additional file 7 — UniProt Blast results for LP1_BOMMO (June 16, 2011). [file 1471-2091-13-5-S7.PDF]

Additional file 7 - (Additional_file_7.pdf) UniProt Blast results for LP1_BOMMO (June 16, 2011).

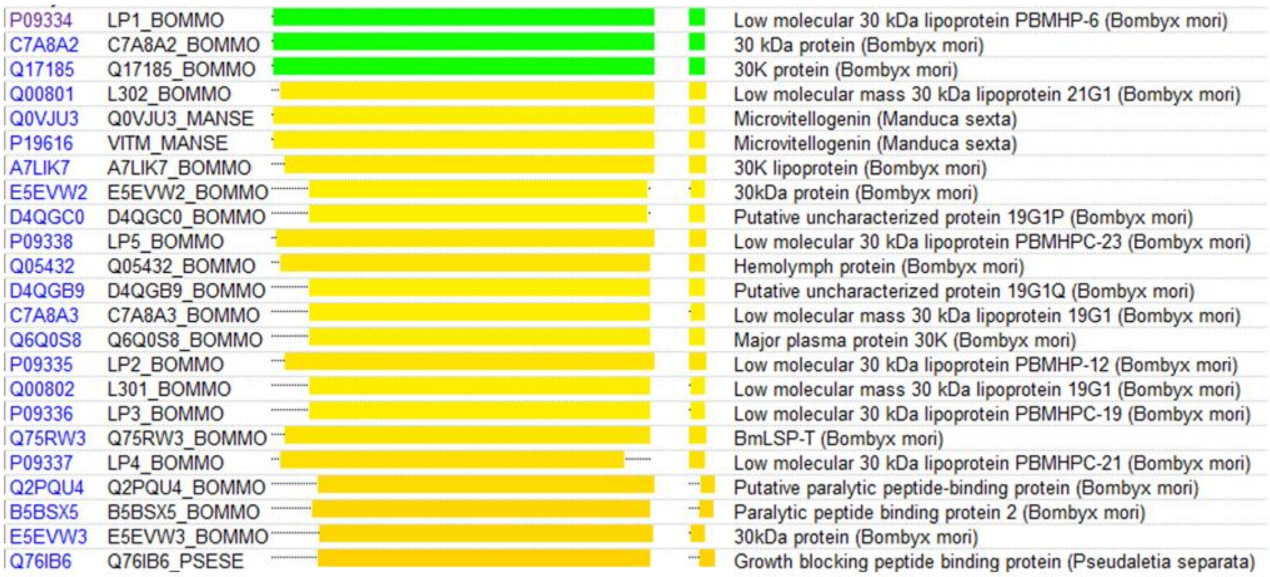

P09334	LP1_BOMMO	Low molecular 30 kDa lipoprotein PBMHP-6 (Bombyx mori)
C7A8A2	C7A8A2_BOMMO	30 kDa protein (Bombyx mori)
Q17185	Q17185_BOMMO	30K protein (Bombyx mori)
Q00801	L302_BOMMO	Low molecular mass 30 kDa lipoprotein 21G1 (Bombyx mori)
Q0VJU3	Q0VJU3_MANSE	Microvitellogenin (Manduca sexta)
P19616	VITM_MANSE	Microvitellogenin (Manduca sexta)
A7LIK7	A7LIK7_BOMMO	30K lipoprotein (Bombyx mori)
E5EVW2	E5EVW2_BOMMO	30kDa protein (Bombyx mori)
D4QGC0	D4QGC0_BOMMO	Putative uncharacterized protein 19G1P (Bombyx mori)
P09338	LP5_BOMMO	Low molecular 30 kDa lipoprotein PBMHPC-23 (Bombyx mori)
Q05432	Q05432_BOMMO	Hemolymph protein (Bombyx mori)
D4QGB9	D4QGB9_BOMMO	Putative uncharacterized protein 19G1Q (Bombyx mori)
C7A8A3	C7A8A3_BOMMO	Low molecular mass 30 kDa lipoprotein 19G1 (Bombyx mori)
Q6Q0S8	Q6Q0S8_BOMMO	Major plasma protein 30K (Bombyx mori)
P09335	LP2_BOMMO	Low molecular 30 kDa lipoprotein PBMHP-12 (Bombyx mori)
Q00802	L301_BOMMO	Low molecular mass 30 kDa lipoprotein 19G1 (Bombyx mori)
P09336	LP3_BOMMO	Low molecular 30 kDa lipoprotein PBMHPC-19 (Bombyx mori)
Q75RW3	Q75RW3_BOMMO	BmLSP-T (Bombyx mori)
P09337	LP4_BOMMO	Low molecular 30 kDa lipoprotein PBMHPC-21 (Bombyx mori)
Q2PQU4	Q2PQU4_BOMMO	Putative paralytic peptide-binding protein (Bombyx mori)
B5BSX5	B5BSX5_BOMMO	Paralytic peptide binding protein 2 (Bombyx mori)
E5EVW3	E5EVW3_BOMMO	30kDa protein (Bombyx mori)
Q76IB6	Q76IB6_PSESE	Growth blocking peptide binding protein (Pseudaletia separata)
