# Supplementary material for: A proteomic view on the developmental transfer of homologous 30 kDa lipoproteins from peripheral fat body to perivisceral fat body via hemolymph in silkworm, Bombyx mori
Source: BMC Biochem. 2012 Feb 28;13:5. doi: 10.1186/1471-2091-13-5 (PMC3306753; doi:10.1186/1471-2091-13-5)
Supplement: Additional file 8 — UniProt Blast sequence alignment best matches for LP1_BOMMO (June 16, 2011). [file 1471-2091-13-5-S8.PDF]

Additional file 8 - UniProt Blast sequence alignment best matches for LP1_BOMMO (June 16, 2011). Hydrophobic amino acids are shown in blue.

```
1    ------------------------------------------------------------    0  P09334  LP1_BOMMO
1    ------------------------------------------------------------    0  C7A8A2  C7A8A2_BOMMO
1    ------------------------------------------------------------    0  Q17185  Q17185_BOMMO
1    ------------------------------------------------------------    0  Q75RW3  Q75RW3_BOMMO
1    ------------------------------------------------------------    0  Q05432  Q05432_BOMMO
1    ------------------------------------------------------------    0  Q0VJU3  Q0VJU3_MANSE
1    ------------------------------------------------------------    0  P19616  VITM_MANSE
1    ------------------------------------------------------------    0  P09336  LP3_BOMMO
1    ------------------------------------------------------------    0  Q00802  L301_BOMMO
1    ------------------------------------------------------------    0  C7A8A3  C7A8A3_BOMMO
1    ------------------------------------------------------------    0  D4QGB9  D4QGB9_BOMMO
1    ------------------------------------------------------------    0  Q6Q0S8  Q6Q0S8_BOMMO
1    ------------------------------------------------------------    0  D4QGC0  D4QGC0_BOMMO
1    ------------------------------------------------------------    0  E5EVW2  E5EVW2_BOMMO
1    ------------------------------------------------------------    0  P09335  LP2_BOMMO
1    ------------------------------------------------------------    0  A7LIK7  A7LIK7_BOMMO
1    ------------------------------------------------------------    0  P09338  LP5_BOMMO
1    ------------------------------------------------------------    0  P09337  LP4_BOMMO
1    ------------------------------------------------------------    0  Q00801  L302_BOMMO
1    ------------------------------------------------------------    0  E5EVW3  E5EVW3_BOMMO
1    ---------------MMDPQDKLPNYVTNVDLKYPYSDLPYIGQYKLLKLPFTG-KLIEH   44  B5BSX5  B5BSX5_BOMMO
1    -----------MRSLTIWKLAQRPNFNTNINKQFPYSETPYQGDYYLEKIPISLNNLIQH   49  Q76IB6  Q76IB6_PSESE
1    MTTSFSHSQVKTPSEEKWEAASEPDYHTNEDLLYPYSPIPYFGMYHLVKIPIGR-GLVHH   59  Q2PQU4  Q2PQU4_BOMMO

1    ------------------------------------------------------------    0  P09334  LP1_BOMMO
1    ------------------------------------------------------------    0  C7A8A2  C7A8A2_BOMMO
1    ------------------------------------------------------------    0  Q17185  Q17185_BOMMO
1    ------------------------------------------------------------    0  Q75RW3  Q75RW3_BOMMO
1    ------------------------------------------------------------    0  Q05432  Q05432_BOMMO
1    ------------------------------------------------------------    0  Q0VJU3  Q0VJU3_MANSE
1    ------------------------------------------------------------    0  P19616  VITM_MANSE
1    ------------------------------------------------------------    0  P09336  LP3_BOMMO
1    ------------------------------------------------------------    0  Q00802  L301_BOMMO
1    ------------------------------------------------------------    0  C7A8A3  C7A8A3_BOMMO
1    ------------------------------------------------------------    0  D4QGB9  D4QGB9_BOMMO
1    ------------------------------------------------------------    0  Q6Q0S8  Q6Q0S8_BOMMO
1    ------------------------------------------------------------    0  D4QGC0  D4QGC0_BOMMO
1    ------------------------------------------------------------    0  E5EVW2  E5EVW2_BOMMO
1    ------------------------------------------------------------    0  P09335  LP2_BOMMO
1    ------------------------------------------------------------    0  A7LIK7  A7LIK7_BOMMO
1    ------------------------------------------------------------    0  P09338  LP5_BOMMO
1    ------------------------------------------------------------    0  P09337  LP4_BOMMO
1    ------------------------------------------------------------    0  Q00801  L302_BOMMO
1    ------------------------------------------------------------    0  E5EVW3  E5EVW3_BOMMO
45   VDYWGEGSIVNGGLYSGFRNCYNVNRQYQEVSNGPDMGRKIPNRIPVRDENDCDTRAYIK  104  B5BSX5  B5BSX5_BOMMO
50   VDYWGEGKVVTEEGVRGFSNCYNVNHQYQLVSSGPDKDRKIPNRIPVRSDEDCDTSSYIK  109  Q76IB6  Q76IB6_PSESE
60   VDYWGEGKVTNLDRVRGFRRSYNVNEQFALVSKGHSKGKQIPNRIPVVSVDDSDTSSYIR  119  Q2PQU4  Q2PQU4_BOMMO
```

```
1     ------------------------------------------------------MRLTLFAFV    9  P09334  LP1_BOMMO
1     ---------------------------------------------------MRLTLFAFV    9  C7A8A2  C7A8A2_BOMMO
1     ---------------------------------------------------MRLTLFAFV    9  Q17185  Q17185_BOMMO
1     ------------------------------------------------------MKTLAV    6  Q75RW3  Q75RW3_BOMMO
1     ------------------------------------------------------MKFLVF    6  Q05432  Q05432_BOMMO
1     ------------------------------------------------------MLRTTV    6  Q0VJU3  Q0VJU3_MANSE
1     ------------------------------------------------------MLRTTV    6  P19616  VITM_MANSE
1     ------------------------------------------------------MKPAIV    6  P09336  LP3_BOMMO
1     ------------------------------------------------------MKPAIV    6  Q00802  L301_BOMMO
1     ------------------------------------------------------MKPAIV    6  C7A8A3  C7A8A3_BOMMO
1     ------------------------------------------------------------    0  D4QGB9  D4QGB9_BOMMO
1     ------------------------------------------------------------    0  Q6Q0S8  Q6Q0S8_BOMMO
1     ------------------------------------------------------------    0  D4QGC0  D4QGC0_BOMMO
1     ------------------------------------------------------MKPVIV    6  E5EVW2  E5EVW2_BOMMO
1     ------------------------------------------------------MKLLVV    6  P09335  LP2_BOMMO
1     ------------------------------------------------------MKLLVV    6  A7LIK7  A7LIK7_BOMMO
1     ------------------------------------------------------MKFLVV    6  P09338  LP5_BOMMO
1     ------------------------------------------------------MKFVVV    6  P09337  LP4_BOMMO
1     ------------------------------------------------------MKFLVV    6  Q00801  L302_BOMMO
1     ---------------------------------------------------------MSV    3  E5EVW3  E5EVW3_BOMMO
105   DDSVKIVTLMSAP-IIPNSARDITRIVNERVGMVVIYGMPVESQGIKLLAAELKSKLLLY  163  B5BSX5  B5BSX5_BOMMO
110   DNSVLTVTVAEASRITSSCAKDIARIINSDHGKVIVYGVQGNSQEISELAVELRKKGLTP  169  Q76IB6  Q76IB6_PSESE
120   DGGVKTVTISTGP-ISKRCAADVARIVNASEGLVVAYGYSENSDDIQNLERELGKKGLYY  178  Q2PQU4  Q2PQU4_BOMMO

10    LAVCALASNAT--------LAPRTDDVLAEQLYMSVVIGEYETAIAKCSEYLKEKKGE-V   60  P09334  LP1_BOMMO
10    LAVCALASNAT--------LAPRTDDVLAEQLYMSVVIGEYETAIAKCSEYLKEKKGE-V   60  C7A8A2  C7A8A2_BOMMO
10    LAVCALASNAT--------LAPRTDDVLAEQLYMSVVIGEYETAIAKCSEYLKEKKGE-V   60  Q17185  Q17185_BOMMO
7     LALCLVAASATPSIDGDDRYPIHAPSGYEDIVTNAIITRNYEAAASMTVQLKRRSSGR-Y   65  Q75RW3  Q75RW3_BOMMO
7     FSTCVLAASAG-LIDLD-INILSAPTRAETRLVDAITTADYNTAVSLILLLEKQSSGS-I   63  Q05432  Q05432_BOMMO
7     VLLTLAAIAFA------------APT--SDDIYNNVVIGDIDGAVAKSKELQKQGKGD-I   51  Q0VJU3  Q0VJU3_MANSE
7     VLLTLAAIAFA------------APT--SDDIYNNVVIGDIDGAVAKSKELQKQGKGD-I   51  P19616  VITM_MANSE
7     ILCLFVAS-------LYAADSDVPNDILEEQLYNSVVVADYDSAVEKSKHLYEEKKSE-V   58  P09336  LP3_BOMMO
7     ILCLFVAS-------LYAADSDVPNDILEEQLYNSVVVADYDSAVEKSKHLYEEKKSE-V   58  Q00802  L301_BOMMO
7     ILCLFVAS-------LYAADSDVPNDILEEQLYNSVVVADYDSAVEKSKHLYEEKKSE-V   58  C7A8A3  C7A8A3_BOMMO
1     --------------------SDVPNDILEEQLYNSVVVADYDSAVEKSKHLYEEKKSE-V   39  D4QGB9  D4QGB9_BOMMO
1     ------------------ADSDVPNDILEEQLYNSVVVADYDSAVEKSKHLYGEKKSE-V   41  Q6Q0S8  Q6Q0S8_BOMMO
1     --------------------SDVPNDILEEQLYNSIVVADYDSAVEKSKHLYEEKKSE-V   39  D4QGC0  D4QGC0_BOMMO
7     ILCLFVAS-------LYAADSDVPNDILEEQLYNSIVVADYDSAVEKSKHLYEEKKSE-V   58  E5EVW2  E5EVW2_BOMMO
7     FAMCVPAASAG-VVELSADSMSPSNQDLEDKLYNSILTGDYDSAVRKSLEYESQGQGS-I   64  P09335  LP2_BOMMO
7     FAMCMLAASAG-VVELSAD---TSNQDLEEKLYNSILTGDYDSAVRQSLEYESQGKGS-I   61  A7LIK7  A7LIK7_BOMMO
7     FAVVRACVTPA-CAEMSAVSMSSSNKELEEKLYNSILTGDYDSAVRQSLEYESQGKGS-I   64  P09338  LP5_BOMMO
7     FASCVLAVSAG-VTEMSAASMSSSNKELEEKLYNSILTGDYDSAVRQSLEYENQGKGS-I   64  P09337  LP4_BOMMO
7     FASCVLAVSAG-VAEMSAVSMSSSNKELEEKLYNSILTGDYDSAVRQSLEYENQGKGS-I   64  Q00801  L302_BOMMO
4     EASDSG--------------------KSDRKLYNSVITADYDDATKRCEQLQSEPDGSYI   43  E5EVW3  E5EVW3_BOMMO
164   CPDYELPDYLQEPTMMDSHVAFLNKQLLMDLLFKCVSTGDYDKAVTITKSLQDDNVGF-M  222  B5BSX5  B5BSX5_BOMMO
170   SPNAALPRELQGLTYYNSHVAFLDNHNFEEEVYNSVINGDYDAAVNMAQSYGVASNSE-F  228  Q76IB6  Q76IB6_PSESE
179   GAGYELPADLKTQTEFSTKMVFADARSINDHLYNLVTGGDYINAVKTVRSLDDNQGSG-V  237  Q2PQU4  Q2PQU4_BOMMO
                                     :   :   :   *            .
```

```
61    IKEAVKRLIENGKRNTMDFAYQLWT--KDGKEIVKSYFPIQFRVIFTEQT-VKLINKRDH  117  P09334  LP1_BOMMO
61    IKEAVKRLIENGKRNTMDFAYQLWT--KDGKEIVKSYFPIQFRVIFTEQT-VKLINNRDH  117  C7A8A2  C7A8A2_BOMMO
61    IKEAVKRLIENGKRNTMDFAYQLWT--KDGKEIVKSYFPIQFRVIFTEQT-VKLINKRDH  117  Q17185  Q17185_BOMMO
66    ITIIVNRLIRENKRNICDLAYKLWDYMDESQEIVKEYFPVIFRQIFSENS-VKIINKRDN  124  Q75RW3  Q75RW3_BOMMO
64    IEDTVNNLIRDGNRNVLEFAYKLW--IGEGKEIVKHYFPVQFRQVLSESN-VKIINKRDN  120  Q05432  Q05432_BOMMO
52    ITEAVNRLIRDSQRNTMEYAYQLWS--LEARDIVKERFPIQFRMMLGEHS-IKLINKRDN  108  Q0VJU3  Q0VJU3_MANSE
52    ITEAVNRLIRDSQRNTMEYAYQLWS--LEARDIVKERFPIQFRMMLGEHS-IKLINKRDN  108  P19616  VITM_MANSE
59    ITNVVNKLIRNNKMNCMEYAYQLWL--QGSKDIVRDCFPVEFRLIFAENA-IKLMYKRDG  115  P09336  LP3_BOMMO
59    ITNVVNKLIRNNKMNCMEYAYQLWL--QGSKDIVRDCFPVEFRLIFAENA-IKLMYKRDG  115  Q00802  L301_BOMMO
59    ITNVVSKLIRNNKMNCMEYAYQLWL--QGSKDIVRDCFPVEFRLIFAENA-IKLMYKRDG  115  C7A8A3  C7A8A3_BOMMO
40    ITNVVNKLIRNNKMNCMEYAYQLWL--QGSKDIVRDCFPVEFRLIFAENA-IKLMYKRDG   96  D4QGB9  D4QGB9_BOMMO
42    ITNVVNKLIRSNKMNCMEYAYQLWL--QGSKDIVRDCFPVEFRLIFAENA-IKLMYKRDG   98  Q6Q0S8  Q6Q0S8_BOMMO
40    ITNVVNKLIRNNKMNCMEYAYQLWL--QGSKDIVRDCFPVEFRLIFAENA-IKLMYKRDG   96  D4QGC0  D4QGC0_BOMMO
59    ITNVVNKLIRNNKMNCMEYAYQLWL--QGSKDIVRDCFPVEFRLIFAENA-IKLMYKRDG  115  E5EVW2  E5EVW2_BOMMO
65    VQNVVNNLIIDKRRNTMEYCYKLWV--GNGQDIVKKYFPLSFRLIMAGNY-VKLIYRNYN  121  P09335  LP2_BOMMO
62    IQNVVNNLIIDKRRNTMEYCYKLWV--GNGQEIVRKYFPLNFRLIMAGNY-VKIIYRNYN  118  A7LIK7  A7LIK7_BOMMO
65    IQNVVNNLIIDKRRNTMEYCYKLWV--GNGQEIVRKYFPLNFRLIMAGNY-VKIIYRNYN  121  P09338  LP5_BOMMO
65    IQNVVNNLIIDGSRNTMEYCYKLWV--GNGQHIVRKYFPYNFRLIMAGNF-VKLIYRNYN  121  P09337  LP4_BOMMO
65    IQNVVNNLIIDGSRNTMEYCYKLWV--GNGQHIVRKYFPYNFRLIMAGNF-VKLIYRNYN  121  Q00801  L302_BOMMO
44    IKNTVTELLNNAESNTINFSYKLWT--TGHQNIVQSCFPLEFRLILDDKKDCKIINKHDD  101  E5EVW3  E5EVW3_BOMMO
223   IEELIDRLLRAREPNVFAYADKLWS--AGHHDIVNDFFPSEVKLITKQER-VKIIGRYYN  279  B5BSX5  B5BSX5_BOMMO
229   TNRIVTRLMTAFPRKLMSFAYKLWH--GGAKEIVRNHFPKAFQHIFNEDA-VTIVNKQYQ  285  Q76IB6  Q76IB6_PSESE
238   CRDVVSRLVSQGIKNAMSFAYKLWH--EGHKDIVEDYFPSEFQLILDQKR-IKLIGNHYN  294  Q2PQU4  Q2PQU4_BOMMO
          : .*:     :    . :**      :.**.  **  .: :       .:: .

118   HALKLIDQQNHN--KIAFGDSKD--KTSKKVSWK-FTPVLENNRVYFKIMSTEDKQYLKL  172  P09334  LP1_BOMMO
118   HALKLIDQQNHN--KIAFGDSKD--KTSKKVSWK-FTPVLENNRVYFKIMSTEDKQYLKL  172  C7A8A2  C7A8A2_BOMMO
118   HALKLIDQQNHN--KIAFGGSKD--KTSKKVSWK-FTPVLENNRVYFKIMSTEDKQYLKL  172  Q17185  Q17185_BOMMO
125   LAIKLGDALDSDNDRVAYGDAND--KTSDNVAWK-LIPLWDDNRVYFKIFSVHRNQIFEI  181  Q75RW3  Q75RW3_BOMMO
121   LAIKLGAAADSDNDRIAYGDAND--KSSENVSWK-LIPLWENNRVYFKIYSVRRHQYLKL  177  Q05432  Q05432_BOMMO
109   LAMKLGVATDNSGDRIAYGAADD--KTSDRVAWK-FVPLSEDKRVYFKILNVQRGQYLKL  165  Q0VJU3  Q0VJU3_MANSE
109   LAMKLGVATDNSGDRIAYGAADD--KTSDRVAWK-FVPLSEDKRVYFKILNVQRGQYLKL  165  P19616  VITM_MANSE
116   LALTLSNDVQGDDGRPAYG--KD--KTSPRVSWK-LIALWENNKVYFKILNTERNQYLVL  170  P09336  LP3_BOMMO
116   LALTLSNDVQGDDGRPRYGDGKD--KTSPRVSWK-LIALWENNKVYFKILNTERNQYLVL  172  Q00802  L301_BOMMO
116   LALTLSNDVQGDDGRPAYGDGKD--KTSPRVSWK-LIALWENNKVYFKILNTERNQYLVL  172  C7A8A3  C7A8A3_BOMMO
97    LALTLSNDVQGDDGRPAYGDGKD--KTSPRVSWK-LIALWENNKVYFKILNTERNQYLVL  153  D4QGB9  D4QGB9_BOMMO
99    LAVTLSNDVQGDDGRPAYGDGKD--KTSPRVSWK-LIALWENNKVYFKILNTERNQYLVL  155  Q6Q0S8  Q6Q0S8_BOMMO
97    LALTLSNDVHGNDGRLAFGDGKD--KTSPKVSWK-FIALWENNKVYFKILNTERNQYLVL  153  D4QGC0  D4QGC0_BOMMO
116   LALTLSNDVHGNDGRLAFGDGKD--KTSPKVSWK-FIALWENNKVYFKILNTERNQYLVL  172  E5EVW2  E5EVW2_BOMMO
122   LALKLGSTTNPSNERIAYGDGVD--KHTDLVSWK-FITLWENNRVYFKAHNTKYNQYLKM  178  P09335  LP2_BOMMO
119   LALKLGSTTNPSNERIAYGDGVD--KHTELVSWK-FITLWENNRVYFKIHNTKYNQYLKM  175  A7LIK7  A7LIK7_BOMMO
122   LALKLGSTTNPSNERIAYGDGVD--KHTELVSWK-FITLWENNRVYFKIHNTKYNQYLKM  178  P09338  LP5_BOMMO
122   LALKLGPTLDPANERLAYGDGKE--KNSDLISWKSHYLVGEQHSVLQDPPTLSYNQYLKL  179  P09337  LP4_BOMMO
122   LALKLGPTLDPANERLAYGDGKE--KNSDLISWK-FITLWENNRVYFKIHNTKYNQYLKL  178  Q00801  L302_BOMMO
102   LYMTLSKDLDQNGDRDAYGDEDD--HKN---SWK-FMSSWESNRVYFKIFNPKYNQRLKM  155  E5EVW3  E5EVW3_BOMMO
280   QALKLDSNVDSYNNRLAWGDSKD--KTSHRVSWK-FIPVWENNKLLYKILNTEYTMYLKL  336  B5BSX5  B5BSX5_BOMMO
286   QPLKLDVNTDSMNDRLAWGDHNQCKITSERLSWK-ILPMWNRDGLTFKLYNVHRNMYLKL  344  Q76IB6  Q76IB6_PSESE
295   QALKLDANVDRYKDRLTWGDGKD--YTSYRVSWR-LISLWENNNVIFKILNTEHEMYLKL  351  Q2PQU4  Q2PQU4_BOMMO
        :.*    .    :  :*   :    .   :*:      : . :  .  .      : :
```

```
173  DNTKGSSD--DRIIYGDSTADTFKHHWYLEPSMYESDV--MFFVYNREYN-SVMTLDEDM  227  P09334   LP1_BOMMO
173  DNTKGSSD--DRIIYGDSTADTFKHHWYLEPSMYESDV--MFFVYNREYN-SVMTLDEDM  227  C7A8A2   C7A8A2_BOMMO
173  DNTKGSSD--DRIIYGDSTADTFKHQWYLEPSMYESDV--MFFVYNREYN-SVMTLDEDM  227  Q17185   Q17185_BOMMO
182  RHTYLTVDN-DHGVYGDDRADTHRHQWYLNPVELENQV--LFYIYNRQYD-QALKLGRNV  237  Q75RW3   Q75RW3_BOMMO
178  G-TGTDGEN-DHSVYGDDRADTHRHQWYLKPAKLDNQV--LFYIYNRQYN-QALKLSRSV  232  Q05432   Q05432_BOMMO
166  G-VETDSDG-EHMAYASSGADTFRHQWYLQPAKADGNL--VFFIVNREYN-HALKLGRSV  220  Q0VJU3   Q0VJU3_MANSE
166  G-VETDSDG-EHMAYASSGADTFRHQWYLQPAKADGNL--VFFIVNREYN-HALKLGRSV  220  P19616   VITM_MANSE
171  GVGT-NWNG-DHMAFGVNSVDSFRAQWYLQPAKYDNDV--LFYIYNREYS-KALTLSRTV  225  P09336   LP3_BOMMO
173  GVGT-NWNG-DHMAFGVNSVDSFRAQWYLQPAKYDNDV--LFYIYNREYS-KALTLSRTV  227  Q00802   L301_BOMMO
173  GVGT-NWNG-DHMAFGVNSVDSFRAQWYLQPAKYDNDV--LFYIYNREYS-KALTLSRTV  227  C7A8A3   C7A8A3_BOMMO
154  GVGT-NWNG-DHMAFGVNSVDSFRAQWYLQPAKYDNDV--LFYIYNREYS-KALTLSRTV  208  D4QGB9   D4QGB9_BOMMO
156  GVGT-NWNG-DHMAFGVNSVDSFRAQWYLQPAKYDNDV--LFYIYNREYS-KALTLSRTV  210  Q6Q0S8   Q6Q0S8_BOMMO
154  GVGT-NPNG-DHMAFGVNSVDSFRAQWYLQPAKYDKDN--LFYIYNREYS-KALTLSRTL  208  D4QGC0   D4QGC0_BOMMO
173  GVGT-NPNG-DHMAFGVNSVDSFRAQWYLQPAKYDKDN--LFYIYNREYS-KALTLSRTL  227  E5EVW2   E5EVW2_BOMMO
179  STSTCNCNARDRVVYGGNSADSTREQWFFQPAKYENDV--LFFIYNRQFN-DALELGTIV  235  P09335   LP2_BOMMO
176  STTTCNCNSRDRVVYGGNSADSTREQWFFQPAKYENDV--LFFIYNRQFN-DALELGTIV  232  A7LIK7   A7LIK7_BOMMO
179  STTTCNCNSRDRVVYGGNSADSTREQWFFQPAKYENDV--LFFIYNRQFN-DALELGTIV  235  P09338   LP5_BOMMO
180  SSTT-DCNTQDRIIFGTNTADTTREQWFLQPTKYENDV--LFFIYNREVQRVALKLGRIV  236  P09337   LP4_BOMMO
179  SSTT-DCNTQDRVIFGTNTADTTREQWFLQPTKYENDV--LFFIYNREYN-DALKLGRIV  234  Q00801   L302_BOMMO
156  GDPVKDDER---KVFSSDDATDSTSQWYLQAMNHKGDL--LFFIFNRRYS-QALKIGKDV  209  E5EVW3   E5EVW3_BOMMO
337  DMNVEEYGD--RKAWGSNNSNEKGHLWKLTPVVLETGN--VLLIENHEYG-QSLKLDAHV  391  B5BSX5   B5BSX5_BOMMO
345  DASVDSMGD--RQAWGSNNSNEDRHRYYLEPMISPHNGTLVFFIINYKYG-QGLKLDAST  401  Q76IB6   Q76IB6_PSESE
352  DVNVDRYGD--RKTWGSNDSSEKRHTWYLYPVKVGDQQ--LFLIENREYR-QGLKLDANV  406  Q2PQU4   Q2PQU4_BOMMO
                   :. .        : : .         :: : * .     : :.

228  AANEDREALGHSGEVSGYPQLFAWYIVPY--  256  P09334   LP1_BOMMO
228  AANEDREALGHSGEVSGYPQLFAWYIVPY--  256  C7A8A2   C7A8A2_BOMMO
228  AANEDREALGHSGEVSGYPQLFAWYIVPY--  256  Q17185   Q17185_BOMMO
238  DSDGDRRAYSSSSSVEGQPELYAWSISILN-  267  Q75RW3   Q75RW3_BOMMO
233  DSDGDRRAYSSSSSVEGQPELFGWSISILN-  262  Q05432   Q05432_BOMMO
221  DSMGDRQVWGHNGNVIGNPELFGWSVVAFFV  251  Q0VJU3   Q0VJU3_MANSE
221  DSMGDRQVWGHNGNVIGNPELFGWSVVAF--  249  P19616   VITM_MANSE
226  EPSGHRMAWGYNGRVIGSPEHYAWGIKAF--  254  P09336   LP3_BOMMO
228  EPSGHRMAWGYNGRVIGSPEHYAWGIKAF--  256  Q00802   L301_BOMMO
228  EPSGHRMSWGYNGRVIGSPEHYAWGIKAF--  256  C7A8A3   C7A8A3_BOMMO
209  EPSGHRMAWGYNGRVIGSPEHYAWGIKAF--  237  D4QGB9   D4QGB9_BOMMO
211  EPSGHRMAWGHNGRVIGSPEHYAWGIKAF--  239  Q6Q0S8   Q6Q0S8_BOMMO
209  ETSGNRMAWGYNGRVIGSPEHYAWGVKAF--  237  D4QGC0   D4QGC0_BOMMO
228  ETSGNRMAWGYNGRVIGSPEHYAWGVKAF--  256  E5EVW2   E5EVW2_BOMMO
236  NASGDRKAVGHDGEVAGLPDIYSWFITPF--  264  P09335   LP2_BOMMO
233  YASGDRKAVGHDGEVAGLPDIYSWFITPF--  261  A7LIK7   A7LIK7_BOMMO
236  NASGDRKAVGHDGEVAGLPDIYSWFITPF--  264  P09338   LP5_BOMMO
237  DASGDRSG------------IWTRWMK----  251  P09337   LP4_BOMMO
235  DASGDRMAFGHDGEVAGLPDIFSWFVTPF--  263  Q00801   L302_BOMMO
210  VNNEDFRIYGESEDAAEKPHYFGWLIEPM--  238  E5EVW3   E5EVW3_BOMMO
392  DSYGDRLLWGNNGNVDGNPGYFGWVINAWQ-  421  B5BSX5   B5BSX5_BOMMO
402  DDIGDRLLWGHNGTVYNEYERFRWIISAW--  430  Q76IB6   Q76IB6_PSESE
407  DRYGDRLVWGNNGTVADNPEYYGFIIQPWQ-  436  Q2PQU4   Q2PQU4_BOMMO
         .                :   :
```
