# Supplementary material for: A proteomic view on the developmental transfer of homologous 30 kDa lipoproteins from peripheral fat body to perivisceral fat body via hemolymph in silkworm, Bombyx mori
Source: BMC Biochem. 2012 Feb 28;13:5. doi: 10.1186/1471-2091-13-5 (PMC3306753; doi:10.1186/1471-2091-13-5)
Supplement: Additional file 9 — Mafft (v6.857b) alignment for C7A8A2 and Q17185. C7A8A2 is identical to LP1 (P09334) except for the presence of N instead of K in position 114 (marked in blue) and was therefore removed from the considerations discussed in the main text. [file 1471-2091-13-5-S9.PDF]

Additional file 9 - Mafft (v6.857b) alignment for C7A8A2 and Q17185. C7A8A2 is identical to LP1 (P09334) except for the presence of N instead of K in position 114 (marked in blue) and was therefore removed from the considerations discussed in the main text.

```
tr|C7A8A2| MRLTLFAFVLAVCALASNATLAPRTDDVLAEQLYMSVVIGEYETAIAKCSEYLKEKKGEV
tr|Q17185| MRLTLFAFVLAVCALASNATLAPRTDDVLAEQLYMSVVIGEYETAIAKCSEYLKEKKGEV

tr|C7A8A2| IKEAVKRLIENGKRNTMDFAYQLWTKDGKEIVKSYFPIQFRVIFTEQTVKLINNRDHHAL
tr|Q17185| IKEAVKRLIENGKRNTMDFAYQLWTKDGKEIVKSYFPIQFRVIFTEQTVKLINKRDHHAL

tr|C7A8A2| KLIDQQNHNKIAFGDSKDKTSKKVSWKFTPVLENNRVYFKIMSTEDKQYLKLDNTKGSSD
tr|Q17185| KLIDQQNHNKIAFGGSKDKTSKKVSWKFTPVLENNRVYFKIMSTEDKQYLKLDNTKGSSD

tr|C7A8A2| DRIIYGDSTADTFKHHWYLEPSMYESDVMFFVYNREYNSVMTLDEDMAANEDREALGHSG
tr|Q17185| DRIIYGDSTADTFKHQWYLEPSMYESDVMFFVYNREYNSVMTLDEDMAANEDREALGHSG

tr|C7A8A2| EVSGYPQLFAWYIVPY
tr|Q17185| EVSGYPQLFAWYIVPY
```
