# Supplementary material for: A proteomic view on the developmental transfer of homologous 30 kDa lipoproteins from peripheral fat body to perivisceral fat body via hemolymph in silkworm, Bombyx mori
Source: BMC Biochem. 2012 Feb 28;13:5. doi: 10.1186/1471-2091-13-5 (PMC3306753; doi:10.1186/1471-2091-13-5)
Supplement: Additional file 10 — Mafft (v6.857b) alignment for C7A8A3 and D4QGB9. [file 1471-2091-13-5-S10.PDF]

Additional file 10 - Mafft (v6.857b) alignment for C7A8A3 and D4QGB9.

```
tr|D4QGB9| -------------------SDVPNDILEEQLYNSVVVADYDSAVEKSKHLYEEKKSEVIT
tr|C7A8A3| MKPAIVILCLFVASLYAADSDVPNDILEEQLYNSVVVADYDSAVEKSKHLYEEKKSEVIT

tr|D4QGB9| NVVNKLIRNNKMNCMEYAYQLWLQGSKDIVRDCFPVEFRLIFAENAIKLMYKRDGLALTL
tr|C7A8A3| NVVSKLIRNNKMNCMEYAYQLWLQGSKDIVRDCFPVEFRLIFAENAIKLMYKRDGLALTL

tr|D4QGB9| SNDVQGDDGRPAYGDGKDKTSPRVSWKLIALWENNKVYFKILNTERNQYLVLGVGTNWNG
tr|C7A8A3| SNDVQGDDGRPAYGDGKDKTSPRVSWKLIALWENNKVYFKILNTERNQYLVLGVGTNWNG

************************************************************

tr|D4QGB9| DHMAFGVNSVDSFRAQWYLQPAKYDNDVLFYIYNREYSKALTLSRTVEPSGHRMAWGYNG
tr|C7A8A3| DHMAFGVNSVDSFRAQWYLQPAKYDNDVLFYIYNREYSKALTLSRTVEPSGHRMSWGYNG

tr|D4QGB9| RVIGSPEHYAWGIKAF
tr|C7A8A3| RVIGSPEHYAWGIKAF
```
