# Supplementary material for: A proteomic view on the developmental transfer of homologous 30 kDa lipoproteins from peripheral fat body to perivisceral fat body via hemolymph in silkworm, Bombyx mori
Source: BMC Biochem. 2012 Feb 28;13:5. doi: 10.1186/1471-2091-13-5 (PMC3306753; doi:10.1186/1471-2091-13-5)
Supplement: Additional file 11 — Mafft (v6.857b) alignment for L301 (Q00802) and LP3 (P09336). [file 1471-2091-13-5-S11.PDF]

Additional file 11 - Mafft (v6.857b) alignment for L301 (Q00802) and LP3 (P09336).

```
sp|Q00802|L301 MKPAIVILCLFVASLYAADSDVPNDILEEQLYNSVVVADYDSAVEKSKHLYEEKKSEVIT
sp|P09336|LP3  MKPAIVILCLFVASLYAADSDVPNDILEEQLYNSVVVADYDSAVEKSKHLYEEKKSEVIT

sp|Q00802|L301 NVVNKLIRNNKMNCMEYAYQLWLQGSKDIVRDCFPVEFRLIFAENAIKLMYKRDGLALTL
sp|P09336|LP3  NVVNKLIRNNKMNCMEYAYQLWLQGSKDIVRDCFPVEFRLIFAENAIKLMYKRDGLALTL

sp|Q00802|L301 SNDVQGDDGRPRYGDGKDKTSPRVSWKLIALWENNKVYFKILNTERNQYLVLGVGTNWNG
sp|P09336|LP3  SNDVQGDDGRPAY--GKDKTSPRVSWKLIALWENNKVYFKILNTERNQYLVLGVGTNWNG

sp|Q00802|L301 DHMAFGVNSVDSFRAQWYLQPAKYDNDVLFYIYNREYSKALTLSRTVEPSGHRMAWGYNG
sp|P09336|LP3  DHMAFGVNSVDSFRAQWYLQPAKYDNDVLFYIYNREYSKALTLSRTVEPSGHRMAWGYNG

sp|Q00802|L301 RVIGSPEHYAWGIKAF
sp|P09336|LP3  RVIGSPEHYAWGIKAF
```
