# Supplementary material for: A proteomic view on the developmental transfer of homologous 30 kDa lipoproteins from peripheral fat body to perivisceral fat body via hemolymph in silkworm, Bombyx mori
Source: BMC Biochem. 2012 Feb 28;13:5. doi: 10.1186/1471-2091-13-5 (PMC3306753; doi:10.1186/1471-2091-13-5)
Supplement: Additional file 12 — Clustal format Mafft (v6.857b) alignment for lipoprotein Blast matches to LP1. [file 1471-2091-13-5-S12.PDF]

Additional file 12 - Clustal format Mafft (v6.857b) alignment for lipoprotein Blast matches to LP1.

Nearly identical sequences were excluded (C7A8A2, see Fig. S3; Q0VJU3_MANSE is C-terminally longer by two amino acids than VITM_MANSE (P19616) and was retained for comparison to *Bombyx mori* sequences). Marked in yellow: sequence identities with LP1. Marked in green: sequences identities with L302. Marked in pink: sequence identities with LP3. Accession numbers in red represent related proteins from *Manduca sexta* and *Pseudaletia separata*.

```
sp|P09334|LP1__ M-----------------------------------------------------------
tr|Q17185|_____ M-----------------------------------------------------------
sp|Q00801|L302_ M-----------------------------------------------------------
tr|A7LIK7|_____ M-----------------------------------------------------------
sp|P09335|LP2__ M-----------------------------------------------------------
sp|P09338|LP5__ M-----------------------------------------------------------
sp|P09337|LP4__ M-----------------------------------------------------------
tr|Q0VJU3|_____ M-----------------------------------------------------------
tr|Q05432|_____ M-----------------------------------------------------------
tr|Q75RW3|_____ M-----------------------------------------------------------
tr|E5EVW2|_____ M-----------------------------------------------------------
tr|D4QGC0|_____ ------------------------------------------------------------
tr|D4QGB9|_____ ------------------------------------------------------------
tr|C7A8A3|_____ M-----------------------------------------------------------
sp|Q00802|L301_ M-----------------------------------------------------------
sp|P09336|LP3__ M-----------------------------------------------------------
tr|Q6Q0S8|_____ ------------------------------------------------------------
tr|Q2PQU4|_____ MTTSFSHSQVKTPSEEKWEAASEPDYHTNEDLLYPYSPIPYFGMYHLVKIPIG-RGLVHH
tr|B5BSX5|_____ M---------------MDPQDKLPNYVTNVDLKYPYSDLPYIGQYKLLKLPFT-GKLIEH
tr|Q76IB6|_____ M-----------RSLTIWKLAQRPNFNTNINKQFPYSETPYQGDYYLEKIPISLNNLIQH
tr|E5EVW3|_____ M-----------------------------------------------------------
```

```
sp|P09334|LP1__  --------------------------------------------------------RLTL
tr|Q17185|_____  --------------------------------------------------------RLTL
sp|Q00801|L302_  -----------------------------------------------------------K
tr|A7LIK7|_____  -----------------------------------------------------------K
sp|P09335|LP2__  -----------------------------------------------------------K
sp|P09338|LP5__  -----------------------------------------------------------K
sp|P09337|LP4__  -----------------------------------------------------------K
tr|Q0VJU3|_____  ----------------------------------------------------------LR
tr|Q05432|_____  -----------------------------------------------------------K
tr|Q75RW3|_____  -----------------------------------------------------------K
tr|E5EVW2|_____  -----------------------------------------------------------K
tr|D4QGC0|_____  ------------------------------------------------------------
tr|D4QGB9|_____  ------------------------------------------------------------
tr|C7A8A3|_____  -----------------------------------------------------------K
sp|Q00802|L301_  -----------------------------------------------------------K
sp|P09336|LP3__  -----------------------------------------------------------K
tr|Q6Q0S8|_____  ------------------------------------------------------------
tr|Q2PQU4|_____  VDYWGEGKVTNLDRVRGFRRSYNVNEQFALVSKGHSKGKQIPNRIPVVSVDDSDTSSYIR
tr|B5BSX5|_____  VDYWGEGSIVNGGLYSGFRNCYNVNRQYQEVSNGPDMGRKIPNRIPVRDENDCDTRAYIK
tr|Q76IB6|_____  VDYWGEGKVVTEEGVRGFSNCYNVNHQYQLVSSGPDKDRKIPNRIPVRSDEDCDTSSYIK
tr|E5EVW3|_____  ------------------------------------------------------------
```

```
sp|P09334|LP1__   FAFVLAVCALASN-----------------------------------------------
tr|Q17185|______  FAFVLAVCALASN-----------------------------------------------
sp|Q00801|L302_   FLVVFASCVLAVS-----------------------------------------------
tr|A7LIK7|______  LLVVFAMCMLAAS-----------------------------------------------
sp|P09335|LP2__   LLVVFAMCVPAAS-----------------------------------------------
sp|P09338|LP5__   FLVVFAVVRACVT-----------------------------------------------
sp|P09337|LP4__   FVVVFASCVLAVS-----------------------------------------------
tr|Q0VJU3|______  TTVVLLTLAAIAF-----------------------------------------------
tr|Q05432|______  FLVFFSTCVLAAS-----------------------------------------------
tr|Q75RW3|______  TLAVLALCLVAAS-----------------------------------------------
tr|E5EVW2|______  PVIVILCLFVASL-----------------------------------------------
tr|D4QGC0|______  ------------------------------------------------------------
tr|D4QGB9|______  ------------------------------------------------------------
tr|C7A8A3|______  PAIVILCLFVASL-----------------------------------------------
sp|Q00802|L301_   PAIVILCLFVASL-----------------------------------------------
sp|P09336|LP3__   PAIVILCLFVASL-----------------------------------------------
tr|Q6Q0S8|______  ------------------------------------------------------------
tr|Q2PQU4|______  DGGVKTVTISTGP-ISKRCAADVARIVNASEGLVVAYGYSENSDDIQNLERELGKKGLYY
tr|B5BSX5|______  DDSVKIVTLMSAP-IIPNSARDITRIVNERVGMVVIYGMPVESQGIKLLAAELKSKLLLY
tr|Q76IB6|______  DNSVLTVTVAEASRITSSCAKDIARIINSDHGKVIVYGVQGNSQEISELAVELRKKGLTP
tr|E5EVW3|______  ------------------------------------------------------------
```

```
sp|P09334|LP1__ --------A---------TLAPRTDDVLAEQLYMSVVIGEYETAIAKCSEYLKEKKGE-V
tr|Q17185|_____ --------A---------TLAPRTDDVLAEQLYMSVVIGEYETAIAKCSEYLKEKKGE-V
sp|Q00801|L302_ --------A--GVAEMSAVSMSSSNKELEEKLYNSILTGDYDSAVRQSLEYENQGKGS-I
tr|A7LIK7|_____ --------A--GVVELSA---DTSNQDLEEKLYNSILTGDYDSAVRQSLEYESQGKGS-I
sp|P09335|LP2__ --------A--GVVELSADSMSPSNQDLEDKLYNSILTGDYDSAVRKSLEYESQGQGS-I
sp|P09338|LP5__ --------P--ACAEMSAVSMSSSNKELEEKLYNSILTGDYDSAVRQSLEYESQGKGS-I
sp|P09337|LP4__ --------A--GVTEMSAASMSSSNKELEEKLYNSILTGDYDSAVRQSLEYENQGKGS-I
tr|Q0VJU3|_____ ------------------------AAPTSDDIYNNVVIGDIDGAVAKSKELQKQGKGD-I
tr|Q05432|_____ --------A--GLIDLDINILSAPTR-AETRLVDAITTADYNTAVSLILLLEKQSSGS-I
tr|Q75RW3|_____ --------ATPSIDGDDRYPIHAPSG-YEDIVTNAIITRNYEAAASMTVQLKRRSSGR-Y
tr|E5EVW2|_____ ----------------YAADSDVPNDILEEQLYNSIVVADYDSAVEKSKHLYEEKKSE-V
tr|D4QGC0|_____ --------------------SDVPNDILEEQLYNSIVVADYDSAVEKSKHLYEEKKSE-V
tr|D4QGB9|_____ --------------------SDVPNDILEEQLYNSVVVADYDSAVEKSKHLYEEKKSE-V
tr|C7A8A3|_____ ----------------YAADSDVPNDILEEQLYNSVVVADYDSAVEKSKHLYEEKKSE-V
sp|Q00802|L301_ ----------------YAADSDVPNDILEEQLYNSVVVADYDSAVEKSKHLYEEKKSE-V
sp|P09336|LP3__ ----------------YAADSDVPNDILEEQLYNSVVVADYDSAVEKSKHLYEEKKSE-V
tr|Q6Q0S8|_____ ------------------ADSDVPNDILEEQLYNSVVVADYDSAVEKSKHLYGEKKSE-V
tr|Q2PQU4|_____ GAGYELPADLKTQTEFSTKMVFADARSINDHLYNLVTGGDYINAVKTVRSLDDNQGSG-V
tr|B5BSX5|_____ CPDYELPDYLQEPTMMDSHVAFLNKQLLMDLLFKCVSTGDYDKAVTITKSLQDDNVGF-M
tr|Q76IB6|_____ SPNAALPRELQGLTYYNSHVAFLDNHNFEEEVYNSVINGDYDAAVNMAQSYGVASNSE-F
tr|E5EVW3|_____ ------------------SVEASDSGKSDRKLYNSVITADYDDATKRCEQLQSEPDGSYI
                                               :   :   :   *            .
```

```
sp|P09334|LP1__   IKEAVKRLIENGKRNTMDFAYQLW--TKDGKEIVKSYFPIQFRVIFTEQT-VKLINKRDH
tr|Q17185|______  IKEAVKRLIENGKRNTMDFAYQLW--TKDGKEIVKSYFPIQFRVIFTEQT-VKLINKRDH
sp|Q00801|L302_   IQNVVNNLIIDGSRNTMEYCYKLW--VGNGQHIVRKYFPYNFRLIMAGNF-VKLIYRNYN
tr|A7LIK7|______  IQNVVNNLIIDKRRNTMEYCYKLW--VGNGQEIVRKYFPLNFRLIMAGNY-VKIIYRNYN
sp|P09335|LP2__   VQNVVNNLIIDKRRNTMEYCYKLW--VGNGQDIVKKYFPLSFRLIMAGNY-VKLIYRNYN
sp|P09338|LP5__   IQNVVNNLIIDKRRNTMEYCYKLW--VGNGQEIVRKYFPLNFRLIMAGNY-VKIIYRNYN
sp|P09337|LP4__   IQNVVNNLIIDGSRNTMEYCYKLW--VGNGQHIVRKYFPYNFRLIMAGNF-VKLIYRNYN
tr|Q0VJU3|______  ITEAVNRLIRDSQRNTMEYAYQLW--SLEARDIVKERFPIQFRMMLGEHS-IKLINKRDN
tr|Q05432|______  IEDTVNNLIRDGNRNVLEFAYKLW--IGEGKEIVKHYFPVQFRQVLSESN-VKIINKRDN
tr|Q75RW3|______  ITIIVNRLIRENKRNICDLAYKLWDYMDESQEIVKEYFPVIFRQIFSENS-VKIINKRDN
tr|E5EVW2|______  ITNVVNKLIRNNKMNCMEYAYQLW--LQGSKDIVRDCFPVEFRLIFAENA-IKLMYKRDG
tr|D4QGC0|______  ITNVVNKLIRNNKMNCMEYAYQLW--LQGSKDIVRDCFPVEFRLIFAENA-IKLMYKRDG
tr|D4QGB9|______  ITNVVNKLIRNNKMNCMEYAYQLW--LQGSKDIVRDCFPVEFRLIFAENA-IKLMYKRDG
tr|C7A8A3|______  ITNVVSKLIRNNKMNCMEYAYQLW--LQGSKDIVRDCFPVEFRLIFAENA-IKLMYKRDG
sp|Q00802|L301_   ITNVVNKLIRNNKMNCMEYAYQLW--LQGSKDIVRDCFPVEFRLIFAENA-IKLMYKRDG
sp|P09336|LP3__   ITNVVNKLIRNNKMNCMEYAYQLW--LQGSKDIVRDCFPVEFRLIFAENA-IKLMYKRDG
tr|Q6Q0S8|______  ITNVVNKLIRSNKMNCMEYAYQLW--LQGSKDIVRDCFPVEFRLIFAENA-IKLMYKRDG
tr|Q2PQU4|______  CRDVVSRLVSQGIKNAMSFAYKLW--HEGHKDIVEDYFPSEFQLILDQKR-IKLIGNHYN
tr|B5BSX5|______  IEELIDRLLRAREPNVFAYADKLW--SAGHHDIVNDFFPSEVKLITKQER-VKIIGRYYN
tr|Q76IB6|______  TNRIVTRLMTAFPRKLMSFAYKLW--HGGAKEIVRNHFPKAFQHIFNEDA-VTIVNKQYQ
tr|E5EVW3|______  IKNTVTELLNNAESNTINFSYKLW--TTGHQNIVQSCFPLEFRLILDDKKDCKIINKHDD
                      : .*:     :    . :**      :.**.  **  .: :       .:: .
```

```
sp|P09334|LP1__ HALKLID--QQNHNKIAFGDSKD--KTSKKVSWKFTPV-LENNRVYFKIMSTEDKQYLKL
tr|Q17185|_____ HALKLID--QQNHNKIAFGGSKD--KTSKKVSWKFTPV-LENNRVYFKIMSTEDKQYLKL
sp|Q00801|L302_ LALKLGPTLDPANERLAYGDGKE--KNSDLISWKFITL-WENNRVYFKIHNTKYNQYLKL
tr|A7LIK7|_____ LALKLGSTTNPSNERIAYGDGVD--KHTELVSWKFITL-WENNRVYFKIHNTKYNQYLKM
sp|P09335|LP2__ LALKLGSTTNPSNERIAYGDGVD--KHTDLVSWKFITL-WENNRVYFKAHNTKYNQYLKM
sp|P09338|LP5__ LALKLGSTTNPSNERIAYGDGVD--KHTELVSWKFITL-WENNRVYFKIHNTKYNQYLKM
sp|P09337|LP4__ LALKLGPTLDPANERLAYGDGKE--KNSDLISWKSHYLVGEQHSVLQDPPTLSYNQYLKL
tr|Q0VJU3|_____ LAMKLGVATDNSGDRIAYGAADD--KTSDRVAWKFVPL-SEDKRVYFKILNVQRGQYLKL
tr|Q05432|_____ LAIKLGAAADSDNDRIAYGDAND--KSSENVSWKLIPL-WENNRVYFKIYSVRRHQYLKL
tr|Q75RW3|_____ LAIKLGDALDSDNDRVAYGDAND--KTSDNVAWKLIPL-WDDNRVYFKIFSVHRNQIFEI
tr|E5EVW2|_____ LALTLSNDVHGNDGRLAFGDGKD--KTSPKVSWKFIAL-WENNKVYFKILNTERNQYLVL
tr|D4QGC0|_____ LALTLSNDVHGNDGRLAFGDGKD--KTSPKVSWKFIAL-WENNKVYFKILNTERNQYLVL
tr|D4QGB9|_____ LALTLSNDVQGDDGRPAYGDGKD--KTSPRVSWKLIAL-WENNKVYFKILNTERNQYLVL
tr|C7A8A3|_____ LALTLSNDVQGDDGRPAYGDGKD--KTSPRVSWKLIAL-WENNKVYFKILNTERNQYLVL
sp|Q00802|L301_ LALTLSNDVQGDDGRPRYGDGKD--KTSPRVSWKLIAL-WENNKVYFKILNTERNQYLVL
sp|P09336|LP3__ LALTLSNDVQGDDGRPAY--GKD--KTSPRVSWKLIAL-WENNKVYFKILNTERNQYLVL
tr|Q6Q0S8|_____ LAVTLSNDVQGDDGRPAYGDGKD--KTSPRVSWKLIAL-WENNKVYFKILNTERNQYLVL
tr|Q2PQU4|_____ QALKLDANVDRYKDRLTWGDGKD--YTSYRVSWRLISL-WENNNVIFKILNTEHEMYLKL
tr|B5BSX5|_____ QALKLDSNVDSYNNRLAWGDSKD--KTSHRVSWKFIPV-WENNKLLYKILNTEYTMYLKL
tr|Q76IB6|_____ QPLKLDVNTDSMNDRLAWGDHNQCKITSERLSWKILPM-WNRDGLTFKLYNVHRNMYLKL
tr|E5EVW3|_____ LYMTLSKDLDQNGDRDAYGDEDD--HKN---SWKFMSS-WESNRVYFKIFNPKYNQRLKM
                  :.*    .    :  :    :    .   :*:      : . :  .  .      : :
```

```
sp|P09334|LP1__  DNTK-GSSD-DRIIYGDSTADTFKHHWYLEPS--MYESDVMFFVYNREYNS-VMTLDEDM
tr|Q17185|_____  DNTK-GSSD-DRIIYGDSTADTFKHQWYLEPS--MYESDVMFFVYNREYNS-VMTLDEDM
sp|Q00801|L302_  SSTT-DCNTQDRVIFGTNTADTTREQWFLQPT--KYENDVLFFIYNREYND-ALKLGRIV
tr|A7LIK7|_____  STTTCNCNSRDRVVYGGNSADSTREQWFFQPA--KYENDVLFFIYNRQFND-ALELGTIV
sp|P09335|LP2__  STSTCNCNARDRVVYGGNSADSTREQWFFQPA--KYENDVLFFIYNRQFND-ALELGTIV
sp|P09338|LP5__  STTTCNCNSRDRVVYGGNSADSTREQWFFQPA--KYENDVLFFIYNRQFND-ALELGTIV
sp|P09337|LP4__  SSTT-DCNTQDRIIFGTNTADTTREQWFLQPT--KYENDVLFFIYNREVQRVALKLGRIV
tr|Q0VJU3|_____  GVET-DSDG-EHMAYASSGADTFRHQWYLQPA--KADGNLVFFIVNREYNH-ALKLGRSV
tr|Q05432|_____  GTGT-DGEN-DHSVYGDDRADTHRHQWYLKPA--KLDNQVLFYIYNRQYNQ-ALKLSRSV
tr|Q75RW3|_____  RHTYLTVDN-DHGVYGDDRADTHRHQWYLNPV--ELENQVLFYIYNRQYDQ-ALKLGRNV
tr|E5EVW2|_____  GVGT-NPNG-DHMAFGVNSVDSFRAQWYLQPA--KYDKDNLFYIYNREYSK-ALTLSRTL
tr|D4QGC0|_____  GVGT-NPNG-DHMAFGVNSVDSFRAQWYLQPA--KYDKDNLFYIYNREYSK-ALTLSRTL
tr|D4QGB9|_____  GVGT-NWNG-DHMAFGVNSVDSFRAQWYLQPA--KYDNDVLFYIYNREYSK-ALTLSRTV
tr|C7A8A3|_____  GVGT-NWNG-DHMAFGVNSVDSFRAQWYLQPA--KYDNDVLFYIYNREYSK-ALTLSRTV
sp|Q00802|L301_  GVGT-NWNG-DHMAFGVNSVDSFRAQWYLQPA--KYDNDVLFYIYNREYSK-ALTLSRTV
sp|P09336|LP3__  GVGT-NWNG-DHMAFGVNSVDSFRAQWYLQPA--KYDNDVLFYIYNREYSK-ALTLSRTV
tr|Q6Q0S8|_____  GVGT-NWNG-DHMAFGVNSVDSFRAQWYLQPA--KYDNDVLFYIYNREYSK-ALTLSRTV
tr|Q2PQU4|_____  DVNV-DRYG-DRKTWGSNDSSEKRHTWYLYPV--KVGDQQLFLIENREYRQ-GLKLDANV
tr|B5BSX5|_____  DMNV-EEYG-DRKAWGSNNSNEKGHLWKLTPV--VLETGNVLLIENHEYGQ-SLKLDAHV
tr|Q76IB6|_____  DASV-DSMG-DRQAWGSNNSNEDRHRYYLEPMISPHNGTLVFFIINYKYGQ-GLKLDAST
tr|E5EVW3|_____  GDPV--KDD-ERKVFSSDDATDSTSQWYLQAM--NHKGDLLFFIFNRRYSQ-ALKIGKDV
                           ::  :. .        : : .         :: : * .     : :.
```

```
sp|P09334|LP1__ AANEDREALGHSGEVSGYPQLFAWYIVPY--
tr|Q17185|_____ AANEDREALGHSGEVSGYPQLFAWYIVPY--
sp|Q00801|L302_ DASGDRMAFGHDGEVAGLPDIFSWFVTPF--
tr|A7LIK7|_____ YASGDRKAVGHDGEVAGLPDIYSWFITPF--
sp|P09335|LP2__ NASGDRKAVGHDGEVAGLPDIYSWFITPF--
sp|P09338|LP5__ NASGDRKAVGHDGEVAGLPDIYSWFITPF--
sp|P09337|LP4__ DASGDRSGI--------------WTRWMK--
tr|Q0VJU3|_____ DSMGDRQVWGHNGNVIGNPELFGWSVVAFFV
tr|Q05432|_____ DSDGDRRAYSSSSSVEGQPELFGWSISIL-N
tr|Q75RW3|_____ DSDGDRRAYSSSSSVEGQPELYAWSISIL-N
tr|E5EVW2|_____ ETSGNRMAWGYNGRVIGSPEHYAWGVKAF--
tr|D4QGC0|_____ ETSGNRMAWGYNGRVIGSPEHYAWGVKAF--
tr|D4QGB9|_____ EPSGHRMAWGYNGRVIGSPEHYAWGIKAF--
tr|C7A8A3|_____ EPSGHRMSWGYNGRVIGSPEHYAWGIKAF--
sp|Q00802|L301_ EPSGHRMAWGYNGRVIGSPEHYAWGIKAF--
sp|P09336|LP3__ EPSGHRMAWGYNGRVIGSPEHYAWGIKAF--
tr|Q6Q0S8|_____ EPSGHRMAWGHNGRVIGSPEHYAWGIKAF--
tr|Q2PQU4|_____ DRYGDRLVWGNNGTVADNPEYYGFIIQPW-Q
tr|B5BSX5|_____ DSYGDRLLWGNNGNVDGNPGYFGWVINAW-Q
tr|Q76IB6|_____ DDIGDRLLWGHNGTVYNEYERFRWIISAW--
tr|E5EVW3|_____ VNNEDFRIYGESEDAAEKPHYFGWLIEPM--
                    .                  :
```
