# Supplementary material for: A proteomic view on the developmental transfer of homologous 30 kDa lipoproteins from peripheral fat body to perivisceral fat body via hemolymph in silkworm, Bombyx mori
Source: BMC Biochem. 2012 Feb 28;13:5. doi: 10.1186/1471-2091-13-5 (PMC3306753; doi:10.1186/1471-2091-13-5)

Additional file 13 - Mafft phylogenetic tree of Blast sequence alignment matches for LP1_BOMMO (June 16, 2011).

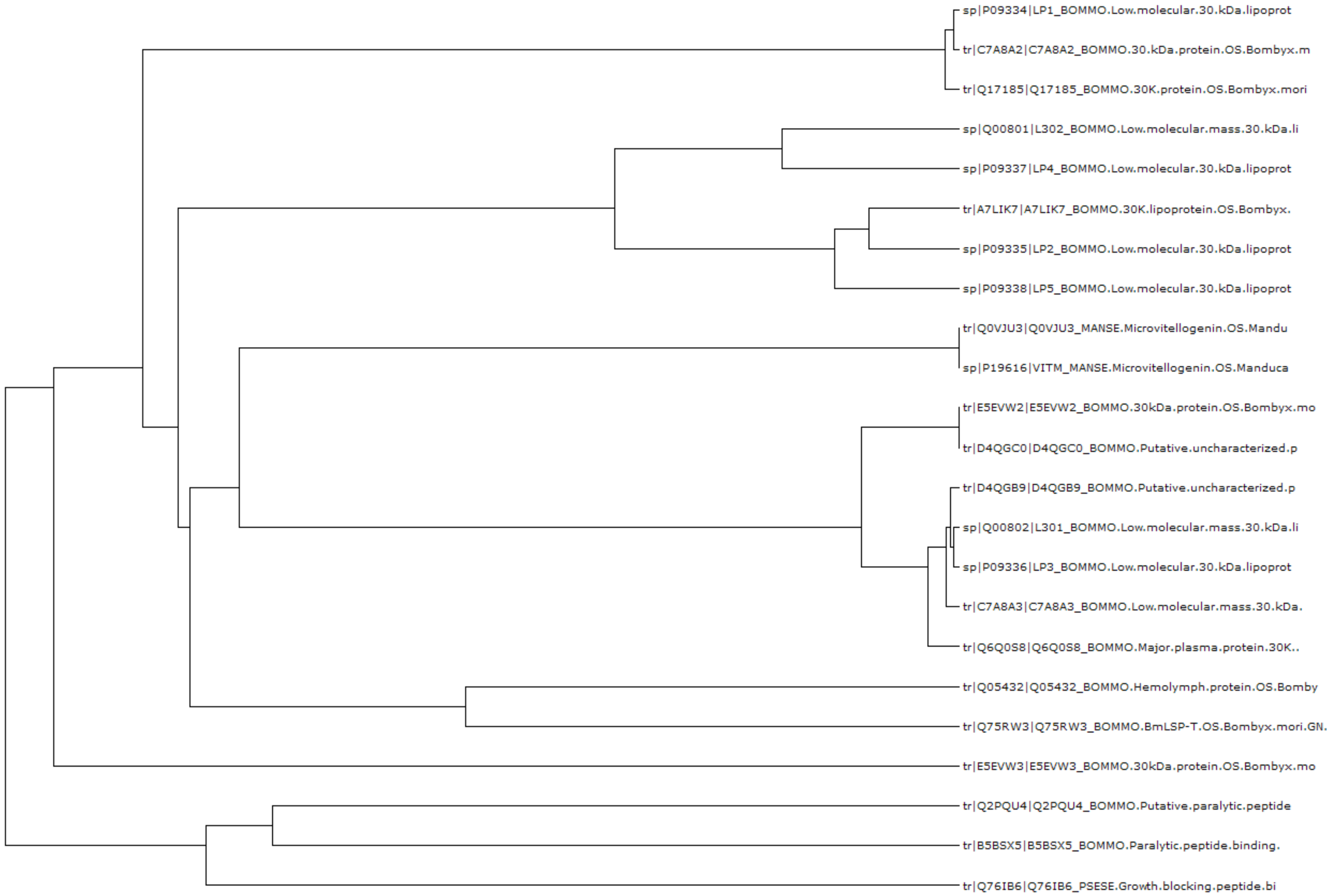

Supplement: Additional file 13 — Mafft phylogenetic tree of Blast sequence alignment matches for LP1_BOMMO (June 16, 2011). [file 1471-2091-13-5-S13.PDF]
