# Supplementary material for: A proteomic view on the developmental transfer of homologous 30 kDa lipoproteins from peripheral fat body to perivisceral fat body via hemolymph in silkworm, Bombyx mori
Source: BMC Biochem. 2012 Feb 28;13:5. doi: 10.1186/1471-2091-13-5 (PMC3306753; doi:10.1186/1471-2091-13-5)
Supplement: Additional file 15 — UniProt entries for proteins assigned to spots excised from the 2D gel map of total proteins isolated from hemolymph of day 3 of V instar larvae of B. mori using LC-MS/MS and database search. [file 1471-2091-13-5-S15.PDF]

Additional file 15 - UniProt entries for proteins assigned to spots excised from the 2D gel map of total proteins isolated from hemolymph of day 3 of V instar larvae of *B. mori* using LC-MS/MS and database search. For more details see Fig. 4. Peptide evidence for specific protein forms was not conclusive so all relevant database entries for the homologous proteins were quoted.

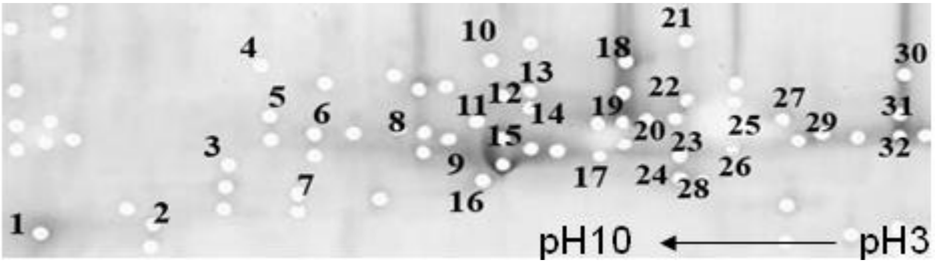

Spot#	Entry	Description
	C7A8A2_BOMMO	30 kDa protein OS Bombyx mori PE 2 SV 1
	LP3_BOMMO	Low molecular 30 kDa lipoprotein PBMHPC 19 OS Bombyx mori GN LP PE 2 SV 1
	LP1_BOMMO	Low molecular 30 kDa lipoprotein PBMHP 6 precursor
	Q05432_BOMMO	Hemolymph protein OS Bombyx mori PE 3 SV 1
1	Q6Q0S8_BOMMO	Major plasma protein 30K Fragment OS Bombyx mori GN 19G PE 4 SV 1
	C7ASM6_BOMMO	Antitrypsin isoform 3 OS Bombyx mori PE 3 SV 1
	ACH2_BOMMO	Antichymotrypsin 2 Antichymotrypsin II ACHY II
2	Q967V9_BOMMO	Chymotrypsin inhibitor CI 8A Fragment OS Bombyx mori PE 2 SV 1
	C7A8A2_BOMMO	30 kDa protein OS Bombyx mori PE 2 SV 1
	LP1_BOMMO	Low molecular 30 kDa lipoprotein PBMHP 6 precursor
	Q05432_BOMMO	Hemolymph protein OS Bombyx mori PE 3 SV 1
	Q0VJU3_MANSE	Microvitellogenin OS Manduca sexta GN MVg PE 2 SV 1
3	VITM_MANSE	Microvitellogenin OS Manduca sexta GN MVG PE 2 SV 1
	C7ASM3_BOMMO	Antitrypsin isoform 2 OS Bombyx mori PE 2 SV 1
	D2X2F6_BOMMO	C type lectin 10 OS Bombyx mori PE 2 SV 1
4	A1AT_BOMMO	Antitrypsin precursor AT
	C7A8A2_BOMMO	30 kDa protein OS Bombyx mori PE 2 SV 1
	LP1_BOMMO	Low molecular 30 kDa lipoprotein PBMHP 6 precursor
	LP2_BOMMO	Low molecular 30 kDa lipoprotein PBMHP 12 precursor
5	Q75RW3_BOMMO	BmLSP T OS Bombyx mori GN BmLSP T PE 2 SV 1
6	A7BEX9_BOMMO	Imaginal disk growth factor OS Bombyx mori GN IDGF PE 2 SV 1

	A7LIK7_BOMMO	30K lipoprotein OS Bombyx mori PE 2 SV 1
	C7A8A2_BOMMO	30 kDa protein OS Bombyx mori PE 2 SV 1
	IDGFL_BOMMO	Chitinase like protein EN03 OS Bombyx mori GN EN03 PE 1 SV 1
	LP1_BOMMO	Low molecular 30 kDa lipoprotein PBMHP 6 precursor
	LP2_BOMMO	Low molecular 30 kDa lipoprotein PBMHP 12 precursor
	Q05432_BOMMO	Hemolymph protein OS Bombyx mori PE 3 SV 1
	C7A8A3_BOMMO	Low molecular mass 30 kDa lipoprotein 19G1 OS Bombyx mori PE 2 SV 1
	SSP1_BOMMO	Sex specific storage protein 1 precursor SP 1 Methionine rich storage protein
	LP3_BOMMO	Low molecular 30 kDa lipoprotein PBMHPC 19 precursor
7	Q6Q0S8_BOMMO	Major plasma protein 30K Fragment OS Bombyx mori GN 19G PE 4 SV 1
	A7LIK7_BOMMO	30K lipoprotein OS Bombyx mori PE 2 SV 1
	LP1_BOMMO	Low molecular 30 kDa lipoprotein PBMHP 6 OS Bombyx mori GN LP PE 2 SV 1
8	LP5_BOMMO	Low molecular 30 kDa lipoprotein PBMHPC 23 precursor
	A7LIK7_BOMMO	30K lipoprotein OS Bombyx mori PE 2 SV 1
	C7A8A2_BOMMO	30 kDa protein OS Bombyx mori PE 2 SV 1
	L302_BOMMO	Low molecular mass 30 kDa lipoprotein 21G1 OS Bombyx mori GN 21G1 PE 2 SV 1
	LP1_BOMMO	Low molecular 30 kDa lipoprotein PBMHP 6 precursor
	LP2_BOMMO	Low molecular 30 kDa lipoprotein PBMHP 12 precursor
	Q05432_BOMMO	Hemolymph protein OS Bombyx mori PE 3 SV 1
	Q1HPR9_BOMMO	Mitochondrial translational release factor 1 OS Bombyx mori PE 2 SV 1
	A7BEX9_BOMMO	Imaginal disk growth factor OS Bombyx mori GN IDGF PE 2 SV 1
9	IDGFL_BOMMO	Chitinase like protein EN03 OS Bombyx mori GN EN03 PE 1 SV 1
	C7ASM6_BOMMO	Antitrypsin isoform 3 OS Bombyx mori PE 3 SV 1
	LP3_BOMMO	Low molecular 30 kDa lipoprotein PBMHPC 19 precursor
	ACH2_BOMMO	Antichymotrypsin 2 Antichymotrypsin II ACHY II
10	Q6Q0S8_BOMMO	Major plasma protein 30K Fragment OS Bombyx mori GN 19G PE 4 SV 1
	A7LIK7_BOMMO	30K lipoprotein OS Bombyx mori PE 2 SV 1
	C7A8A2_BOMMO	30 kDa protein OS Bombyx mori PE 2 SV 1
	C7A8A3_BOMMO	Low molecular mass 30 kDa lipoprotein 19G1 OS Bombyx mori PE 2 SV 1
	L302_BOMMO	Low molecular mass 30 kDa lipoprotein 21G1 OS Bombyx mori GN 21G1 PE 2 SV 1
	LP1_BOMMO	Low molecular 30 kDa lipoprotein PBMHP 6 OS Bombyx mori GN LP PE 2 SV 1
11	LP2_BOMMO	Low molecular 30 kDa lipoprotein PBMHP 12 OS Bombyx mori GN LP PE 2 SV 1

	LP3_BOMMO	Low molecular 30 kDa lipoprotein PBMHPC 19 OS Bombyx mori GN LP PE 2 SV 1
	LP1_BOMMO	Low molecular 30 kDa lipoprotein PBMHP 6 precursor
	LP3_BOMMO	Low molecular 30 kDa lipoprotein PBMHPC 19 precursor
	LP5_BOMMO	Low molecular 30 kDa lipoprotein PBMHPC 23 precursor
	PANB_ACISJ	3 methyl 2 oxobutanoate hydroxymethyltransferase OS Acidovorax sp strain JS42 GN panB PE 3 SV 1
	L302_BOMMO	Low molecular mass 30 kDa lipoprotein 21G1 precursor
	Q6Q0S8_BOMMO	Major plasma protein 30K Fragment OS Bombyx mori GN 19G PE 4 SV 1
	C7A8A2_BOMMO	30 kDa protein OS Bombyx mori PE 2 SV 1
	LP1_BOMMO	Low molecular 30 kDa lipoprotein PBMHP 6 precursor
	LP3_BOMMO	Low molecular 30 kDa lipoprotein PBMHPC 19 precursor
	L302_BOMMO	Low molecular mass 30 kDa lipoprotein 21G1 precursor
	Q05432_BOMMO	Hemolymph protein OS Bombyx mori PE 3 SV 1
12	Q6Q0S8_BOMMO	Major plasma protein 30K Fragment OS Bombyx mori GN 19G PE 4 SV 1
	LP3_BOMMO	Low molecular 30 kDa lipoprotein PBMHPC 19 precursor
13	Q6Q0S8_BOMMO	Major plasma protein 30K Fragment OS Bombyx mori GN 19G PE 4 SV 1
	C7A8A3_BOMMO	Low molecular mass 30 kDa lipoprotein 19G1 OS Bombyx mori PE 2 SV 1
	LP3_BOMMO	Low molecular 30 kDa lipoprotein PBMHPC 19 precursor
14	Q6Q0S8_BOMMO	Major plasma protein 30K Fragment OS Bombyx mori GN 19G PE 4 SV 1
	A7LIK7_BOMMO	30K lipoprotein OS Bombyx mori PE 2 SV 1
	C7A8A2_BOMMO	30 kDa protein OS Bombyx mori PE 2 SV 1
	L302_BOMMO	Low molecular mass 30 kDa lipoprotein 21G1 OS Bombyx mori GN 21G1 PE 2 SV 1
	LP5_BOMMO	Low molecular 30 kDa lipoprotein PBMHPC 23 OS Bombyx mori GN LP PE 2 SV 1
	LP1_BOMMO	Low molecular 30 kDa lipoprotein PBMHP 6 precursor
	LP2_BOMMO	Low molecular 30 kDa lipoprotein PBMHP 12 precursor
	Q05432_BOMMO	Hemolymph protein OS Bombyx mori PE 3 SV 1
	Q538A5_BOMMO	Chorion b ZIP transcription factor OS Bombyx mori GN CbZ PE 2 SV 1
15	VITM_MANSE	Microvitellogenin OS Manduca sexta GN MVG PE 2 SV 1
	C7A8A2_BOMMO	30 kDa protein OS Bombyx mori PE 2 SV 1
	LP3_BOMMO	Low molecular 30 kDa lipoprotein PBMHPC 19 OS Bombyx mori GN LP PE 2 SV 1
	LP1_BOMMO	Low molecular 30 kDa lipoprotein PBMHP 6 precursor
	Q05432_BOMMO	Hemolymph protein OS Bombyx mori PE 3 SV 1
16	Q6Q0S8_BOMMO	Major plasma protein 30K Fragment OS Bombyx mori GN 19G PE 4 SV 1

	A7LIK7_BOMMO	30K lipoprotein OS Bombyx mori PE 2 SV 1
	C7A8A2_BOMMO	30 kDa protein OS Bombyx mori PE 2 SV 1
	LP1_BOMMO	Low molecular 30 kDa lipoprotein PBMHP 6 precursor
	LP2_BOMMO	Low molecular 30 kDa lipoprotein PBMHP 12 precursor
	LP3_BOMMO	Low molecular 30 kDa lipoprotein PBMHPC 19 precursor
	Q0N2R8_BOMMO	Putative signal recognition particle 54 kDa protein OS Bombyx mori GN SRP54 PE 2 SV 1
17	Q6Q0S8_BOMMO	Major plasma protein 30K Fragment OS Bombyx mori GN 19G PE 4 SV 1
	C7A8A3_BOMMO	Low molecular mass 30 kDa lipoprotein 19G1 OS Bombyx mori PE 2 SV 1
	LP3_BOMMO	Low molecular 30 kDa lipoprotein PBMHPC 19 precursor
18	Q6Q0S8_BOMMO	Major plasma protein 30K Fragment OS Bombyx mori GN 19G PE 4 SV 1
	A7LIK7_BOMMO	30K lipoprotein OS Bombyx mori PE 2 SV 1
	C7A8A3_BOMMO	Low molecular mass 30 kDa lipoprotein 19G1 OS Bombyx mori PE 2 SV 1
	LP5_BOMMO	Low molecular 30 kDa lipoprotein PBMHPC 23 OS Bombyx mori GN LP PE 2 SV 1
	LP3_BOMMO	Low molecular 30 kDa lipoprotein PBMHPC 19 precursor
19	Q6Q0S8_BOMMO	Major plasma protein 30K Fragment OS Bombyx mori GN 19G PE 4 SV 1
	C7A8A3_BOMMO	Low molecular mass 30 kDa lipoprotein 19G1 OS Bombyx mori PE 2 SV 1
	LP3_BOMMO	Low molecular 30 kDa lipoprotein PBMHPC 19 precursor
	L302_BOMMO	Low molecular mass 30 kDa lipoprotein 21G1 precursor
20	Q6Q0S8_BOMMO	Major plasma protein 30K Fragment OS Bombyx mori GN 19G PE 4 SV 1
	A7LIK7_BOMMO	30K lipoprotein OS Bombyx mori PE 2 SV 1
	LP2_BOMMO	Low molecular 30 kDa lipoprotein PBMHP 12 precursor
	LP3_BOMMO	Low molecular 30 kDa lipoprotein PBMHPC 19 precursor
	LP5_BOMMO	Low molecular 30 kDa lipoprotein PBMHPC 23 precursor
	L302_BOMMO	Low molecular mass 30 kDa lipoprotein 21G1 precursor
21	Q6Q0S8_BOMMO	Major plasma protein 30K Fragment OS Bombyx mori GN 19G PE 4 SV 1
	A7LIK7_BOMMO	30K lipoprotein OS Bombyx mori PE 2 SV 1
	B5BSX5_BOMMO	Paralytic peptide binding protein 2 OS Bombyx mori GN PPBP2 PE 2 SV 1
	LP3_BOMMO	Low molecular 30 kDa lipoprotein PBMHPC 19 precursor
	LP5_BOMMO	Low molecular 30 kDa lipoprotein PBMHPC 23 precursor
	L302_BOMMO	Low molecular mass 30 kDa lipoprotein 21G1 precursor
22	Q6Q0S8_BOMMO	Major plasma protein 30K Fragment OS Bombyx mori GN 19G PE 4 SV 1
23	A7BEX9_BOMMO	Imaginal disk growth factor OS Bombyx mori GN IDGF PE 2 SV 1

	IDGFL_BOMMO	Chitinase like protein EN03 OS Bombyx mori GN EN03 PE 1 SV 1
	C5J498_BOMMO	Signal transducer and activator of transcription OS Bombyx mori PE 2 SV 1
	ACH1_BOMMO	Antichymotrypsin 1 precursor Antichymotrypsin I ACHY I
	Q1HPJ9_BOMMO	Vacuolar ATP synthase subunit H OS Bombyx mori PE 2 SV 1
24	Q967V9_BOMMO	Chymotrypsin inhibitor CI 8A Fragment OS Bombyx mori PE 2 SV 1
	A7LIK7_BOMMO	30K lipoprotein OS Bombyx mori PE 2 SV 1
	LP5_BOMMO	Low molecular 30 kDa lipoprotein PBMHPC 23 OS Bombyx mori GN LP PE 2 SV 1
	LP3_BOMMO	Low molecular 30 kDa lipoprotein PBMHPC 19 precursor
	LP5_BOMMO	Low molecular 30 kDa lipoprotein PBMHPC 23 precursor
25	Q6Q0S8_BOMMO	Major plasma protein 30K Fragment OS Bombyx mori GN 19G PE 4 SV 1
	A7LIK7_BOMMO	30K lipoprotein OS Bombyx mori PE 2 SV 1
	C7A8A2_BOMMO	30 kDa protein OS Bombyx mori PE 2 SV 1
	LP1_BOMMO	Low molecular 30 kDa lipoprotein PBMHP 6 precursor
	LP3_BOMMO	Low molecular 30 kDa lipoprotein PBMHPC 19 precursor
	LP5_BOMMO	Low molecular 30 kDa lipoprotein PBMHPC 23 precursor
26	L302_BOMMO	Low molecular mass 30 kDa lipoprotein 21G1 precursor
	C7A8A3_BOMMO	Low molecular mass 30 kDa lipoprotein 19G1 OS Bombyx mori PE 2 SV 1
	LP3_BOMMO	Low molecular 30 kDa lipoprotein PBMHPC 19 precursor
	L301_BOMMO	Low molecular mass 30 kDa lipoprotein 19G1 precursor
27	Q6Q0S8_BOMMO	Major plasma protein 30K Fragment OS Bombyx mori GN 19G PE 4 SV 1
	A7LIK7_BOMMO	30K lipoprotein OS Bombyx mori PE 2 SV 1
	C0SQ80_BOMMO	Odorant binding protein OS Bombyx mori GN fmxg2I3 PE 2 SV 1
	LP3_BOMMO	Low molecular 30 kDa lipoprotein PBMHPC 19 precursor
	LP5_BOMMO	Low molecular 30 kDa lipoprotein PBMHPC 23 precursor
28	Q6Q0S8_BOMMO	Major plasma protein 30K Fragment OS Bombyx mori GN 19G PE 4 SV 1
	A7LIK7_BOMMO	30K lipoprotein OS Bombyx mori PE 2 SV 1
	LP3_BOMMO	Low molecular 30 kDa lipoprotein PBMHPC 19 precursor
	LP5_BOMMO	Low molecular 30 kDa lipoprotein PBMHPC 23 precursor
29	Q6Q0S8_BOMMO	Major plasma protein 30K Fragment OS Bombyx mori GN 19G PE 4 SV 1
	C7A8A3_BOMMO	Low molecular mass 30 kDa lipoprotein 19G1 OS Bombyx mori PE 2 SV 1
	LP3_BOMMO	Low molecular 30 kDa lipoprotein PBMHPC 19 precursor
30	Q6Q0S8_BOMMO	Major plasma protein 30K Fragment OS Bombyx mori GN 19G PE 4 SV 1

	C7A8A3_BOMMO	Low molecular mass 30 kDa lipoprotein 19G1 OS Bombyx mori PE 2 SV 1
	LP3_BOMMO	Low molecular 30 kDa lipoprotein PBMHPC 19 precursor
31	Q6Q0S8_BOMMO	Major plasma protein 30K Fragment OS Bombyx mori GN 19G PE 4 SV 1
	C7A8A2_BOMMO	30 kDa protein OS Bombyx mori PE 2 SV 1
	C7A8A3_BOMMO	Low molecular mass 30 kDa lipoprotein 19G1 OS Bombyx mori PE 2 SV 1
	LP1_BOMMO	Low molecular 30 kDa lipoprotein PBMHP 6 precursor
	LP3_BOMMO	Low molecular 30 kDa lipoprotein PBMHPC 19 precursor
	LP5_BOMMO	Low molecular 30 kDa lipoprotein PBMHPC 23 precursor
32	Q6Q0S8_BOMMO	Major plasma protein 30K Fragment OS Bombyx mori GN 19G PE 4 SV 1
