# Supplementary material for: A proteomic view on the developmental transfer of homologous 30 kDa lipoproteins from peripheral fat body to perivisceral fat body via hemolymph in silkworm, Bombyx mori
Source: BMC Biochem. 2012 Feb 28;13:5. doi: 10.1186/1471-2091-13-5 (PMC3306753; doi:10.1186/1471-2091-13-5)
Supplement: Additional file 16 — Separation of 30 kDa lipoproteins from B. mori by DEAE ion chromatography followed by 1D-PAGE (bands resulting from fraction 34, Figure 5). Identification by LC-MS/MS. Tentative assignment of tryptic peptides to lipoproteins LP1-LP5, L301/L302. [file 1471-2091-13-5-S16.PDF]

Additional file 16 - Separation of 30 kDa lipoproteins from *B. mori* by DEAE ion chromatography followed by 1D-PAGE (bands resulting from fraction 34, Fig. 5). Identification by LC-MS/MS. Tentative assignment of tryptic peptides to lipoproteins LP1-LP5, L301/L302. Peptides were often found to be deamidated or oxidized. Sequences marked in bold represent terminal peptides.

Sequence	LP1	LP2	LP3	LP4	LP5	L301	L302	C7A8A2	Q17185	A7LIK7	29 kDa	30 kDa	31 kDa
FTPVLENNR	x							x	x		x	x	
SYFPIQFR	x							x	x		x	x	
NTMDFAYQLWTK	x							x	x		x	x	
TDDVLAEQLYMSVVIGEYETAIAK	x							x	x		x	x	
VIFTEQTVK	x										x	x	
IIYGDSTADTFK	x											x	
IAFGDSKDK	x							x	x		x		
EALGHSGEVSGYPQLFAWYIVPY	x										x		
EYNSVMTLDEDMAANEDR	x										x		
GSIIQNVVNNLIIDK		x			x					x	x	x	x
KSEVITNVVNK			x			x					x	x	x
LGSTTNPSNER		x			x						x	x	x
LYNSILTGDYDSAVR		x		x	x		x			x	x	x	x
YENDVLFFIYNR		x		x	x		x			x	x	x	x
IAYGDGVDK		x			x						x	x	x
VVYGGNSADSTR		x			x					x	x	x	x
GSIIQNVVNNLIIDKR					x					x	x	x	
LGPTLDPANER				x			x				x	x	
NSDLISWK				x			x				x	x	
SLEYESQGQGSIVQNVVNNLIIDK		x									x	x	
YFPLNFR					x						x	x	
YFPLSFR		x									x	x	
IAYGDGVDKHTELVSWK					x					x	x	x	
NYNLALK		x		x	x		x				x	x	
QFNDALELGTIVNASGDR		x			x						x	x	
QFNDALELGTIVNASGDRK		x			x						x	x	
QSLEYENQGK				x	x		x			x	x	x	
YDNDVLFYIYNR			x			x						x	x
DCFPVEFR			x			x						x	x
MAWGYNGR			x			x						x	x
LWVGNGQDIVKK		x			x							x	

LYNSILTGDYDSAVRK		x										x	
SLEYESQGQGSIVQNVVNNLIIDKR		x										x	
DRVVYGGNSADSTR		x			x							x	
KSLEYESQGQGSIVQNVVNNLIIDK		x										x	
KYFPLNFR					x							x	
FITLWENNR		x		x	x		x				x		x
EQWFFQPAK		x			x						x		x
VIFGTNTADTTR				x			x				x		x
AVGHDGEVAGLPDIYSWFITPF		x			x						x		
EKNSDLISWK				x			x				x		
GSIIQNVVNNLIIDGSR				x			x				x		
LWVGNGQDIVK		x									x		
LWVGNGQEIVR					x					x	x		
LWVGNGQHIVR				x			x				x		
YFPYNFR				x			x				x		
AQWYLQPAK			x			x					x		
DRVVYGGNSADSTR		x			x						x		
EQWFLQPTK				x			x				x		
EYNDALKLGR				x			x				x		
IVDASGDR				x			x				x		
LIFAENAIK			x			x					x		
LIMAGNFVK				x			x				x		
MAFGHDGEVAGLPDIFSWFVTPF				x			x				x		
SEVITNVVNK			x			x							x
QSLEYESQGK		x			x								x
VIGSPEHYAWGIK			x			x							x
