# Supplementary material for: A proteomic view on the developmental transfer of homologous 30 kDa lipoproteins from peripheral fat body to perivisceral fat body via hemolymph in silkworm, Bombyx mori
Source: BMC Biochem. 2012 Feb 28;13:5. doi: 10.1186/1471-2091-13-5 (PMC3306753; doi:10.1186/1471-2091-13-5)
Supplement: Additional file 17 — Separation of 30 kDa lipoproteins from B. mori by DEAE ion chromatography followed by gel filtration chromatography and 1D-PAGE (band resulting from fraction 60, Figure 5). Identification by LC-MS/MS. Tentative assignment of tryptic peptides to lipoproteins LP1-LP5, L301/L302. [file 1471-2091-13-5-S17.PDF]

Additional file 17 - Separation of 30 kDa lipoproteins from *B. mori* by DEAE ion chromatography followed by gel filtration chromatography and 1D-PAGE (band resulting from fraction 60, Fig. 5). Identification by LC-MS/MS. Tentative assignment of tryptic peptides to lipoproteins LP1-LP5, L301/L302. Peptides were often found to be deamidated or oxidized. The sequence marked in bold represents a terminal peptide. The peptide in italic was also found both phosphorylated (or O-sulfonated) and oxidized.

Sequence	LP1	LP2	LP3	LP4	LP5	L301	L302	C7A8A2	Q6Q0S8
DIVRDCFPVEFR			x			x			
KSEVITNVVNK			x			x			
MNCMEYAYQLWLQGSK			x			x		x	x
SEVITNVVNK			x			x			
YDNDVLFYIYNR			x			x			
DCFPVEFR			x			x		x	x
MAWGYNGR			x			x			
VIGSPEHYAWGIK			x			x			
**ADSDVPNDILEEQLYNSVVVADYDSAVEK**									x
FITLWENNR		x		x	x		x		
LYNSILTGDYDSAVR		x		x	x		x		
DGLALTLSNDVQGDDGRPAYGDGKDK								x	
VVYGGNSADSTR		x		x	x		x		
