# Supplementary material for: A proteomic view on the developmental transfer of homologous 30 kDa lipoproteins from peripheral fat body to perivisceral fat body via hemolymph in silkworm, Bombyx mori
Source: BMC Biochem. 2012 Feb 28;13:5. doi: 10.1186/1471-2091-13-5 (PMC3306753; doi:10.1186/1471-2091-13-5)
Supplement: Additional file 18 — Exemplary spectra for peptides derived from LP2, LP4 and LP5 found in the purified lipoprotein (fraction 60, Figure 5). [file 1471-2091-13-5-S18.PDF]

Additional file 18 - Exemplary spectra for peptides derived from LP2, LP4 and LP5 found in the purified lipoprotein (fraction 60, Fig. 5).

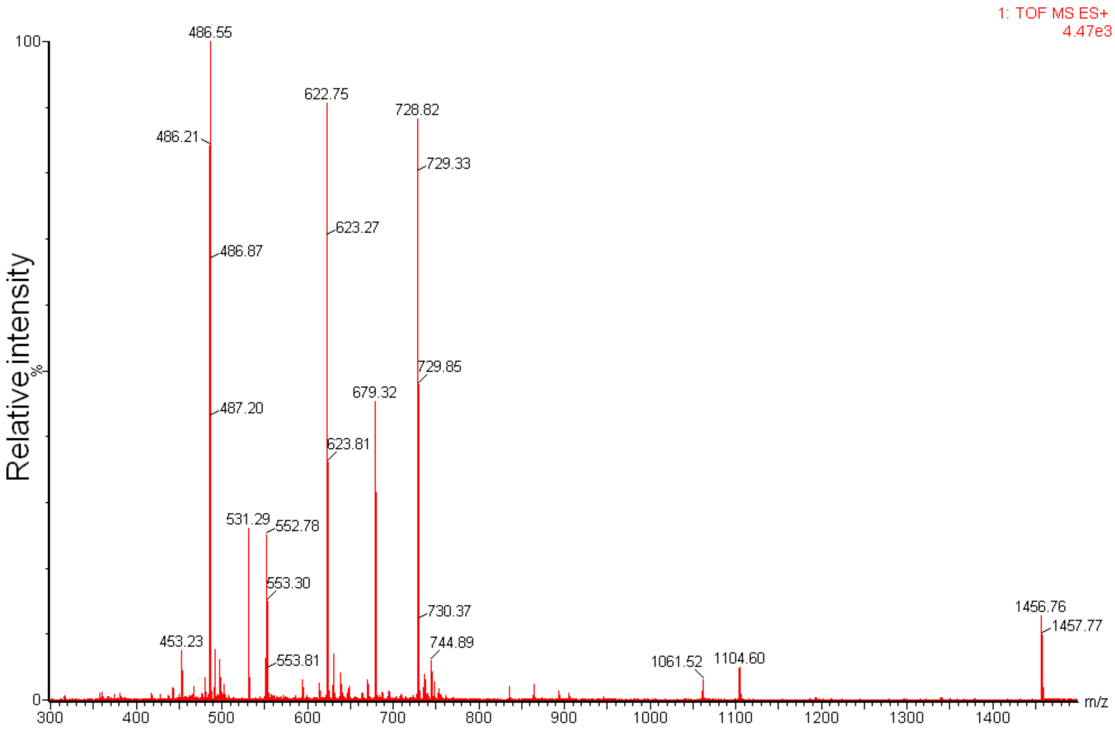

MS spectrum showing the scan with the most intense ion at *m/z* 728.82 subjected to MS/MS fragmentation.

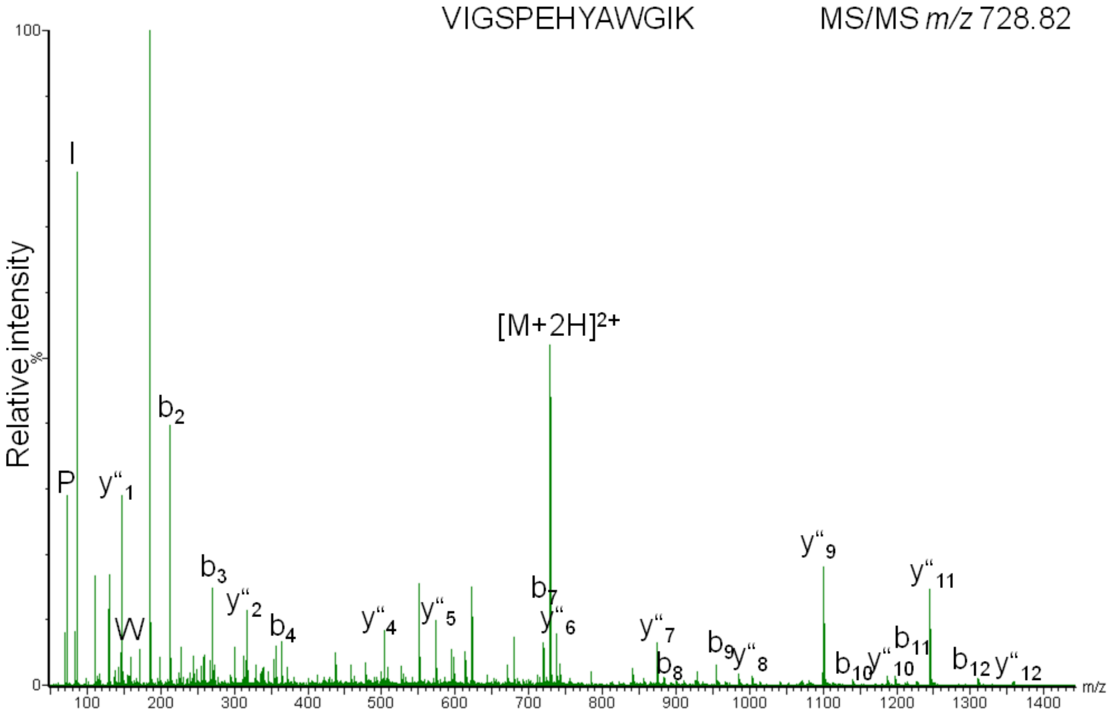

MS/MS of the ion at *m/z* 728.82. All ions of the y-series were observed as well as b and immonium ions. The peptide is unique to LP3/L301.

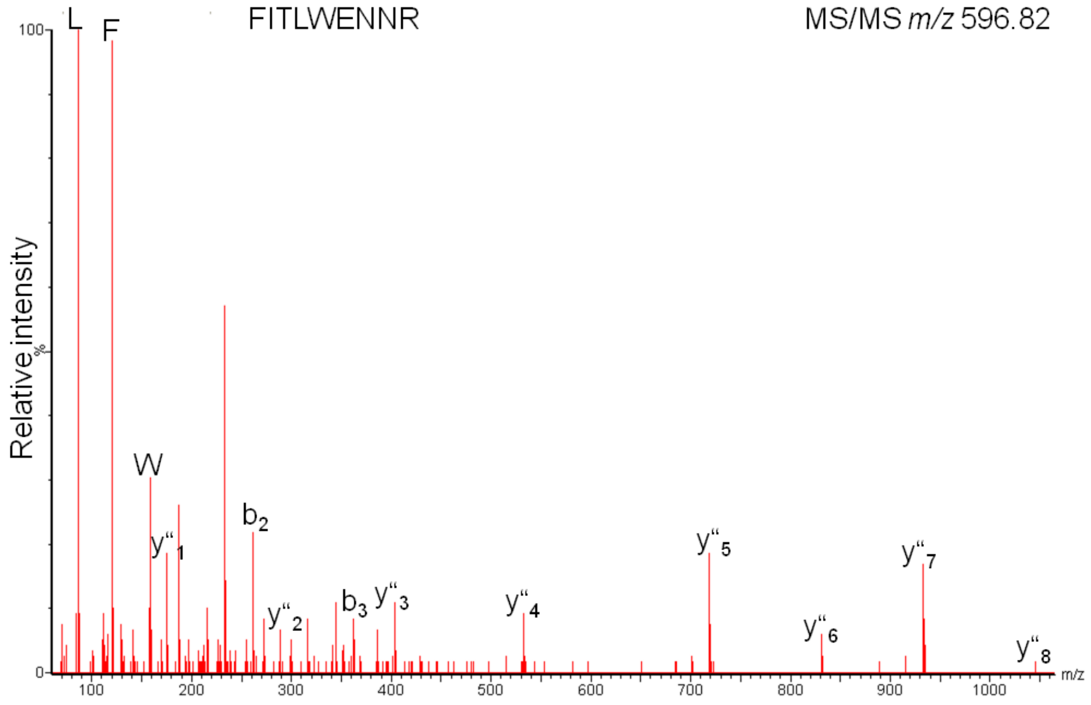

MS/MS spectrum of *m/z* 596.82. Peptide present in LP2, LP4, LP5 and L302 but not in LP3/L301. All ions of the y-series were observed as well as b and immonium ions.

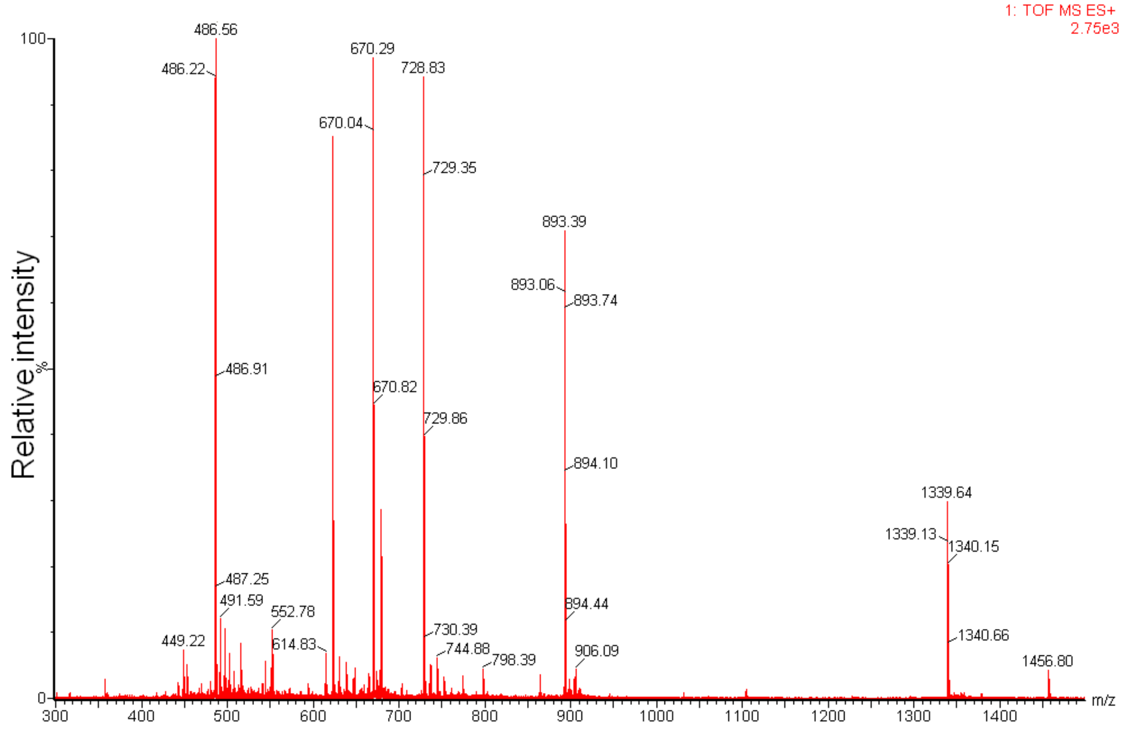

MS spectrum showing the scan with the most intense ion at *m/z* 893.06 subjected to MS/MS fragmentation.

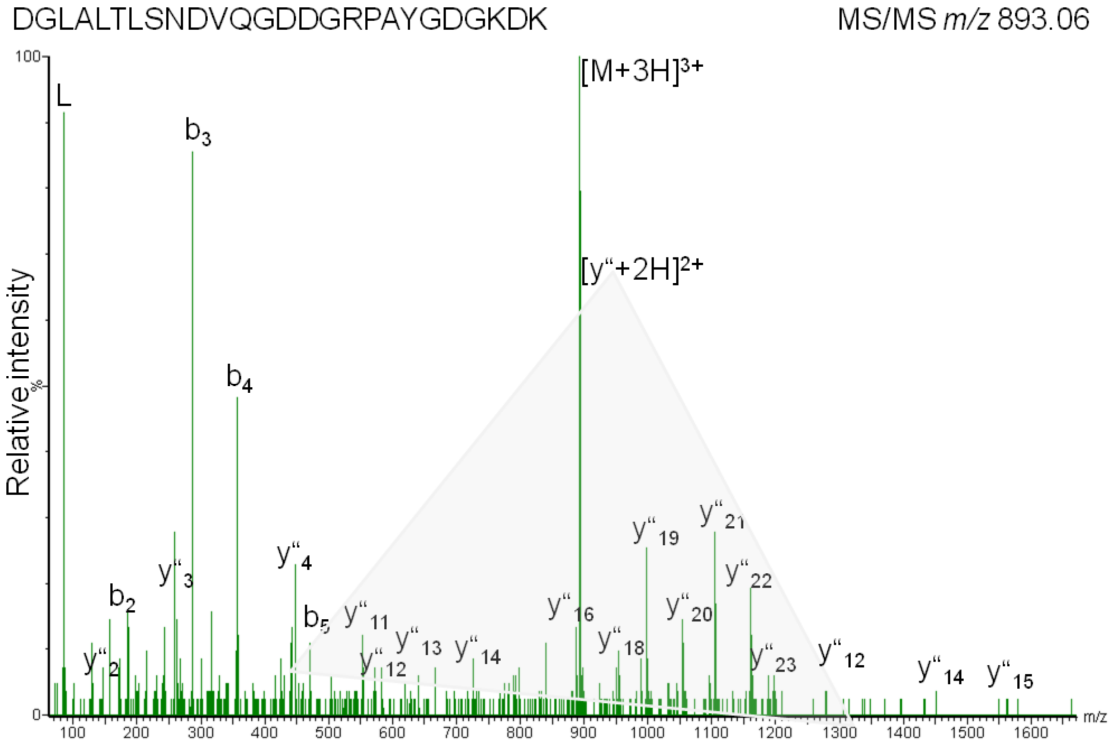

MS/MS of the ion at *m/z* 893.06. All ions of the y-series were observed as well as b and immonium ions. The peptide is unique to C7A8A3.

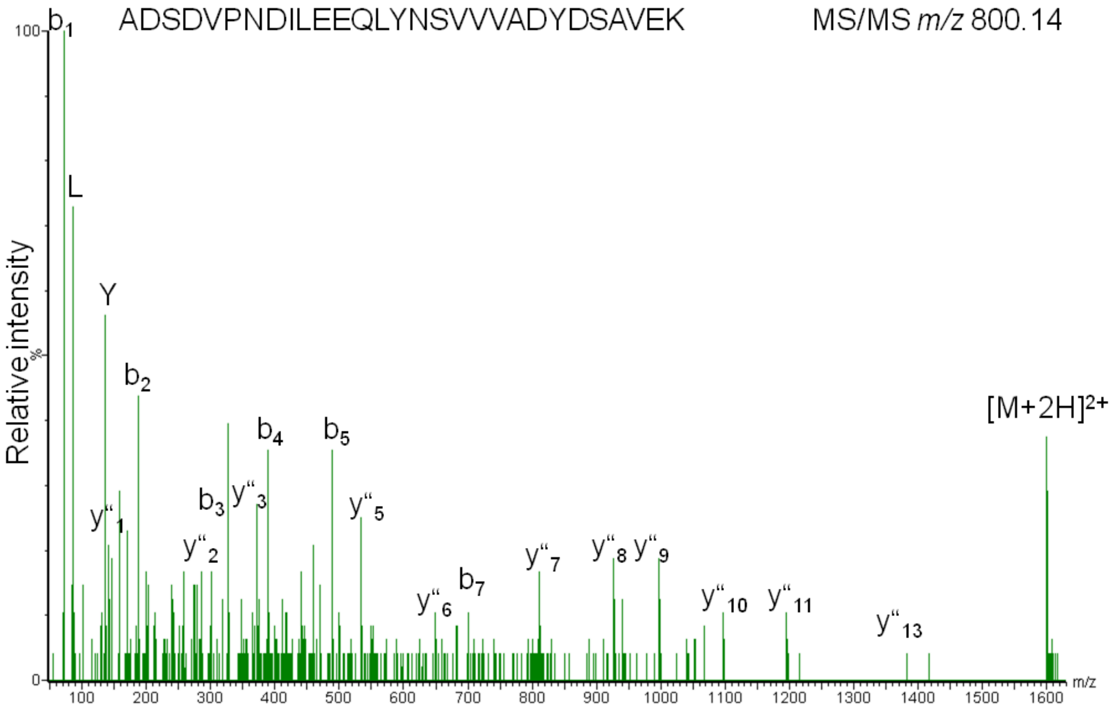

MS/MS of the ion at *m/z* 800.14. All ions of the y-series were observed as well as b and immonium ions. The peptide is N-terminal in Q6Q0S8.

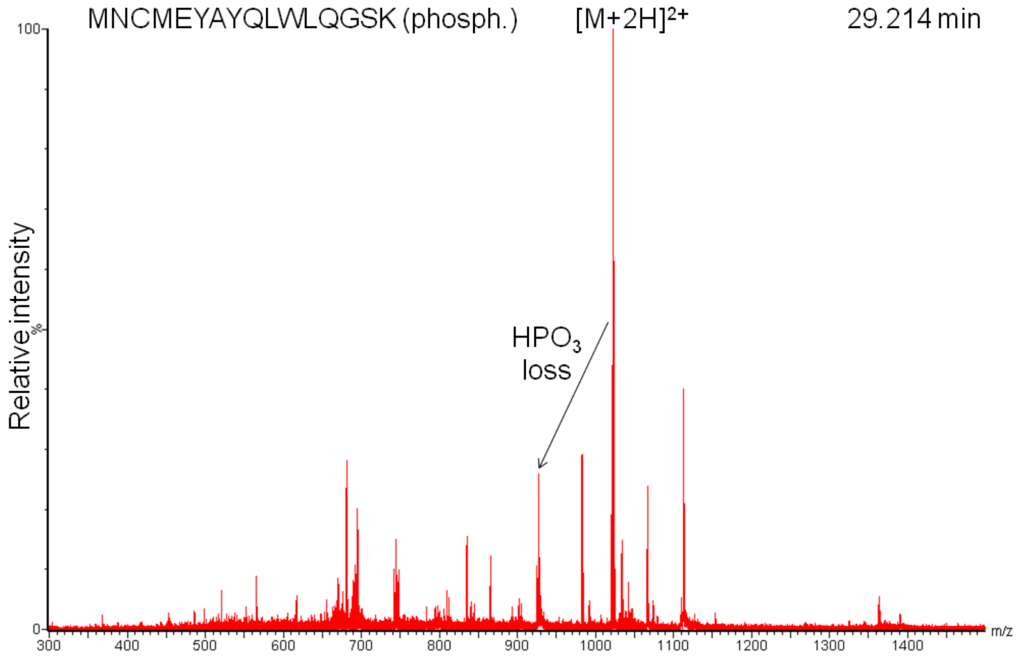

Overview scan (top) and MS/MS spectrum for the phosphorylated (or O-sulfonated) form of a peptide unique in LP3/L301. The peptide easily looses 80 Da. No modified fragment ions can be observed so that the phosphorylation/O-sulfonation site remains ambiguous.
