# Supplementary material for: A proteomic view on the developmental transfer of homologous 30 kDa lipoproteins from peripheral fat body to perivisceral fat body via hemolymph in silkworm, Bombyx mori
Source: BMC Biochem. 2012 Feb 28;13:5. doi: 10.1186/1471-2091-13-5 (PMC3306753; doi:10.1186/1471-2091-13-5)
Supplement: Additional file 19 — Amino acid analysis of purified protein. [file 1471-2091-13-5-S19.PDF]

Additional file 19 - Amino acid analysis of purified protein. Lyophilized protein (20 mg) was dissolved in 6 ml of 6 N HCl and subjected to hydrolysis in a boiling water bath for 24 h. The hydrolysate was centrifuged at 35.000 rpm for 15 min and the supernatant was filtered and neutralized with 1N NaOH. The filtered solution was diluted 1:100 with milliQ water. Amino acid analysis was performed using HP1100 LC system (Agilent, USA) as described before [1] using a flow rate of 0.5 ml/min (40 °C, 338 nm). Mobile phase A consisted of 20 mM sodium acetate containing 0.018% triethylamine and mobile phase B was 20% of 100 mM sodium acetate / 40% methanol / 40% acetonitrile (pH 7.2; adjusted in each case with 2% acetic acid). For 30 kDa protein amino acid analysis, 10 µl of the hydrolysed proteins (1 mg/ml) was mixed with 60 µl 0.4 N borate buffer (pH 10.4) and 10 µl orthopthalaldehyde (10 mg/ml in 0.4 N borate buffer, OPA; Hewlett Packard). This mixture (50 µl) was injected per run. Hewlett Packard standard amino acid mixture in different concentrations (1 nmol, 10, 25, 250 pmol) was used to calibrate the detector response.

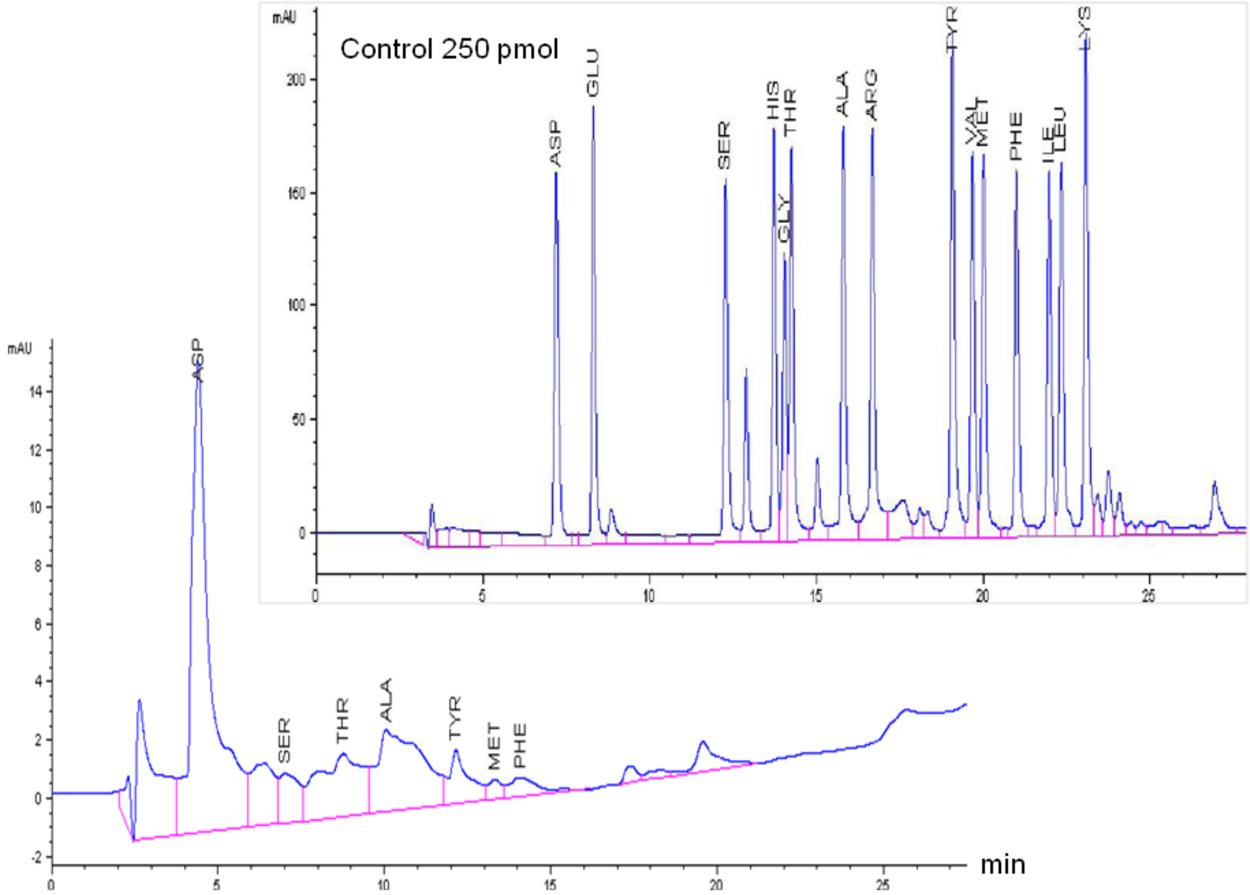

Amino acids	µmoles/ml
Aspartic acid	3.7
Glutamic acid	9.5
Serine	1.9
Histidine	2.8
Glycine	0.6
Threonine	3.7
Alanine	5.9
Arginine	2.1
Tyrosine	2.8
Valine	0.9
Methionine	0.3
Phenylalanine	1.0
Isoleucine	0.0
Leucine	0.0
Lysine	0.0

1. Brückner H, Wittner R, Godel H: Fully automated HPLC separation of DL amino acids derivatized with OPA together with N-isobutyrl-cystine. Applications to food samples. *Chromatographia* 1991, 32:383-388.
